# Chemometric Optimization of BF_3_·OEt_2_‐Mediated Cyclization of Cannabidiol to Rare Δ⁴‐ and *Iso*‐THC Isomers

**DOI:** 10.1002/chem.202502387

**Published:** 2025-10-30

**Authors:** Arianna Bini, Lisa Rita Magnaghi, Valeria Cavalloro, Alessandra Bonanni, Stefano Protti, Daniele Merli

**Affiliations:** ^1^ Department of Chemistry University of Pavia Viale Taramelli 10 Pavia 27100 Italy; ^2^ Department of Earth, Environmental Sciences Via A. Ferrata 1 Pavia 27100 Italy

**Keywords:** cannabidiol (cbd), design of experiments (doe), lewis acid catalysis, principal component analysis (pca), tetrahydrocannabinol derivatives (thcs)

## Abstract

The acid‐catalyzed conversion of cannabidiol (CBD) to tetrahydrocannabinol and iso‐tetrahydrocannabinol derivatives is a well‐established synthetic strategy. However, since the reaction outcome is strongly dependent on the conditions, a careful investigation is always required to achieve the optimal chemoselectivity. Chemometrics recently emerged as an effective approach for improving synthetic methods, especially when multiple parameters are involved. The present paper aims to apply chemometrics tools to the optimization of the procedures for the preparation of Δ⁹‐THC, Δ⁸‐THC, Δ⁸‐*iso*‐THC, Δ⁴‐*iso*‐THC, and Δ⁴^(^⁸^)^‐*iso*‐THC. All the reactions have been performed at room temperature by tuning the initial concentration of CBD, the equivalents of the model Lewis acid considered (BF_3_·OEt_2_), the reaction time, and the nature of the media to achieve the desired products. The kinetics of the process, followed over the course of 1 to 48 hours, were analyzed by means of Principal Component Analysis (PCA) to initially simplify the multidimensional dataset and help identify the best media for Δ⁹‐THC and Δ⁸‐THC. In order to optimize the conditions needed for Δ⁴‐*iso*‐THC, Δ⁴(⁸)‐*iso*‐THC, and Δ⁸‐*iso*‐THC, multiple Design of Experiment (DoE) were employed, leading to the successful isolation of the target compounds. During the investigation, cannabinoid derivatives incorporating a portion of the reaction medium were also identified.

## Graphical abstract

Find the path! The present paper points out the potentialities of chemometrics tools to design selective BF_3_·OEt_2_ catalyzed procedures for the preparation of Δ⁹‐THC, Δ⁸‐THC, Δ⁸‐*iso*‐THC, Δ⁴‐*iso*‐THC, Δ⁴^(^⁸^)^‐*iso*‐THC, by simply tuning the initial concentration of CBD, the amount of the Lewis acid, the reaction time as well as the nature of the solvent employed.

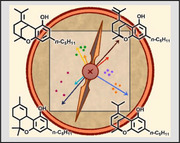

## 1 Introduction

Among the cannabinoids naturally occurring in *Cannabis sativa* L. chemotypes, Cannabidiol (CBD) has attracted growing scientific interest due to its broad spectrum of pharmacological activities.^[^ ^1^, ^2^ ^]^ Although both bicyclic CBD and tricyclic Δ⁹‐tetrahydrocannabinol (Δ⁹‐THC, the other predominant phytocannabinoid) interact with cannabinoid receptors CB_1_ and CB_2_, they elicit markedly different physiological responses. CBD is nonpsychotropic and has shown therapeutic potential for treating Parkinson's disease,^[^ ^3^ ^]^ epilepsy,^[^ ^4^ ^]^ cancer,^[^ ^5^ ^]^ and related disorders. Conversely, Δ⁹‐THC, while effective against chemotherapy‐induced nausea,^[^ ^6^ ^]^ Alzheimer's disease,^[^ ^7^ ^]^ and multiple sclerosis,^[^ ^8^ ^]^ exerts psychotropic effects such as euphoria, sedation, and dizziness.^[^ ^9^ ^]^

Apart from pharmaceutical applications, CBD has served in the last decades as a multifaceted precursor for structurally diverse bioactive compounds.^[^ ^10^, ^11^, ^12^, ^13^, ^14^ ^]^ In this context, cyclization under acid conditions has been widely investigated and exploited for the preparation of Δ⁷‐, Δ⁸‐, Δ⁹‐, Δ^10^‐, Δ^1^ ^1^‐THC, as well as the corresponding *iso*‐THCs depending on the nature of both the solvent and the catalyst.^[^ ^15^, ^16^, ^17^, ^18^, ^19^ ^]^ The catalysts employed in such processes range from Brønsted (e.g., TFA, pTSA, HCl)^[^ ^18^, ^20^ ^]^ to Lewis acids (e.g., ZnBr_2_, TMSOTf, BF_3_·OEt_2_),^[^ ^8^, ^20^ ^]^ while different reaction media have been considered, including, among the others, acetonitrile,^[^ ^18^ ^]^ acetone,^[^ ^20^ ^]^ haloalkanes,^[^ ^18^, ^21^ ^]^ as well as aromatics^[^ ^18^ ^]^ and hydrocarbons.^[^ ^21^ ^]^ The use of alternatives to conventional thermal approaches, such as ultrasonic‐ or microwave‐assisted continuous flow chemistry, has also been taken into account.^[^ ^21^ ^]^ Nonetheless, the impressive sensitivity of CBD to the reaction conditions, along with the smooth interconversion among THC isomers, poses a challenge to the development of efficient and chemoselective protocols and requires a careful reaction design.

In this context, chemometrics is becoming increasingly recognized as an effective strategy for enhancing synthetic methods, particularly when the results may be influenced by several factors. Defined as the application of mathematical, statistical, and logical techniques to extract relevant chemical information from complex datasets,^[^ ^22^ ^]^ chemometrics thus offers a multivariate framework for experimental design in synthetic organic chemistry.^[^ ^23^ ^]^ Such methodologies are particularly useful for identifying which variables, among many potentially influential factors, actually affect reaction outcomes.^[^ ^24^ ^]^ Once key factors are identified, chemometric tools such as Design of Experiments (DoE) can be employed to systematically optimize reaction conditions.^[^ ^25^ ^]^ DoE has been successfully applied across various domains, including synthesis,^[^ ^26^, ^27^, ^28^ ^]^ in the preparation of the oral androgen receptor antagonist apalutamide,^[^ ^29^ ^]^ the optimization of light‐mediated route to diarylketones,^[^ ^30^ ^]^ the development of a bioderived photocatalyst for environmental purposes,^[^ ^31^ ^]^ as well as the application of superheated flow conditions.^[^ ^32^ ^]^

In cannabinoid research, chemometric techniques have been used primarily for profiling and classification purposes^[^ ^33^, ^34^, ^35^, ^36^ ^]^ but have yet to be explored in the context of synthetic optimization. The present study aims to fill this gap by applying chemometric tools to optimize the synthesis of selected THC isomers—namely Δ⁹‐THC, Δ⁸‐THC, Δ⁸‐*iso*‐THC, Δ⁴‐*iso*‐THC, Δ⁴^(^⁸^)^‐*iso*‐THC—as well as derivatives that incorporate the reaction medium, via the mild, acid‐catalyzed cyclization of CBD at room temperature by tuning different parameters such as the equivalents of model Lewis acid (BF_3_·OEt_2_), the reaction time and the nature of the media.

Principal Component Analysis (PCA) was first used to simplify the multidimensional dataset and identify trends in product distribution. The resulting insights informed the design of subsequent DoE strategies aimed at selectively enhancing the yield of the more challenging isomers.

## 2 Results and Discussion

**Scheme 1:**
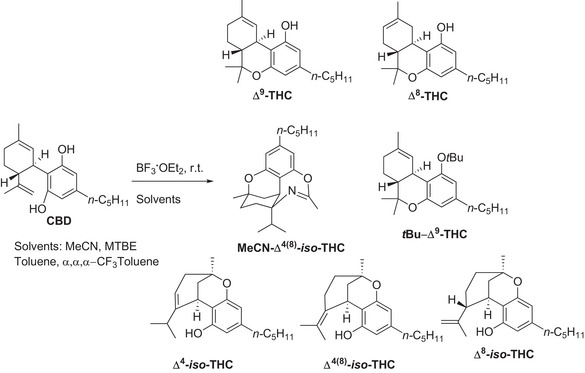
Acid catalyzed cyclization of CBD in the presence of BF_3*_Et_2_O in different media.

Experimental investigation of the reactivity of commercially available CBD in the presence of BF_3_·OEt_2_ has been carried out in a set of solvents, namely acetonitrile, methyl‐tertbutyl ether (MTBE), toluene, and α,α,α‑trifluorotoluene, has been investigated in detail. The reaction has been initially carried out on an exploratory scale (0.032 M, 0.11 M and 0.20 M) and the consumption of CBD as well as the yields of products have been quantified by means of GC‐MS analyses; then, in the most promising cases, a preparative reaction (0.95 mmol, see experimental part and Supporting Information for further details) was performed to isolate and fully characterize the products. An overview of the CBD‐derived compounds described in the present paper is available in Scheme 1, while a detailed description of the reactivity of CBD under the tested conditions has been included in the Supporting Information (see Figures S9–S32).

**Scheme 2:**
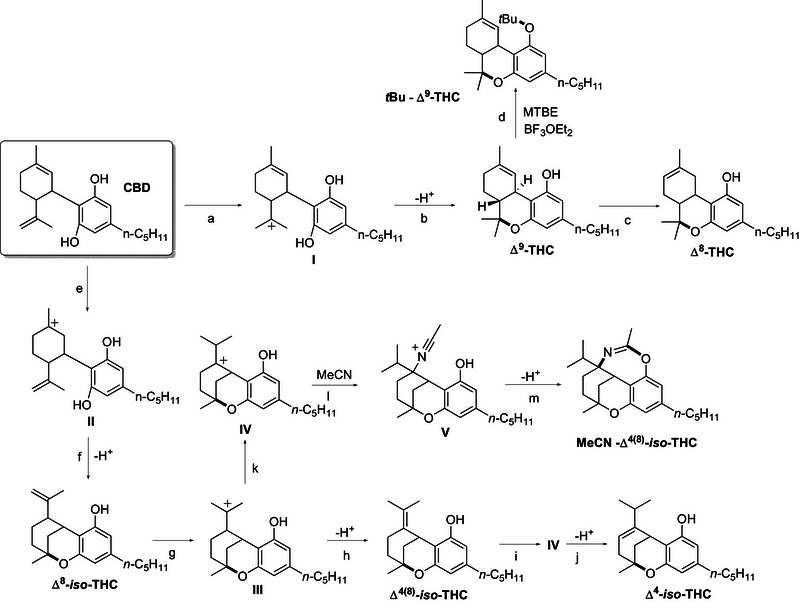
Reaction mechanism for the acid‐catalysed intramolecular cyclization of CBD.

Specifically, in α,α,α‑trifluorotoluene, the formation of Δ⁸‑THC (up to 82% yield, in 6 h with 0.032 mmol of CBD, 5.1 equiv of BF_3_·OEt) was favored, whereas MTBE selectively promoted Δ⁹‑THC (12–69% yield) alongside tert‐butylated product *t*Bu‐Δ^9^‑THC (up to 19% yield). In toluene, a mixture of Δ⁸‑THC (main product), Δ⁴‑*iso*‑THC and Δ⁴^(^⁸^)^‐*iso*‑THC was observed (Figures S9–S14). In contrast, in MeCN we found Δ⁸‑THC, Δ⁴‑*iso*‑THC, Δ⁸‑*iso*‑ and Δ⁴^(^⁸^)^‐*iso*‑THC, along with an *iso*‐THC derivative incorporating a molecule of solvent (dubbed as MeCN‐Δ⁴^(^⁸^)^‐*iso*‑THC) that was observed as the main product. Based on the results obtained and on the available literature,^[^ ^18^ ^]^ the reactivity of CBD under the investigated conditions has been summarized in Scheme 2. Activation of the starting substrate by BF_3_·OEt_2_ results in the competing formation of carbocations **I** (path a) and **II** (path e). Intramolecular nucleophilic addition occurring in **I** results in the formation of Δ⁹‐THC (path b), which can isomerize, under Lewis Acid catalysis to the regioisomer Δ⁸‐THC (path c).^[^ ^37^ ^]^ When the reaction is performed in Methyl‐Tert‐Butyl Ether, acid activation of the medium^[^ ^38^ ^]^ allows for the tertbutylation of the phenolic moiety in Δ⁹‐THC, to afford *t*Bu‐Δ⁹‑THC as the minor product (path d). On the other hand, cyclization of **II** affords Δ⁸‐*iso*‑THC (path f), which in turn generates tertiary carbocation **III**, for which deprotonation led to Δ⁴^(^⁸^)^‐*iso*‑THC (path h) and, after regioisomerization, Δ⁴‐*iso*‐THC (paths i, j). In acetonitrile, **III** could also be converted to **IV** via hydride shift (path k) and addition of a molecule of MeCN to the corresponding carbocation in a Ritter‐type fashion, and subsequent cyclization (paths l, m) affords oxazepine MeCN‐Δ⁴^(^⁸^)^‐*iso*‑THC.

### 2.1 PCA on the Explorative Reactions

The yields (obtained via GC‐MS analyses) from the exploratory reactions performed under the extreme reaction conditions described in Table 1 (Entries a–d) were submitted to PCA to visualize the correlation between experimental conditions and obtained products. This chemometric tool, seldom exploited in this field, allows to reduce datasets dimensionality and to extract correlations between parameters used to describe the samples. The information referring to the samples, that is, similarity or dissimilarity among them, is summarized by computing the directions of highest variability of the samples, namely PC1 and PC2 in this case, as a linear combination of the original variables, the GC‐MC yields. This means that we can visualize in the score plot how the original samples, reaction conditions, are located and thus the similarity or dissimilarity among them; additionally, we can extract which original variables are mainly responsible for the samples location and thus similarity/dissimilarity relying on the loadings plot. To summarize both aspects, in this case we jointly plotted samples location alongside the new directions (scores) and variable influence (loading).

**Table 1:** Codification of the extreme values (in term of equiv. of Lewis Acid) for the PCA training set (conditions from a to d) and test set (conditions e and f) used for the validation.

[M of CBD–BF_3_·OEt_2_ eq]	
a	0.032–1.2
b	0.032–5.1
c	0.20–1.2
d	0.20–5.1
*e*	*0.11–1.2*
*f*	*0.11–5.1*

**Figure 1:**
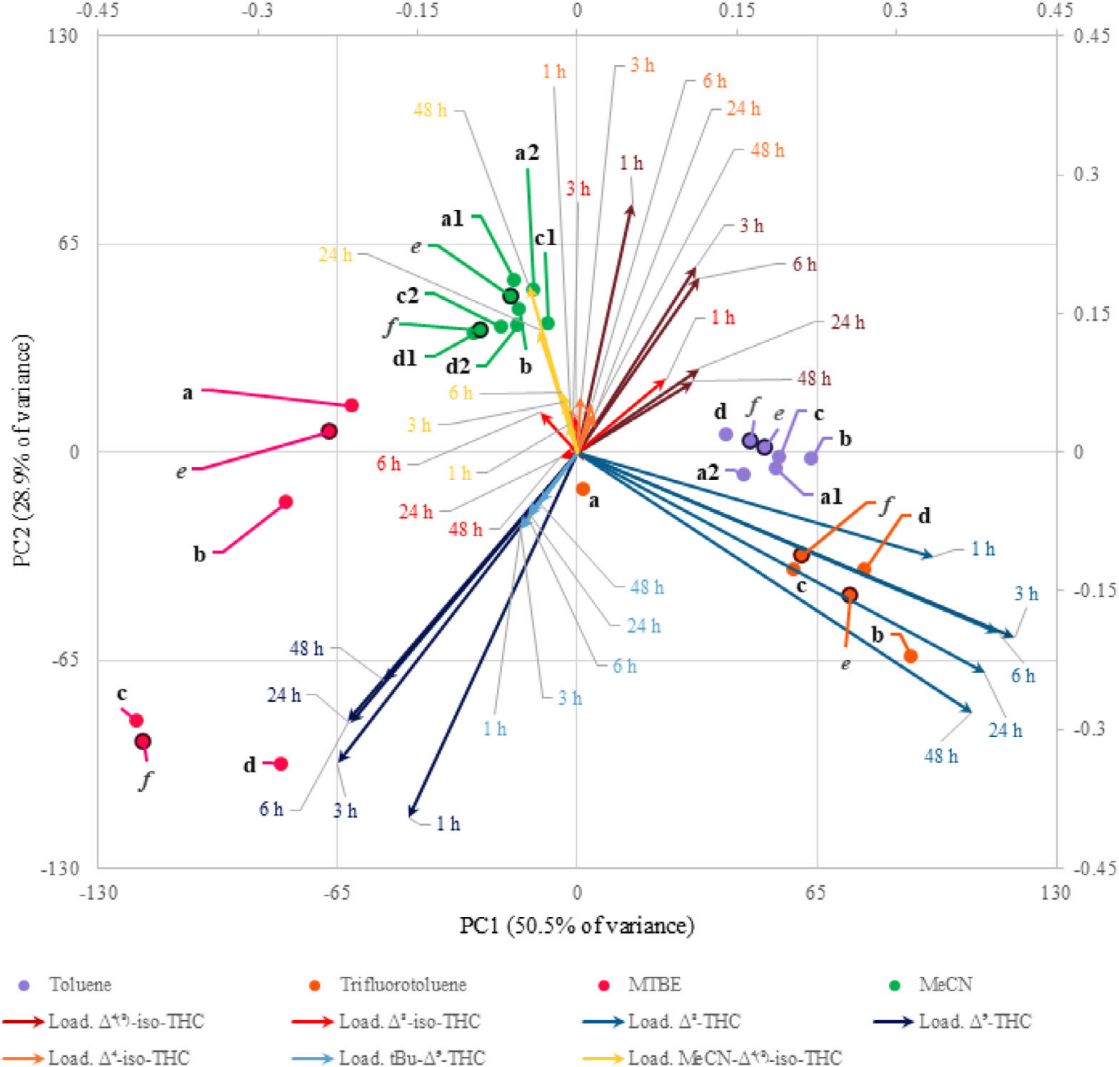
Biplot containing both the loadings and scores obtained for each kinetic acquired; the reaction conditions e and f are relative to the GC‐MS yield achieved in intermediate conditions.

The resulting biplot (Figure 1) emphasizes that the choice of the solvent has a key role in determining the product distribution. Specifically, while α,α,α‑trifluorotoluene (orange points) favored Δ⁸‑THC formation (blue arrows), the same reaction performed in MTBE (red points) mainly promoted the formation of Δ⁹‑THC (dark blue arrows) alongside the derivative labelled as *t*Bu‐Δ⁹‑THC (light blue arrows), where a *t*Bu moiety has been incorporated. Notably, when performing the reaction in acetonitrile (green points), a compound deriving from the acid‐catalyzed addition of the solvent to Δ⁴^(^⁸^)^‑*iso*‑THC (dark red arrows) with a Ritter‐type reaction was detected (yellow arrows). Such optimal conditions were then scaled up to isolate the respective compounds (see Preparative Experiments). This preliminary overview was further confirmed by projected GC‐MS yields achieved in intermediate conditions (Table 1, Entries e,f) onto the previously developed model (Figure 1): the location of projected samples confirmed the fundamental role of solvent and the minor contribution of other conditions in determining the products obtained. Optimization of Δ⁴^(^⁸^)^‐*iso*‑THC and Δ⁸‐*iso*‑THC proved more complex, as both isomers are formed in acetonitrile and toluene. Consequently, further systematic investigation on the reaction conditions was pursued to enhance yields and facilitate isolation.

### 2.2 Design of Experiment (DoE)

We identified Δ⁴^(^⁸^)^‐*iso*‑THC and Δ⁸‐*iso*‑THC as the most challenging products to synthesize. To enhance their yields, a 2^3^ full factorial DoE was implemented: the investigated variables and corresponding levels are summarized in Table 2, while the overall list of experiments performed is described in Table 3. Δ⁴^(^⁸^)^‐*iso*‑THC and Δ⁸‐*iso*‑THC yields, obtained in the experimental conditions listed in Table 3 after 1 and 3 hours, were used as a training set to build polynomial models, whose general formula is reported in Equation 1, able to correlate products yields with variables levels. The computation of b coefficients in Equation 1 is achieved by Multiple Linear Regression (MLR), together with confidence intervals and significance, shown in Figures S33, S34 and Tables S1, S2.

(1) $$\begin{array}{ccc} & & GC-\text{MS yield}=b_{0}+b_{1}\times Solv+b_{2}\times CBD_{conc}\\ & & \;\;+\;b_{3}\times BF_{3}\cdot OE{t_{2}}_{eq}+b_{12}\times Solv\times CBD_{conc}\\ & & \;\;+\;b_{13}\times Solv\times BF_{3}\cdot OE{t_{2}}_{eq}+b_{23}\times CBD_{conc}\times BF_{3}\cdot OE{t_{2}}_{eq} \end{array}$$

**Table 2:** Level definition for the parameters considered in the Full Factorial Design, 2^3^.

Variable	Minimum level [‐1]	Maximum level [1]
Solvent	Toluene	MeCN
CBD concentration (M)	0.032	0.20
BF_3·_·OEt_2_ equivalents	1.2	5.1

**Table 3:** Experimental plan and responses (GC‐MS yield) used to compute the coefficients by MLR.

Training experiment list			1h [GC‐MS yield]		3h [GC‐MS yield]	
Solvent	CBD [M]	BF_3_·OEt_2_ eqv	Δ^4(8)^‐*iso*‐THC	Δ^8^‐*iso*‐THC	Δ^4(8)^‐*iso*‐THC	Δ^8^‐*iso*‐THC
Toluene	0.032	1.2	15.1	36.0	31.2	10.7
Toluene	0.032	1.2	9.1	41.0	23.3	10.2
MeCN	0.032	1.2	39.2	39.2	44.0	14.8
MeCN	0.032	1.2	27.1	41.6	44.4	14.3
Toluene	0.20	1.2	34.1	11.0	29.1	0.0
MeCN	0.20	1.2	31.7	4.3	23.9	0.0
MeCN	0.20	1.2	35.4	15.6	27.0	2.8
Toluene	0.032	5.1	33.3	8.5	34.9	1.3
MeCN	0.032	5.1	43.4	0.0	35.7	0.0
Toluene	0.20	5.1	29.5	10.4	36.0	0.0
MeCN	0.20	5.1	38.7	0.0	13.0	0
MeCN	0.20	5.1	41.7	1.4	20.1	0

In some cases, due to reproducibility issues, experiments have been performed in duplicate and separately considered for model construction, as keeping both provides more information to the model. For Δ⁴^(^⁸^)^‐*iso*‐THC at 1 hour, only the solvent (acetonitrile) significantly impacted yield, with BF_3_·OEt_2_ equivalents trending just below statistical significance—favoring the higher level. At 3 hours, the low CBD concentration became relevant, while solvent and high Lewis acid loading remained optimal to capture significant interaction effects.

In contrast, the Δ⁸‐*iso*‐THC data indicate that low concentration of CBD (0.032 M) and a slightly stoichiometric excess of BF_3_·OEt_2_ (1.2 equiv.) result in maximal yield. These factors also significantly interact. Although solvent alone was not significant, the solvent × BF_3_·OEt_2_ interaction approached significance, suggesting acetonitrile as the preferred medium to exploit this beneficial effect.

Given the distinct optimal conditions for each isomer, two dedicated face‐centered designs (FCDs) were developed, both conducted in acetonitrile. This latter approach allows to develop more complex polynomial models showing all the linear and 2‐interaction terms, similarly to Equation 1, but also yields’ quadratic dependence from the variables, expressed as b_nn_x_n_^2^ terms. The general formula for these models is presented in Equation 2.

(2) $$\begin{array}{ccc} & & GC-\text{MS yield}=b_{0}+b_{1}\times CBD_{conc}+b_{2}\times BF_{3}\cdot OE{t_{2}}_{eq}\\ & & \;\;+\;b_{12}\times CBD_{conc}\times BF_{3}\cdot OE{t_{2}}_{eq}+b_{1}^{2}\times CBD_{conc}^{2}\\ & & \;\;+\;b_{2}^{2}\times BF_{3}\cdot OE{t_{2}}_{2}^{2} \end{array}$$

The FFD‐informed training experiment lists are shown in Tables 2, 3. For Δ⁴^(^⁸^)^‐*iso*‐THC, the FFD highlighted a pronounced requirement for higher BF_3_·OEt_2_ equivalents and exhibited a sign reversal for CBD concentration. Therefore, we retained the original CBD range with a central point at 0.11 M and extended the Lewis Acid range to include even higher equivalents. Conversely, for Δ⁸‐*iso*‐THC, FCD investigations were bounded by the maximum levels identified in the FFD, retaining low CBD and BF_3_·OEt_2_ settings.

Notably, kinetic analysis (Figure S33) for Δ⁴^(^⁸^)^‐*iso*‐THC revealed that the CBD concentration coefficient shifted from positive at 1 hour to negative at 3 hour. To capture this transition, an additional sampling point at 2 hour was included. For both FCDs, three replicates at the factorial center ([0, 0]) were conducted to assess experimental variance and the results have been separately reported in Tables 4, 5.

**Table 4:** Experimental plan and experimental responses (GC‐MS yield) used to compute the coefficients by MLR.

CBD [M]	BF_3·_OEt_2_ equiv.	1h	2h	3h
0.032	5.1	53.4	36.6	38.0
0.20	5.1	19.6	11.0	14.4
0.032	10.1	29.5	22.2	17.4
0.20	10.1	13.5	10.5	6.7
0.032	7.6	31.4	32.8	23.0
0.20	7.6	32.5	16.0	10.7
0.11	5.1	35.5	17.5	12.6
0.11	10.1	20.3	12.2	7.3
0.11	7.6	25.9	18.9	17.5
0.11	7.6	24.0	16.3	14.7
0.11	7.6	24.6	17.9	9.5

**Table 5:** Experimental plan and responses (GC‐MS yield) used to compute the coefficients by MLR.

CBD (M)	BF_3_·OEt_2_ equiv.	1h	2h	3h
0.016	0.6	66.2	82.5	84.8
0.032	0.6	85.7	89.4	85.3
0.016	1.2	79.0	68.0	75.7
0.032	1.2	50.8	29.0	22.2
0.016	0.9	77.2	79.1	70.8
0.032	0.9	87.3	76.3	68.0
0.024	0.6	85.6	90.2	81.9
0.024	1.2	76.4	52.7	46.8
0.024	0.9	89.0	87.1	81.7
0.024	0.9	88.6	86.7	85.4
0.024	0.9	92.8	86.4	85.2

### 2.3 Face Centered Design for the Optimization of the Yield of Δ^4(8)^‐*iso*‐THC

**Figure 2:**
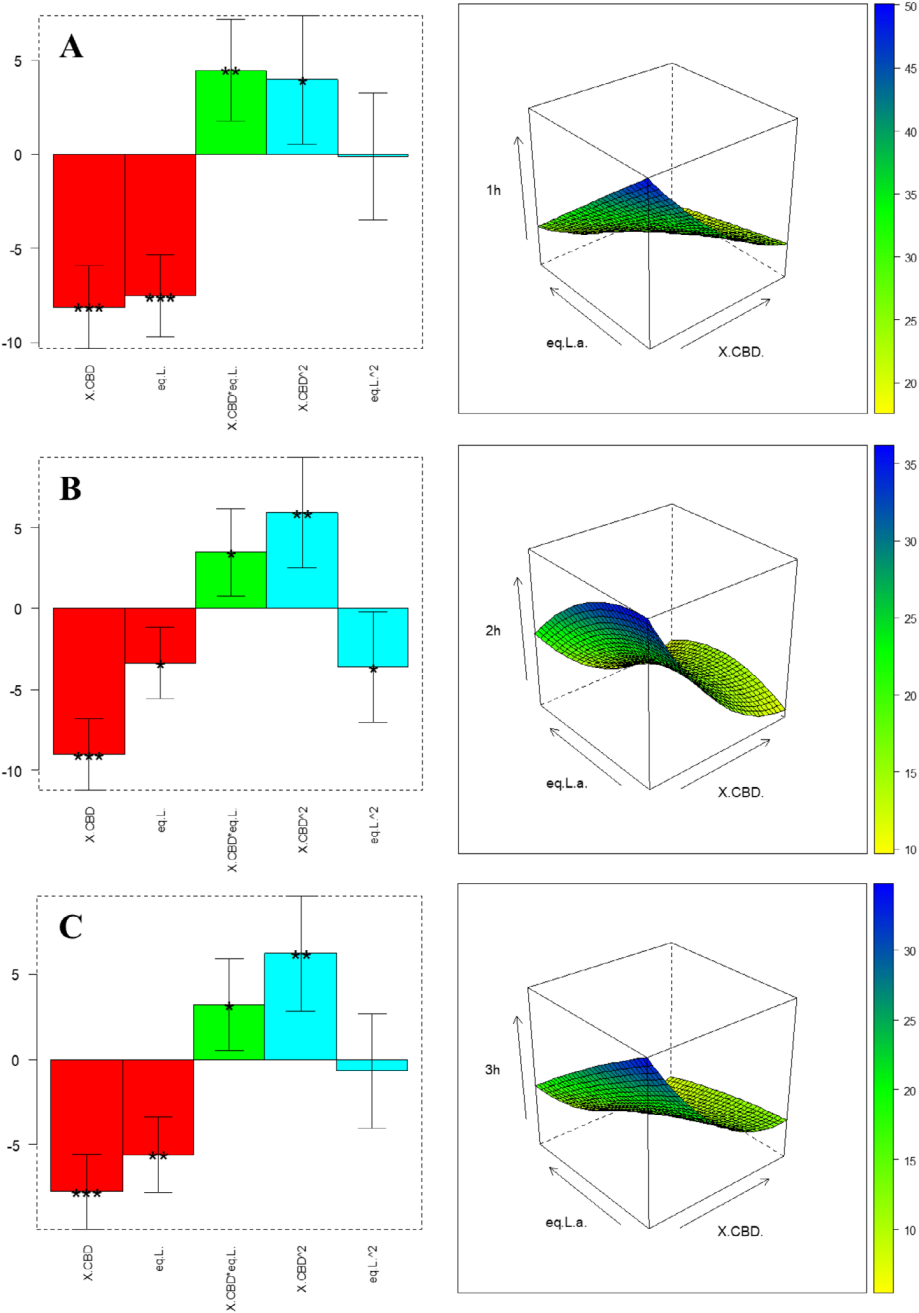
Coefficient plots and response surface of the interaction between CBD concentration and BF_3·_OEt_2_ equivalents relative to the FCD on Δ^4(8)^‐*iso*‐THC, respectively: A) 1 hour, B) 2 hours, and C) 3 hours of reaction time. When present, the asterisks indicate a significant influence of the respective parameters or their interaction and significance (* p ≤ 0.05, ** p ≤ 0.01, *** p ≤ 0.001).

The experiments in Table 4 (with level definition in Table 6) are used to compute the coefficients in Equation 2; the results are shown in bar plots in Figure 2 and Table S4: Both factors significantly influence yield at 1 hour and 2 hour; at 3 hour, only CBD concentration remains significant. All coefficients are negative, implying that the lower level for both factors—0.032 M CBD and 5.1 equiv BF_3_·OEt_2_—maximizes Δ⁴^(^⁸^)^‐*iso*‑THC yield. Under these conditions, the interaction between CBD and catalyst is also favorable, as evidenced by response‐surface plots. Additionally, monitoring b_0_ over time reveals decreasing baseline yield, suggesting product degradation at longer times. Thus, 1 hour is the optimal reaction duration. An independent validation experiment was performed at an untested point: 0.072 M CBD and 6.1 equiv BF_3_·OEt_2_ (matrix coordinates [‐0.5, ‐0.6]). Experimental yield, calculated with pooled standard deviation from central replicates, fell within the model's predicted confidence interval—confirming validity, as displayed in Table S3.

**Table 6:** Level definition of the parameters considered in the FCD for the optimization of Δ^4(8)^‐*iso*‐THC yield in acetonitrile.

FCD for the optimization of Δ^4(8)^‐*iso*‐THC yield			
*Variable*	*Minimum level [‐1]*	*Intermediate level[0]*	*Maximum level [1]*
*CBD concentration (M)*	0.032	0.11	0.20
*BF_3·_* ·*OEt_2_ equivalents*	5.1	7.6	10.1

### 2.4 Face Centered Design for the Optimization of the Yield of Δ^8^‐*iso*‐THC

**Figure 3:**
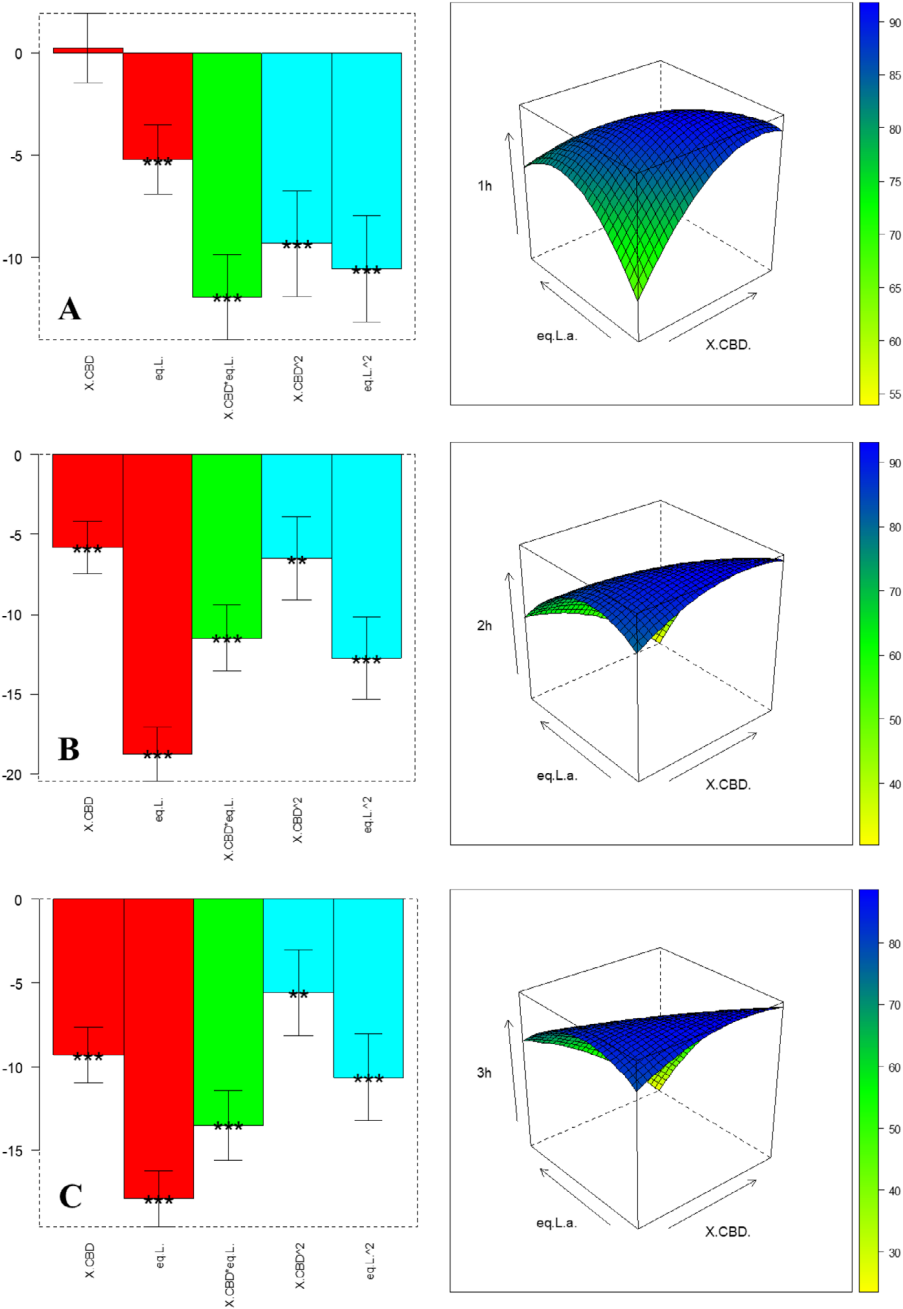
Coefficient plots and response surface of the interaction between CBD concentration and BF_3_·OEt_2_ equiv. relative to the FCD on Δ^8^‐*iso*‐THC, respectively: A) 1 hour, B) 2 hours, and C) 3 hours of reaction time. The asterisks, when present, indicate a significant influence of the respective parameters on their interaction and significance (* p ≤ 0.05, ** p ≤ 0.01, *** p ≤ 0.001).

The experiments listed in Table 5 (with level definition reported in Table 7) are performed to optimize the yield of Δ^8^ *‐iso‐*THC, obtaining the coefficients shown in bar charts in Figure 3 and in Table S5: Variable significance is strongly shifting over time. Consistent with observations for Δ⁴^(^⁸^)^‐*iso*‑THC, also the intercept $b_{0}$ decreases over time, confirming that 1 hour is the optimal reaction duration to prevent product degradation. Among linear and interaction terms, BF_3_·OEt_2_ equivalent exerts the greatest influence, while the quadratic effects are also highly significant. To locate the optimal region, we analyzed the response surface in Figure 3 [A], which indicates a plateau of yields approaching 90% for Δ⁸‑*iso*‑THC under specific CBD and catalyst conditions. To validate the model, an untested point ([0.4, ‐0.5] in coded units)—0.027 M CBD and 0.75 equiv BF_3_·OEt_2_—was evaluated. The experimentally determined confidence interval, calculated using pooled standard deviation from central replicates, overlaps with the model's predicted confidence interval (Table S3). This agreement confirms the model's robustness.

**Table 7:** Level definition of the parameters considered in the FCD for the optimization of Δ^8^‐*iso*‐THC yield in acetonitrile.

FCD for the optimization of Δ^8^‐*iso*‐THC yield			
*Variable*	*Minimum level [‐1]*	*Intermediate level [0]*	*Maximum level [1]*
*CBD concentration (M)*	0.016	0.024	0.032
*BF_3·_* ·*OEt_2_ equiv*.	0.6	0.9	1.2

## 3 Conclusion

The present paper points out the key advantages of adopting a combined experimental and chemometric strategy for the design of chemoselective and efficient synthetic protocols to THC and iso‐THC derivatives under mild conditions by strategically modulating reaction parameters.

The approach designed herein highlights a strong dependence on the nature of the solvent that mainly influences the product distribution. Targeted DoE—comprising a full factorial design (FFD) and subsequent FCDs—revealed distinct optimal conditions for each isomer, as in the following:

- **Δ⁸‐THC** is predominantly formed in both toluene and α,α,α‐trifluorotoluene.
- The yield of **Δ⁴^(^⁸^)^‐*iso*‐THC** is maximized at 0.032 M CBD and 5.1 equiv. BF_3_·OEt_2_ in acetonitrile, with a minor formation of Δ⁴‐*iso*‐THC as a secondary product.
- **Δ⁸‐*iso*‐THC** achieved an optimal yield with intermediate CBD concentrations and moderate Lewis acid concentrations as described by the response surface in Figure 3, operating in acetonitrile. The behavior was confirmed by reactions carried out on a 0.032 M concentration of CBD in the presence of 0.6 equiv. of BF_3_·OEt_2_ (see Supporting Information for further details).
- MeCN represent a peculiar case since selectivity can be smoothly directed by tuning both reaction time and amount of Lewis Acid, allowing for the isolation of four cannabinoids derivatives, including **MeCN‐Δ^4(8)^‐*iso*‐THC** deriving from incorporation of the solvent by Δ⁴^(^⁸^)^‐*iso*‐THC (see Experimental Section for further details).
- **Δ⁴^(^⁸^)^‐*iso*‐THC** and **Δ⁸‐*iso*‐THC** were consistently observed in both toluene and acetonitrile.
- **Δ⁹‐THC** was obtained in high yield using MTBE with either 1.2 or 5.1 equiv. of BF_3_·OEt_2_, along with minor amounts of alkylated ***t*Bu‐Δ⁹‐THC**

Such a strategy provided a clear, data‐driven foundation for the subsequent Design of Experiments (DoE) phase, guiding efficient optimization toward the more challenging isomeric targets. More broadly speaking, the work points out the promising advantages of integrating chemometric analysis with classical synthetic methods to streamline the discovery, optimization, and isolation of pure, standard‐grade cannabinoids. Such data‐driven strategies offer an efficient path for targeting specific cannabinoid profiles, which are increasingly relevant in pharmaceutical and analytical applications.

## 4 Experimental Section

### 4.1 Reagents and Materials

All chemicals employed in the present works were commercially available and used without any further purification unless specified. BF_3_·OEt_2_ (96%) was procured from Merck. Pharmaceutical‑grade cannabidiol (CBD, >99%) was supplied by Fagron Italia S.p.A. Anhydrous solvents (acetonitrile, toluene) and α,α,α‑trifluorotoluene (≥99%) were sourced from Merck. tert‑Butyl methyl ether (MTBE, HPLC grade, ≥99.8%) was obtained from Merck.

### 4.2 GC–MS Analysis

Analyses were performed using an Agilent 7890A GC with a 5975C single‐quadrupole mass spectrometer (Agilent Technologies, Santa Clara, CA, USA). Separation utilized a Restek HP‑5MS capillary column (30 m × 0.25 mm × 0.25 µm) with helium (>99.99%) at a constant flow of 1.0 mL·minutes^−1^. Injection volume was 1 µL in splitless mode at 250 °C. The oven was programmed from 60 °C (held for 4 minutes) to 300 °C (held for 5 minutes) at 10 °C·minutes^−1^, with data acquisition commencing 5 minutes post‐injection. The transfer line was maintained at 300 °C. Mass spectra were recorded in EI mode (70 eV) with a source temperature of 250 °C over 50–600 Da at 1460 amu·s^−1^ scan rate.

### 4.3 Peak Identification & Quantification

Peaks were assigned by matching mass spectra and retention times to an in‐house cannabinoid library^[^ ^39^, ^40^, ^41^, ^42^ ^]^ and verified using NIST08, Wiley Registry 8th Ed., SWGDRUG v3.7, and Cayman Spectral Library (2024) via ChemStation v2.1. Only peaks ≥ 5% TIC were considered unless authentic standards were available. Quantitative yields were determined by diluting quenched reaction mixtures to 100 mg·L^−1^, with 50 mg·L^−1^ olivetol as the internal standard. Calibration curves were generated using either CBD (5–100 mg·L^−1^) or pure compounds.^[^ ^43^ ^]^ A peak purity test was conducted to confirm that each peak corresponded to a single analyte.

### 4.4 Preparative HPLC

Separation was performed using a Jasco (Tokyo, Japan) HPLC system equipped with Jasco PU‐1580 pump, UV‐4070 UV/Vis detector, and HV‐2088–06 fraction valve unit. Separation was conducted on a Waters Atlantis T3 prep column (10 mm × 150 mm, 5 µm, reversed‐phase) using acetonitrile:H_2_O (60:40) at 6 mL·min^−1^. Detection was performed at 254 nm and 220 nm.

### 4.5 NMR Spectroscopy

^1^H and ^13^C NMR spectra were recorded at 300 MHz; DEPT‐135, HSQC, NOESY, and COSY were recorded at 400 MHz. Chemical shifts are reported in ppm from TMS.

### 4.6 Data Analysis

Chemometric analysis was performed using the open‐source software CAT.

### 4.7 Explorative Reactions and Multivariate Data Treatment

All explorative experiments were carried out under anhydrous conditions in oven‐dried glassware using dry, degassed solvents at room temperature. CBD served as the substrate, while boron trifluoride diethyl etherate (BF_3_·OEt_2_) was used as the Lewis acid catalyst. Reactions were initiated by adding the appropriate amount of BF_3_·OEt_2_ (1.2 or 5.1 equiv.) to 2 mL of a stirred CBD solution at defined concentrations (0.032 or 0.20 M), depending on the experimental design. Reaction progress was monitored over a 48‐hour period, with aliquots collected at 1, 3, 6, 24, and 48 hours. For gas chromatography–mass spectrometry (GC‐MS) analysis, 100 µL of the reaction mixture was quenched with 1 mL of saturated aqueous NaHCO_3_ and stirred vigorously for 20 minutes. The organic layer was then separated, diluted to a theoretical concentration of 100 mg·L^−1^, and spiked with the internal standard prior to injection.

A Design of Experiments (DoE) approach examined the effects of CBD concentration and BF_3_·OEt_2_ equiv. on product distribution across four solvents. The primary experimental matrix involved four “extreme” conditions—based on literature precedents^[^ ^18^, ^41^ ^]^—combining two CBD concentrations (0.032 and 0.20 M) with two catalyst loadings (1.2 and 5.1 eq.) in each solvent. GC‐MS yield served as the primary response variable.

For each condition, kinetic profiles were developed. The resulting multivariate dataset comprised 35 variables (kinetic profiles of seven target compounds at each time point) and 22 observations (combinations of solvent, CBD concentration, BF_3_·OEt_2_ equiv., and select replicates). After mean‐centering, the data were subjected to PCA to visualize the correlation between experimental conditions and obtained products.

Subsequently, a test set of eight reactions was conducted at an intermediate CBD concentration (0.11 M), using both catalyst loadings across all solvents to validate the model.

Based on PCA insights, a focused DoE was designed to optimize the conditions for isolating Δ^4(8)^‐*iso*‐THC and Δ^8^‐*iso*‐THC, selecting acetonitrile and toluene as solvents. A three‐factor full factorial design (FFD; 2^3^) was conducted, maintaining the previously tested CBD concentrations and catalyst equivalents, with GC‐MS yield as the response and a reaction time limit of 3 h (to avoid product degradation, as indicated by kinetic profiles and DoE‐derived b_0_ coefficients; see Supporting Information).

Since the FFD identified two contrasting optimal conditions for *iso*‐THC formation, two separate FCDs were subsequently employed to refine conditions favoring each isomer, as reported in Results and Discussion.

## 5 Preparative Experiments

**Scheme 3:**
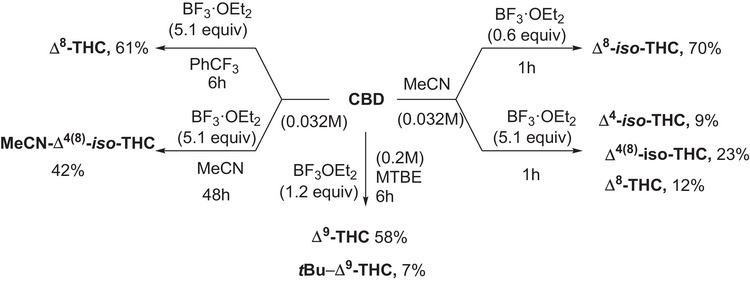
Results obtained by the preparative experiments arising from the combined DoE/experimental analysis described in the present paper.

An overview of the obtained results is available in Scheme 3.

### 5.1 Synthesis of Δ^8^‐*iso*‐THC

The conditions used for this preparative reaction have been identified using the FCD DoE dedicated to Δ^8^‐*iso*‐THC. To a magnetically stirred solution of CBD (298.6 mg, 0.95 mmol, 1.0 eq.) in dry, degassed MeCN (30 mL, 0.032 M) was added BF_3_·OEt_2_ (70 µL, 0.57 mmol, 0.6 eq.). The mixture was reacted at room temperature for 1 hour, then quenched by pouring 15 mL of a saturated NaCl and NaHCO_3_ aqueous solution to obtain a phase separation and reduce the loss of product. The mixture was stirred vigorously for 20 minutes, the two layers were separated, the aqueous phase was further extracted with Et_2_O (3×20 mL) and the combined organic layers were dried over Na_2_SO_4_. The crude residue was concentrated under reduced pressure and purified by Florisil pad (eluant: *n*‐Hex:DCM 9:1) to give **Δ^8^‐*iso*‐THC** as a yellow oil (208.4 mg, 70% yield). Spectroscopical data for **Δ^8^‐*iso*‐THC** were in accordance with the literature.

### 5.2 Synthesis of Δ^4^‐*iso*‐THC and Δ^4(8)^‐*iso*‐THC

The conditions used for this preparative reaction have been identified using the FCD DoE dedicated to Δ^4(8)^‐*iso*‐THC. To a magnetically stirred solution of CBD (299.4 mg, 0.95 mmol, 1.0 eq.) in dry, degassed MeCN (30 mL, 0.032 M) was added BF_3_·OEt_2_ (600 µL, 4.85 mmol, 5.1 eq.). The mixture was reacted at room temperature for 1 hour, then quenched by pouring 15 mL of a saturated NaCl and NaHCO_3_ aqueous solution to obtain a phase separation and reduce the loss of product. The mixture was stirred vigorously for 20 minutes, the two layers were separated, the aqueous phase extracted with Et_2_O (3×20 mL) and the combined layers were dried over Na_2_SO_4_. The crude residue was concentrated under reduced pressure and purified by silica flash column chromatography (Daily purity, cartridge size: 25, particle size: 50 µm; eluant: n‐Hex:DCM, gradient from n‐Hex to 9:2) to afford **Δ^8^‐THC** (pale yellow oil, 36.8 mg, 12% yield) and a mixture of the two compounds, which was further purified by means of preparative HPLC. The procedure afforded **Δ^4^‐*iso*‐THC** (pale yellow oil, 25.5 mg, 9% yield),^[^ ^44^ ^]^ and **Δ^4(8)^‐*iso*‐THC** (pale yellow oil, 69.8 mg, 23% yield). GC‐MS analyses of the crude point out the presence of **Δ^8^‐THC**. Spectroscopical data for **Δ^4(8)^‐*iso*‐THC** ^[^ ^45^ ^]^ and **Δ^4^‐*iso*‐THC** ^[^ ^46^ ^]^ are in accordance with the literature data.

HRMS *m/z*: [M + H]^+^ calcd for **Δ^4(8)^‐*iso*‐THC**, C_21_H_30_O_2_ 315.2319; found 315.2316

HRMS *m/z*: [M + H]^+^ calcd for **Δ^4^‐*iso*‐THC**, C_21_H_30_O_2_ 315.2319, found 315.2310

### 5.3 Synthesis of 7a‐isopropyl‐6,10‐dimethyl‐3‐pentyl‐7a,8,9,10,11,11a‐hexahydro‐1,10‐epoxydibenzo[d,f][1,3]oxazepine (MeCN‐Δ^4(8)^ *‐iso‐*THC)

To a magnetically stirred solution of CBD (300.0 mg, 0.95 mmol, 1.0 eq.) in dry, degassed MeCN (30 mL, 0.032 M) was added BF_3_·OEt_2_ (600 µL, 4.85 mmol, 5.1 eq.). The mixture was reacted at room temperature for 48 hours and quenched by pouring 5 mL of NaHCO_3_ solution (aq. sat.). The mixture is stirred vigorously for 30 minutes, then extracted with Et_2_O (3×20 mL), the combined organic layers were washed with brine (10 mL) and dried over Na_2_SO_4_. The crude residue was concentrated under reduced pressure and purified by column chromatography (Florisil 12.5 g; eluant: *n*‐Hex:Et_2_O 95:5) to give 7a‐isopropyl‐6,10‐dimethyl‐3‐pentyl‐7a,8,9,10,11,11a‐hexahydro‐1,10‐epoxydibenzo[d,f][1,3]oxazepine **(MeCN‐Δ^4(8)^‐*iso*‐THC**) as a viscous oil (142 mg, 42% isolated yield). Accordingly with the kinetic profile obtained during explorative experiments (see Figure S28), GC‐MS analyses of the crude point out the presence of **Δ^8^‐THC, Δ^4(8)^ *‐iso‐*THC,** and **Δ^8^‐*iso*‐THC**.

NMR (400 MHz, CD_3_COCD_3_) δ 6.41 (d, *J* = 1.5 Hz, 1H), 6.33 (d, *J* = 1.5 Hz, 1H), 3.66 (dt, *J* = 4.1, 1.7 Hz, 1H), 2.56–2.40 (m, 2H), 2.24 (p, *J* = 6.7 Hz, 1H), 2.14 (dd, *J* = 13.8, 2.0 Hz, 1H), 2.02 (s, 3H), 1.83–1.65 (m, 4H), 1.59 (p, *J* = 7.5 Hz, 2H), 1.40–1.26 (m, 7H), 1.19–1.08 (m, 1H), 1.01 (d, *J* = 6.5 Hz, 3H), 0.90 (m, 6H). ^13^C NMR (101 MHz, CDCl_3_) δ 158.0, 156.3, 149.7, 143.6, 116.7, 112.2, 110.6, 74.8, 63.2, 36.7, 36.3, 35.8, 32.9, 32.6, 32.1, 30.7, 30.5, 29.3, 24.8, 23.5, 18.0, 17.3, 14.7. HRMS *m/z*: [M + H]^+^ calcd for C_23_H_37_O_2_N 356.2584; found 356.2581.

### 5.4 Synthesis of Δ^8^‐THC

To a magnetically stirred solution of CBD (299.4 mg, 0.95 mmol, 1.0 eq.) in dry, degassed α, α, α – trifluorotoluene (30 mL, 0.032 M) was added BF_3_·OEt_2_ (600 µL, 4.85 mmol, 5.1 eq.). The mixture was reacted at room temperature for 6 hours and quenched by pouring 15 mL of NaHCO_3_ solution (aq. sat.) and stirring vigorously for 20 minutes. The two layers were separated, the aqueous phase further extracted with Et_2_O (3×20 mL), the combined organic layers were washed with brine (10 mL) and dried over Na_2_SO_4_. The crude residue was concentrated under reduced pressure and purified by column chromatography (Florisil 12.5 g; eluant: *n*‐Hex:Et_2_O 95:5) to afford **Δ^8^‐THC** (pale yellow oil, 182 mg, 61% yield). Spectroscopical data for **Δ** ^**8**^ **‐THC** are in accordance with the literature data.^[^ ^45^ ^]^

### 5.5 Synthesis of Δ^9^‐THC and *t*Bu‐Δ**^9^**‐THC

To a magnetically stirred solution of CBD (301.9 mg, 0.96 mmol, 1.0 eq.) in dry, degassed MTBE (5 mL, 0.20 M) was added BF_3_·OEt_2_ (140 µL, 1.1 mmol, 1.2 eq.). The mixture was reacted at room temperature for 6 hours and quenched by pouring 3 mL of NaHCO_3_ solution (aq. sat.) and stirring vigorously for 20 minutes. The two layers were separated, the aqueous phase extracted with Et_2_O (3×20 mL), and the combined layers were washed with brine (10 mL) and dried over Na_2_SO_4_. The crude residue was concentrated under reduced pressure and purified by silica gel column chromatography (15.0 g; eluant: *n*‐Hex:DCM with 0.5% Et_3_N to neutralize the acidity of the silica, gradient from 95:5 to 85:15) to afford **Δ^9^‐THC** (175.7 mg, 58% yield) as a yellow oil, and ***t*Bu‐Δ^9^‐THC** (25.0 mg, 7% yield) as a yellow oil. Spectroscopical data for **Δ^9^‐THC** are in accordance with the literature data.^[^ ^45^ ^]^

^1^H NMR (400 MHz, Acetone) δ 6.50 (t, *J* = 1.7 Hz, 1H), 6.44 (d, *J* = 1.7 Hz, 1H), 6.30 (d, *J* = 1.8 Hz, 1H), 3.28–3.15 (m, 1H), 2.51–2.43 (m, 2H), 2.17–2.11 (m, 2H), 1.971.88 (m, 2H), 1.64 (dd, *J* = 2.4, 1.3 Hz, 3H), 1.60–1.51 (m, 4H), 1.43 (s, 1H), 1.36 (s, 9H), 1.33–1.28 (m, 8H), 1.05 (s, 3H), 0.90–0.85 (m, 3H). ^13^C NMR (101 MHz, Acetone) δ 156.6, 155.8, 142.7, 133.7, 126. 6, 117.2, 115.9, 113.2, 80.4, 77.5, 47.3, 36.5, 36.1, 32.5, 32.1, 32.1, 28.2, 26.2, 23.9, 23.5, 19.8, 14.7. HRMS *m/z*: [M + H]^+^ calcd for C_25_H_39_O_2_ 371.2945, found 371.2940

## Supporting Information

Chemometrics analyses, copy of the ^1^H and ^13^C NMR spectra for the isolated compounds; GC‐MS and HRMS analyses.

## Conflict of Interest

The authors declare no conflict of interest.

## Supporting information



Supporting Information

## Data Availability

The data that support the findings of this study are available in the supplementary material of this article.
