# Supplementary material for: Chemometric Optimization of BF3·OEt2‐Mediated Cyclization of Cannabidiol to Rare Δ⁴‐ and Iso‐THC Isomers
Source: Chemistry. 2025 Oct 30;31(66):e02387. doi: 10.1002/chem.202502387 (PMC12648456; doi:10.1002/chem.202502387)
Supplement: Supplementary file 1 — Supporting Information [file CHEM-31-e02387-s001.pdf]

Chemometric Optimization of $BF_3 \cdot OEt_2$-Mediated Cyclization of Cannabidiol to Rare Δ^4- and *iso*-THC Isomers

Arianna Bini,[a] Lisa Rita Magnaghi, [a] Valeria Cavalloro, [b] Alessandra Bonanni,[a] Stefano Protti,[a]* Daniele Merli[a]*

[a] Department of Chemistry, University of Pavia, Viale Taramelli 10, 27100 Pavia, Italy

[b] Department of Earth and Environmental Sciences, Via A. Ferrata 7, 27100 Pavia, Italy

Table Of Contents

1. **Mass fragmentation of reported compounds** **S2**
2. **Explorative reactions of CBD in the solvents considered** **S10**
3. **Design of Experiments (DoE)** **S26**
4. **^{1}H and ^{13}C NMR Spectra for the isolated compounds** **S30**

1. Mass fragmentation of reported compounds

1.1. CBD

The ten major peaks in the mass fragmentation spectra of this compound are:

m/z	*231*	*68*	*67*	*55*	*174*	*53*	*232*	*91*	*175*	*77*
Relative abundance	*999*	*541*	*523*	*217*	*199*	*162*	*149*	*120*	*89*	*83*

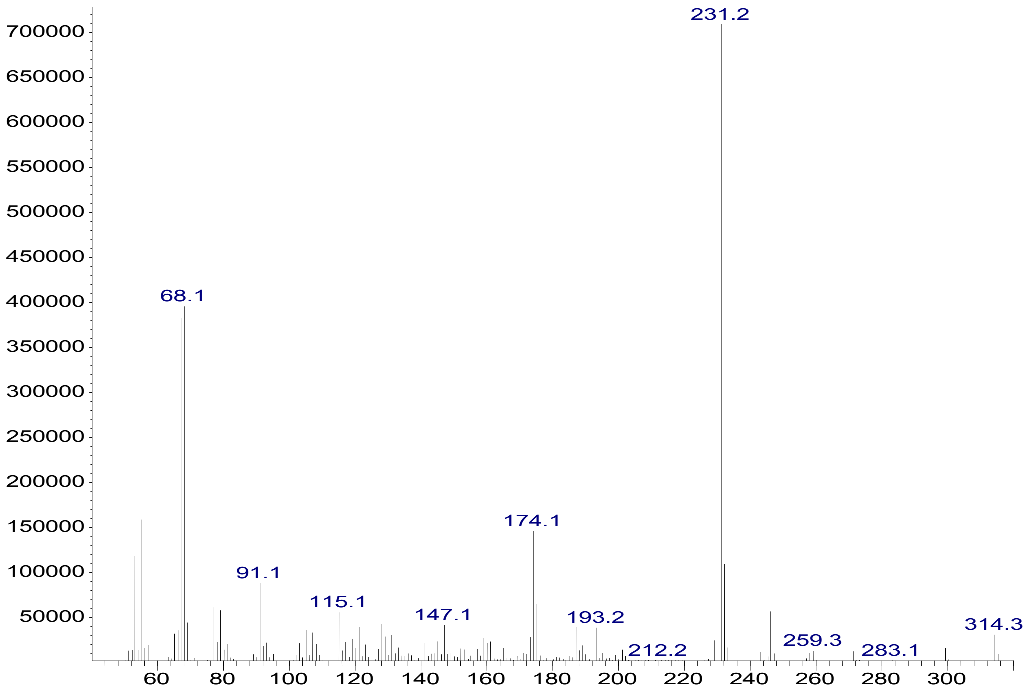

Fig. S1 *Mass fragmentation of CBD.*

1.2. Δ⁴-*iso*-THC

The ten major peaks in the mass fragmentation spectra of this compound are:

m/z	*271*	*314*	*55*	*231*	*67*	*201*	*91*	*272*	*193*	*174*
Relative abundance	*999*	*381*	*304*	*303*	*242*	*242*	*197*	*194*	*171*	*163*

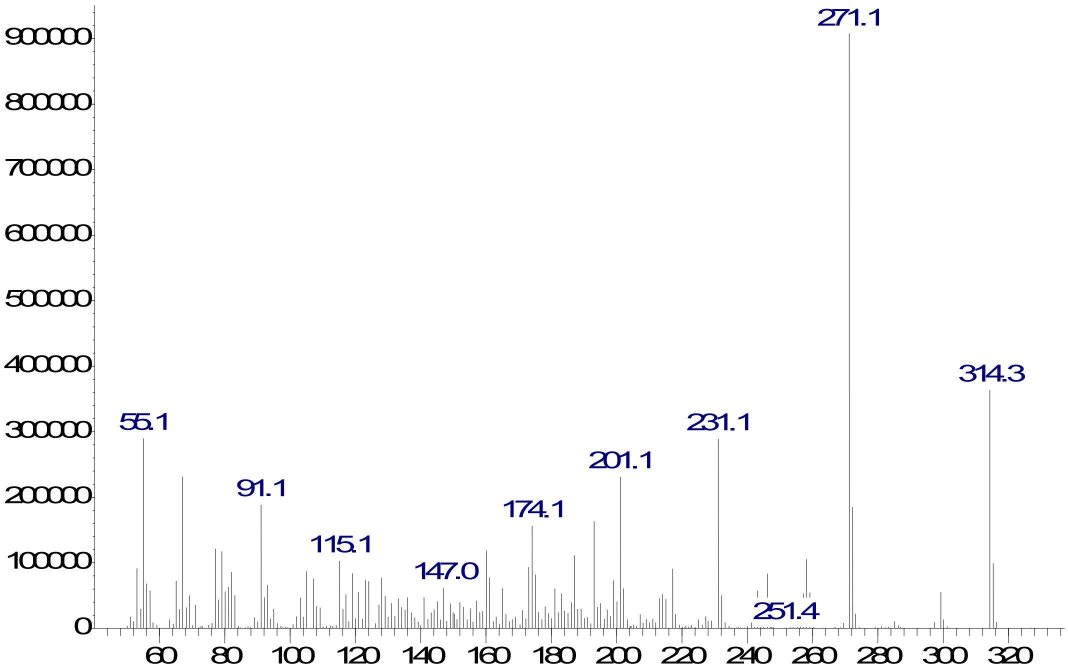

Fig. S2 *Mass fragmentation of Δ⁴-iso-THC.*

1.3. $\Delta^{4(8)}$-*iso*-THC

The ten major peaks in the mass fragmentation spectra of this compound are:

m/z	*271*	*314*	*231*	*55*	*258*	*299*	*91*	*67*	*187*	*174*
Relative abundance	*999*	*845*	*820*	*702*	*451*	*408*	*395*	*369*	*311*	*304*

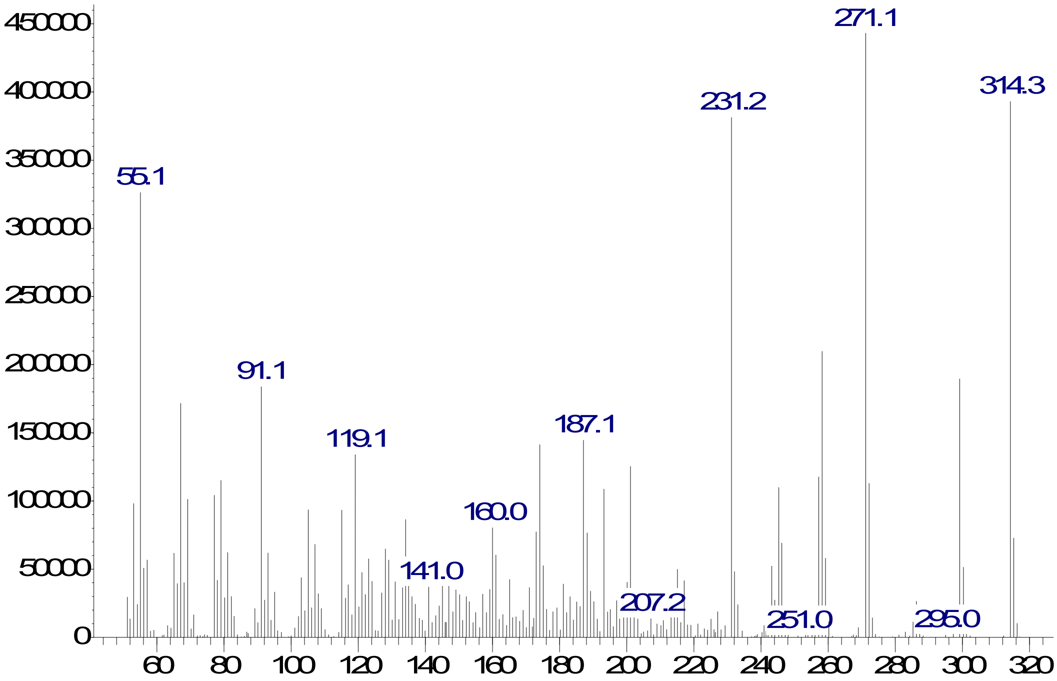

Fig. S3 *Mass fragmentation of $\Delta^{4(8)}$-iso-THC.*

1.4. Δ⁸-*iso*-THC

The ten major peaks in the mass fragmentation spectra of this compound are:

m/z	*231*	*67*	*55*	*174*	*232*	*314*	*68*	*81*	*233*	*91*
Relative abundance	*999*	*216*	*195*	*165*	*152*	*118*	*110*	*95*	*84*	*79*

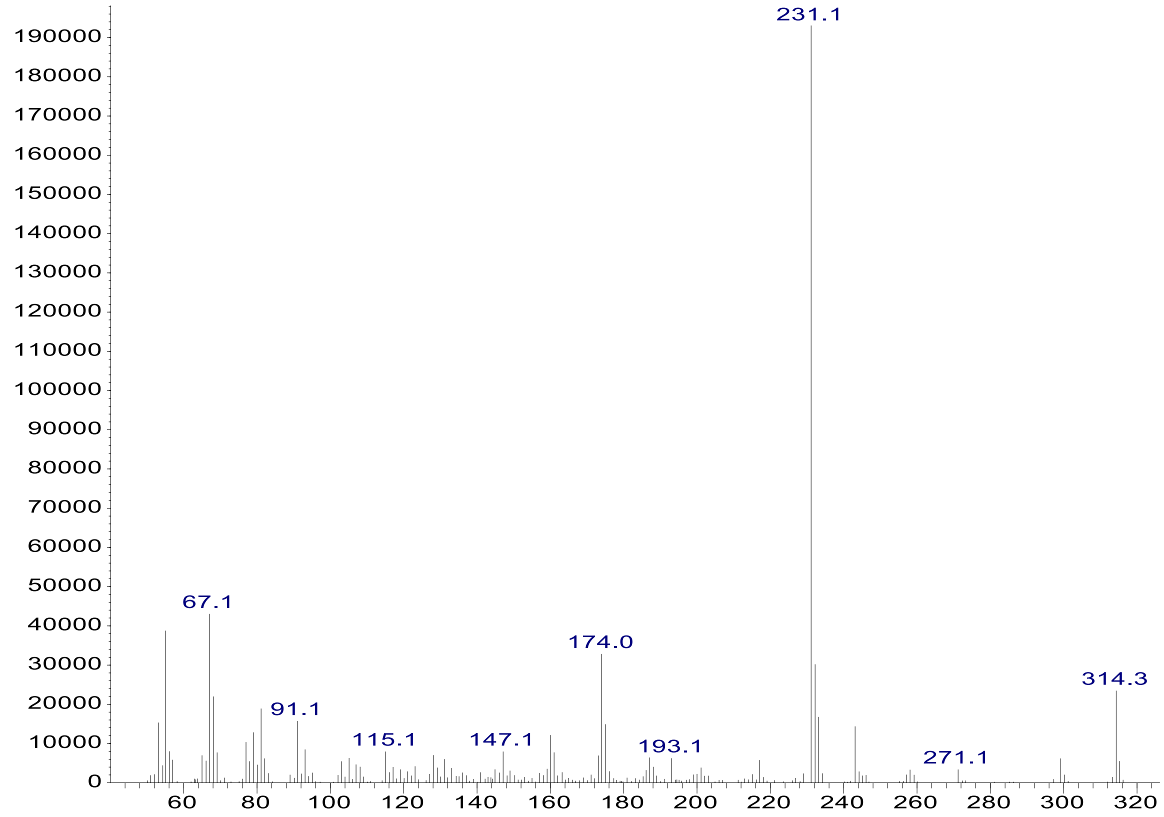

Fig. S4 *Mass fragmentation of Δ⁸-iso-THC.*

1.5. *t*Bu-Δ⁹-THC

The ten major peaks in the mass fragmentation spectra of this compound are:

m/z	*57*	*299*	*314*	*231*	*271*	*243*	*258*	*315*	*300*	*55*
Relative abundance	*999*	*397*	*382*	*199*	*160*	*104*	*103*	*89*	*77*	*76*

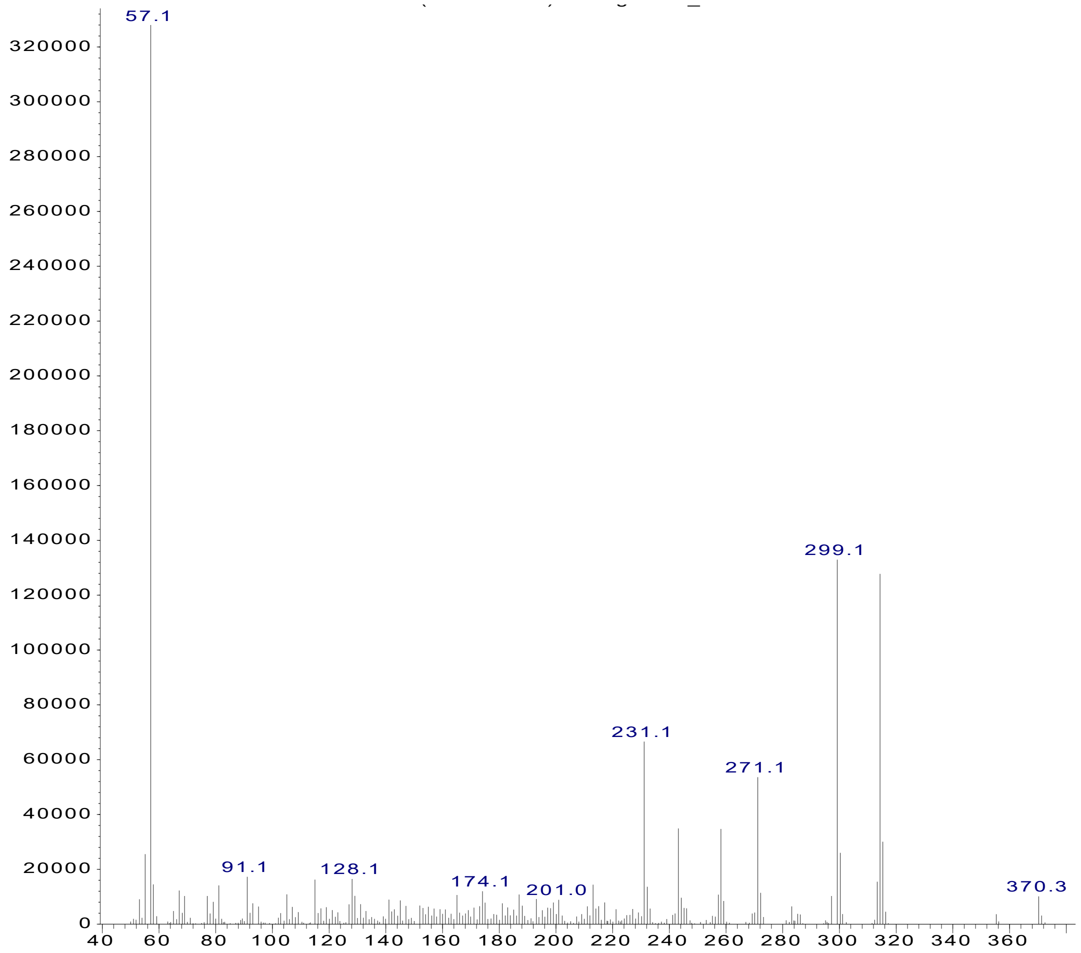

Fig. S5 *Mass fragmentation of tBu-Δ⁹-THC.*

1.6. MeCN-Δ$^{4(8)}$-*iso*-THC

The ten major peaks in the mass fragmentation spectra of this compound are:

m/z	*231*	*314*	*271*	*232*	*174*	*55*	*258*	*299*	*69*	*91*
Relative abundance	*999*	*362*	*333*	*216*	*193*	*192*	*178*	*173*	*159*	*156*

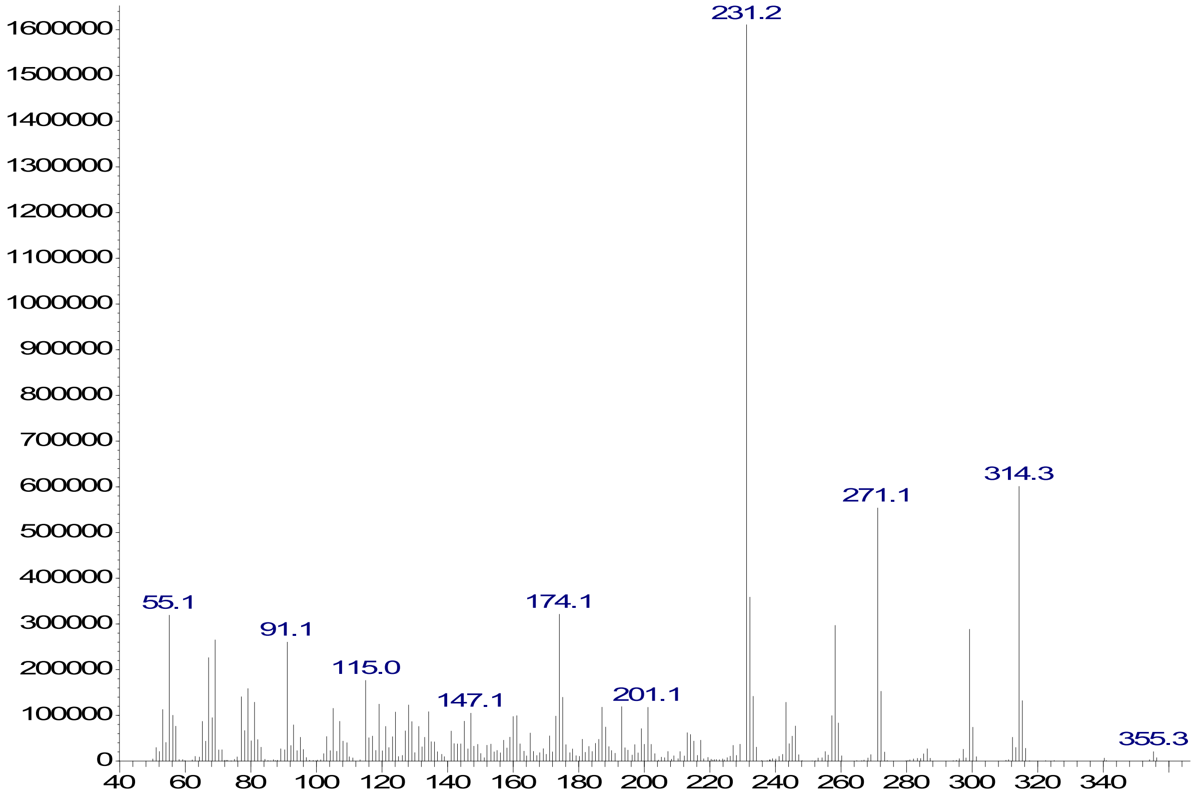

Fig. S6 *Mass fragmentation of MeCN-Δ$^{4(8)}$-iso-THC.*

1.7. Δ⁸-THC

The ten major peaks in the mass fragmentation spectra of this compound are:

m/z	*231*	*314*	*67*	*258*	*68*	*55*	*271*	*174*	*91*	*53*
Relative abundance	*999*	*437*	*384*	*274*	*259*	*246*	*227*	*226*	*216*	*161*

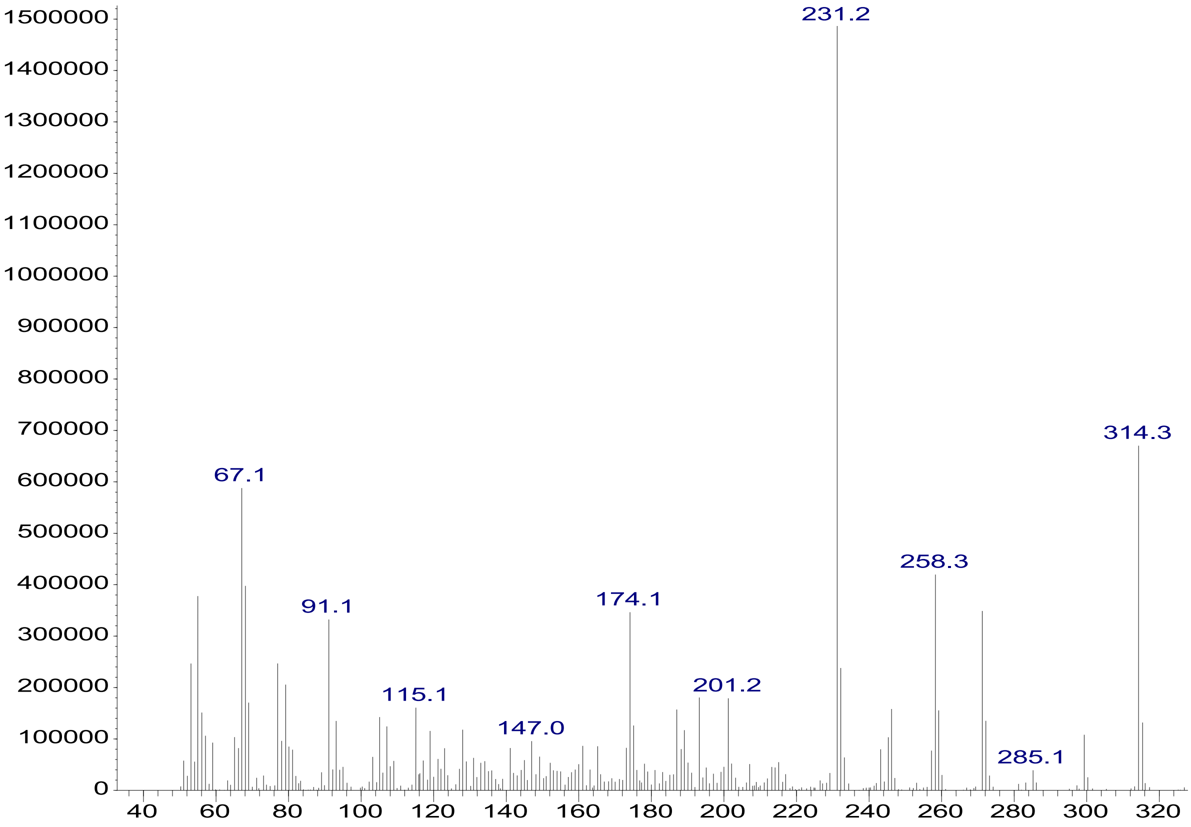

Fig. S7 *Mass fragmentation of MeCN-$\Delta^{4(8)}$-iso-THC.*

1.8. Δ⁹-THC

The ten major peaks in the mass fragmentation spectra of this compound are:

m/z	*299*	*314*	*231*	*271*	*55*	*91*	*243*	*67*	*77*	*300*
Relative abundance	*999*	*826*	*684*	*467*	*415*	*339*	*338*	*250*	*233*	*223*

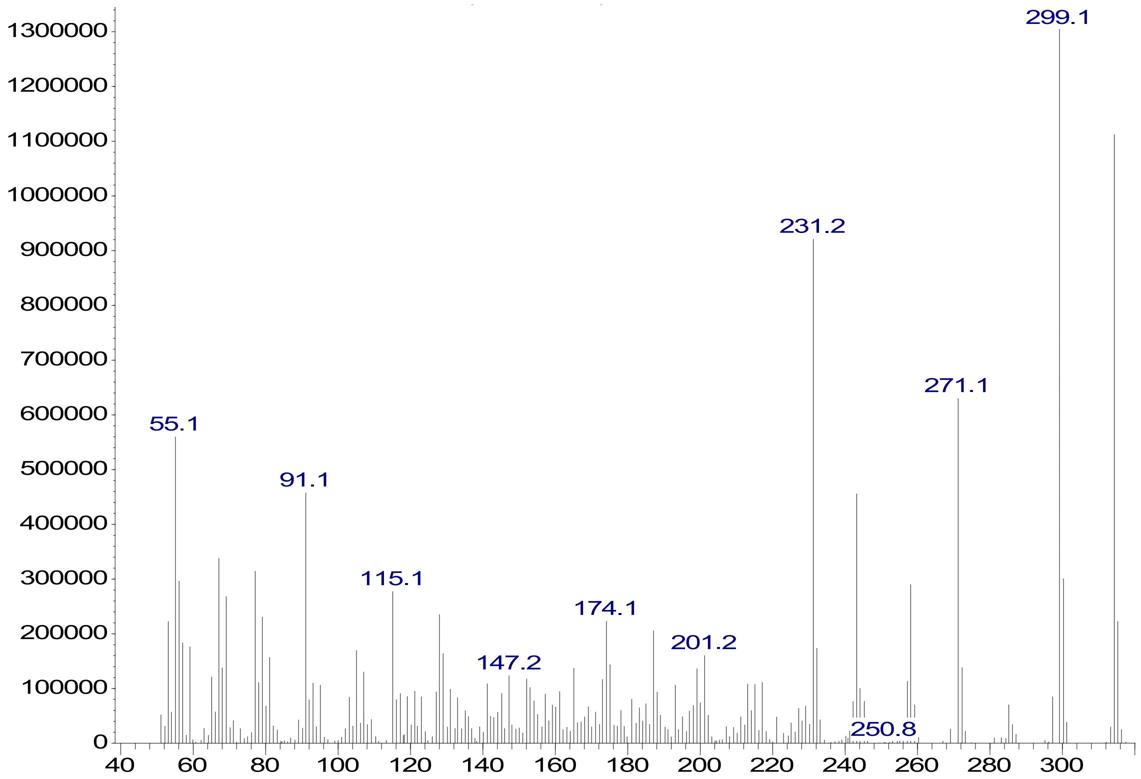

Fig. S8 *Mass fragmentation of Δ⁹-THC.*

2. Explorative reactions of CBD in the solvents considered

2.1. Toluene

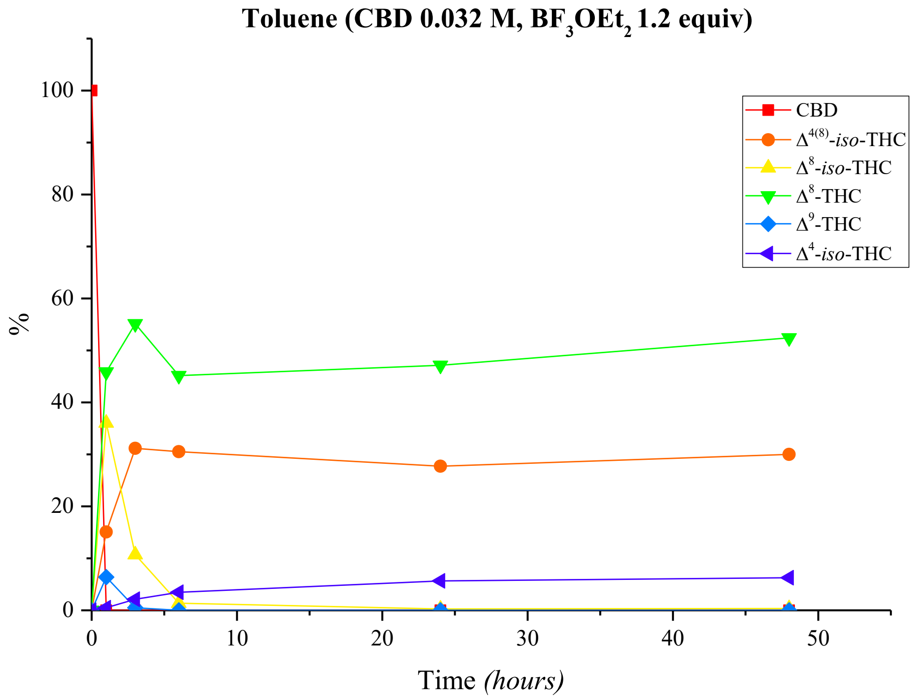

Fig. S9 *Time-dependent consumption of CBD and product formation. Conditions: CBD 0.032 M, BF_3OEt_2 1.2 equivalents.*

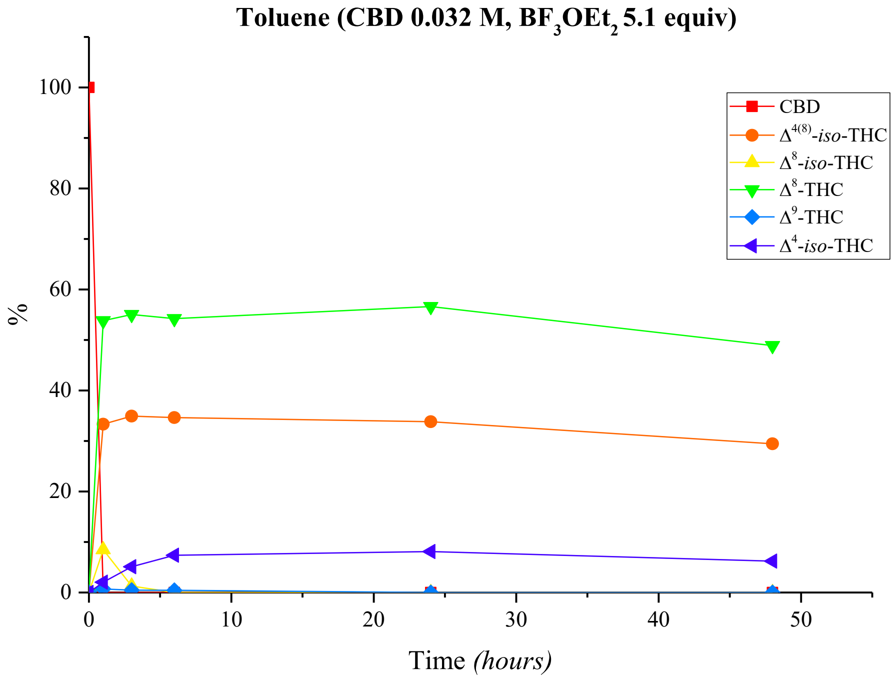

Fig. S10 *Time-dependent consumption of CBD and product formation in toluene. Conditions: CBD 0.032 M, BF_3OEt_2 5.1 equivalents.*

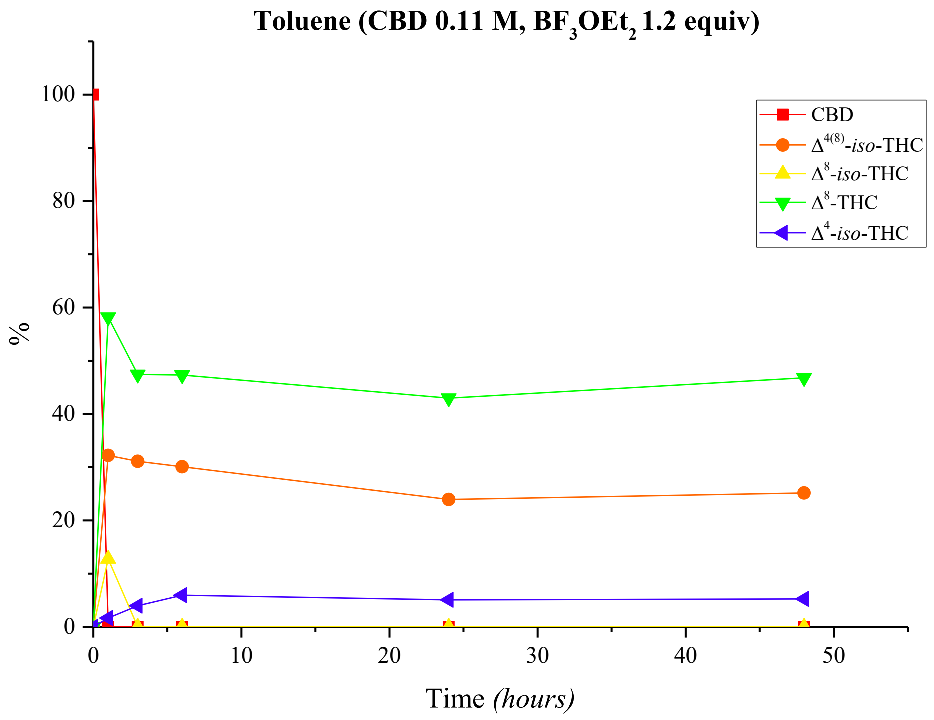

Fig. S11 *Time-dependent consumption of CBD and product formation in toluene. Conditions: CBD 0.11 M, BF_3OEt_2 1.2 equivalents.*

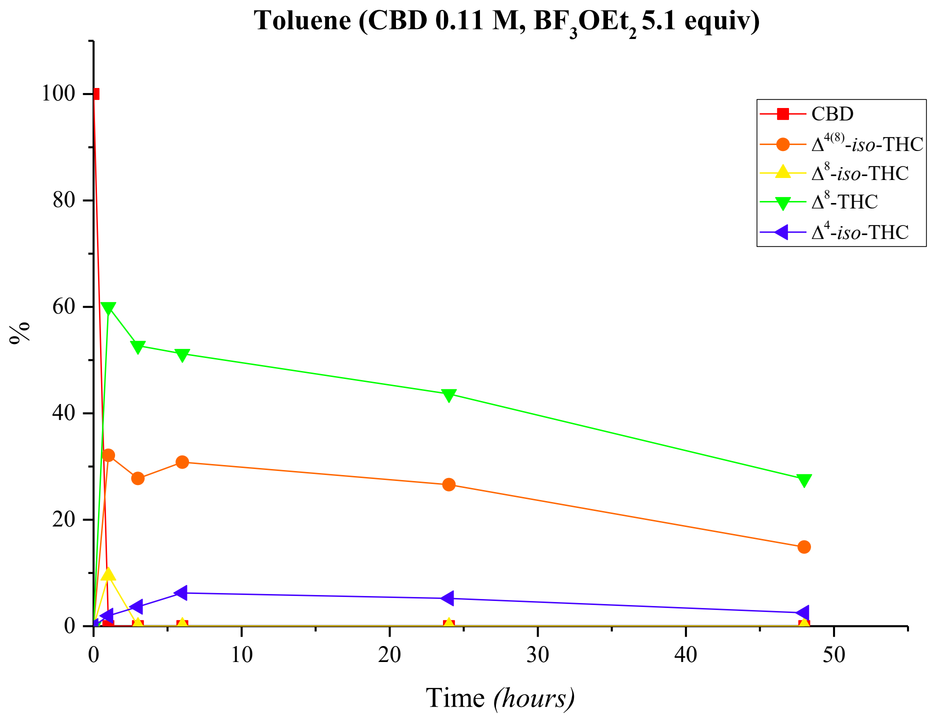

Fig. S12 *Time-dependent consumption of CBD and product formation in toluene. Conditions: CBD 0.11 M, BF_3OEt_2 5.1 equivalents.*

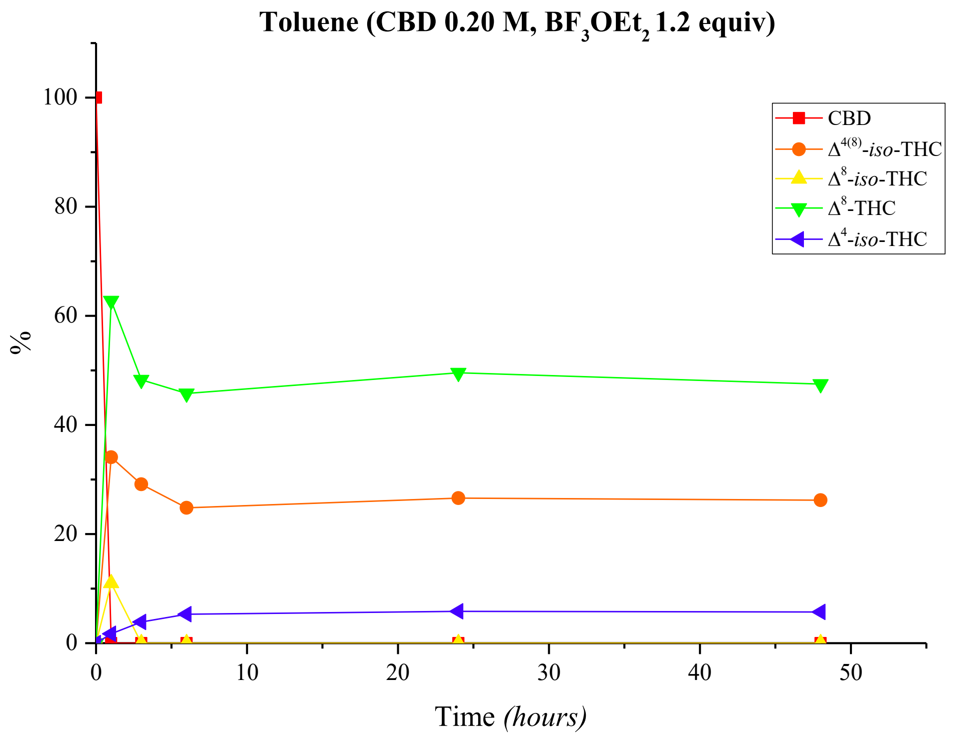

***Fig. S13** Time-dependent consumption of CBD and product formation in toluene. Conditions: CBD 0.20 M, BF_3OEt_2 1.2 equivalents.*

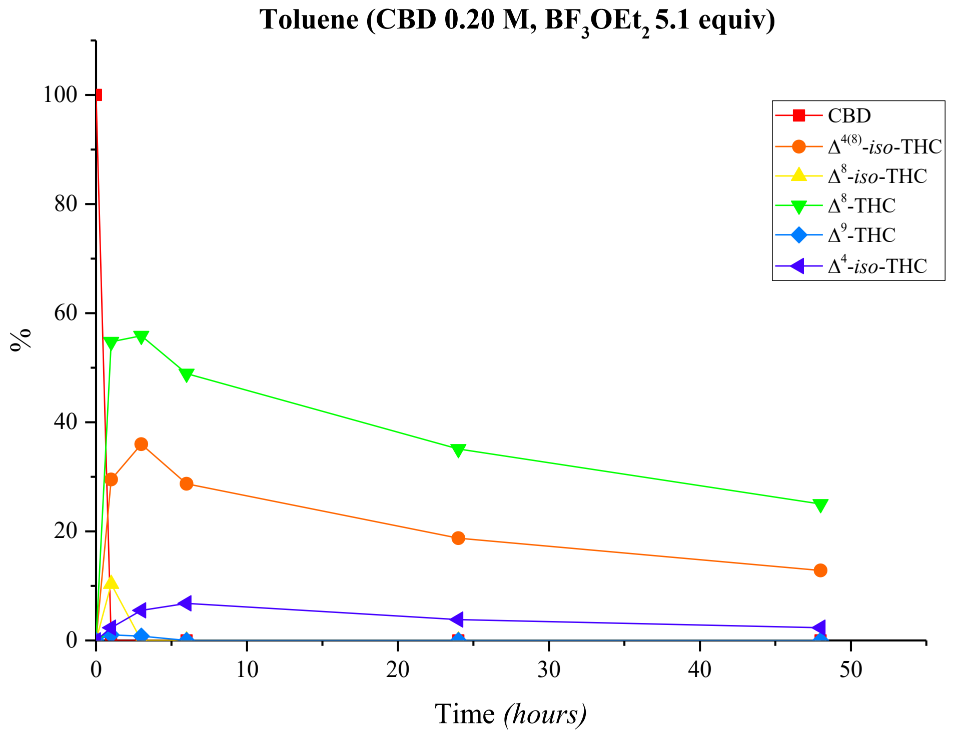

***Fig. S14** Time-dependent consumption of CBD and product formation in toluene. Conditions: CBD 0.20 M, BF_3OEt_2 5.1 equivalents.*

As apparent from Figures S9-S14, CBD in Toluene is efficiently converted to Δ^8-THC (main product, that has been detected in up to 63% yield, when a 0.20 M solution of CBD has been treated with 1.2 equiv. of BF_3OEt_2, see Figure S13) and $\Delta^{4(8)}$-*iso*-THC. Δ^4-*iso*-THC was also observed as secondary product, derived from the acid catalyzed isomerization of $\Delta^{4(8)}$-*iso*-THC.

2.2. α, α, α-trifluorotoluene

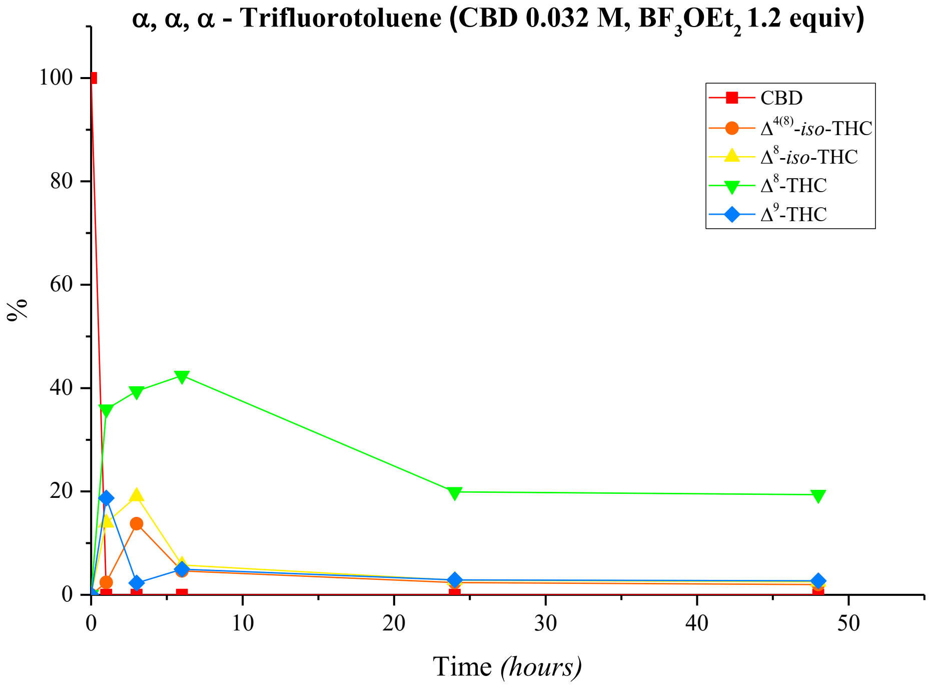

Fig. S15 *Time-dependent consumption of CBD and product formation in α, α, α - trifluorotoluene. Conditions: CBD 0.032 M, BF_3OEt_2 1.2 equivalents.*

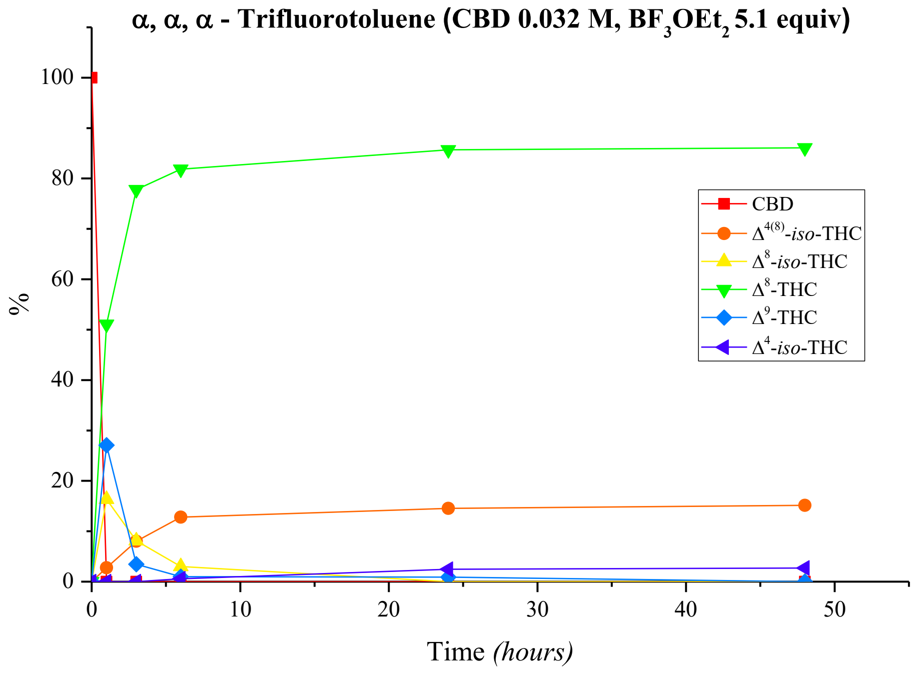

Fig. S16 *Time-dependent consumption of CBD and product formation in α, α, α - trifluorotoluene. Conditions: CBD 0.032 M, BF_3OEt_2 5.1 equivalents.*

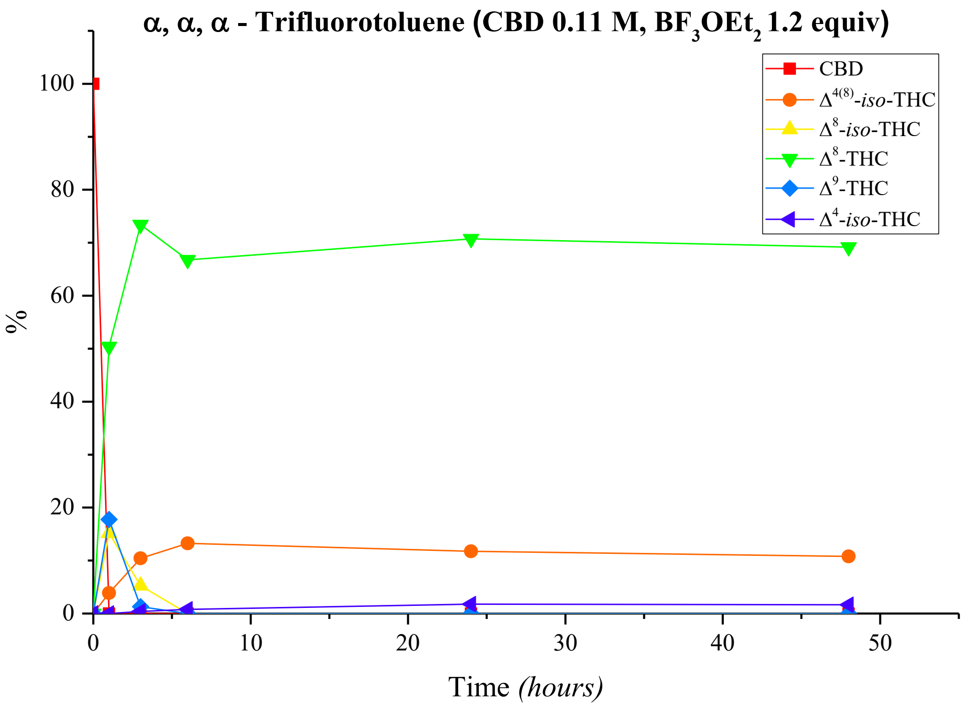

Fig. S17 *Time-dependent consumption of CBD and product formation in α, α, α - trifluorotoluene. Conditions: CBD 0.11 M, BF_3OEt_2 1.2 equivalents.*

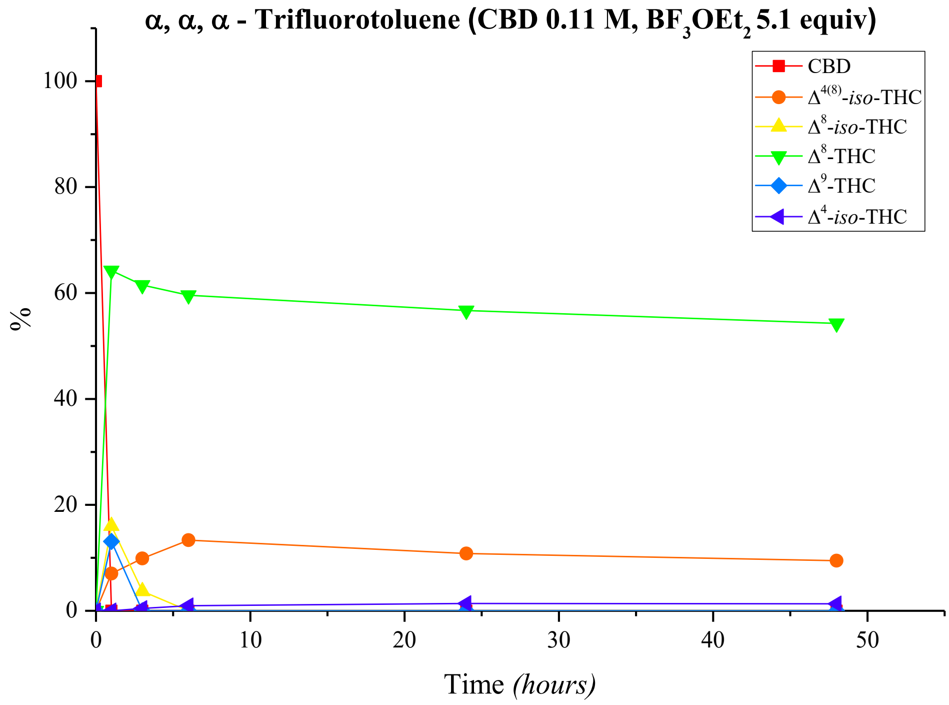

Fig. S18 *Time-dependent consumption of CBD and product formation in α, α, α - trifluorotoluene. Conditions: CBD 0.11 M, BF_3OEt_2 5.1 equivalents.*

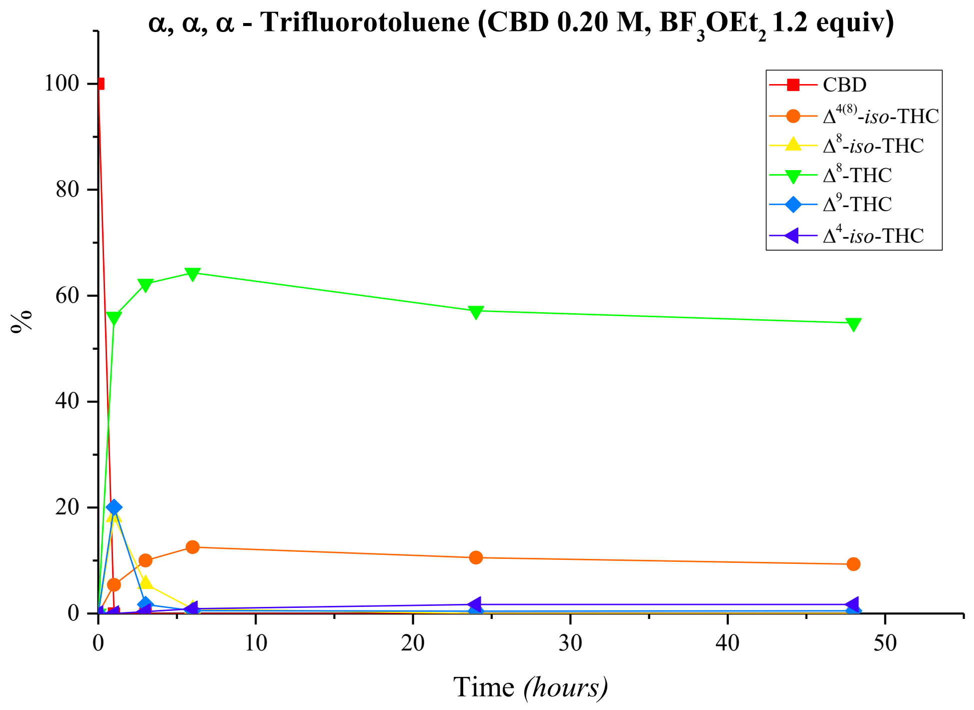

Fig. S19 *Time-dependent consumption of CBD and product formation in α, α, α - trifluorotoluene. Conditions: CBD 0.20 M, BF_3OEt_2 1.2 equivalents.*

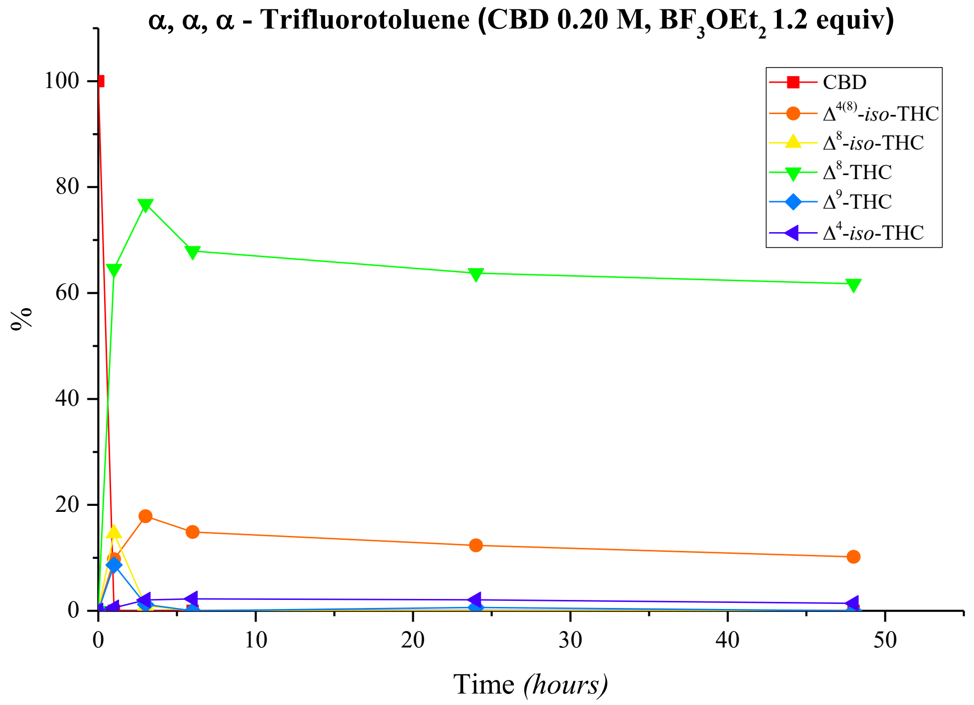

Fig. S20 *Time-dependent consumption of CBD and product formation in α, α, α - trifluorotoluene. Conditions: CBD 0.20 M, BF_3OEt_2 5.1 equivalents.*

As depicted in Figures S15-S20, Δ^8-THC is the main product obtained from the treatment of CBD with BF_3OEt_2, in α,α,α–Trifluorotoluene, along with a minor amount of $\Delta^{4(8)}$-*iso*-THC. Low concentrations of Δ^9-THC were observed at short reaction times (< 5 h). The conversion process achieves an almost quantitative efficiency under the conditions described in Fig. S16 (a 0.032 M solution of CBD treated with 5.1 equiv. of BF_3OEt_2).

2.3. MTBE

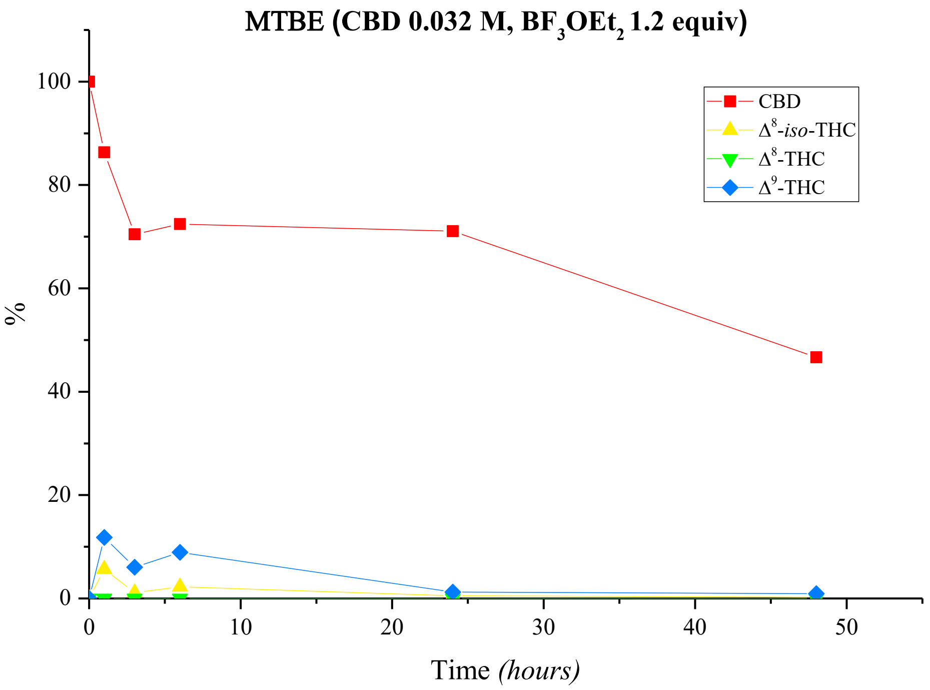

Fig. S21 *Time-dependent consumption of CBD and product formation in MTBE. Conditions: CBD 0.032 M, BF_3OEt_2 1.2 equivalents.*

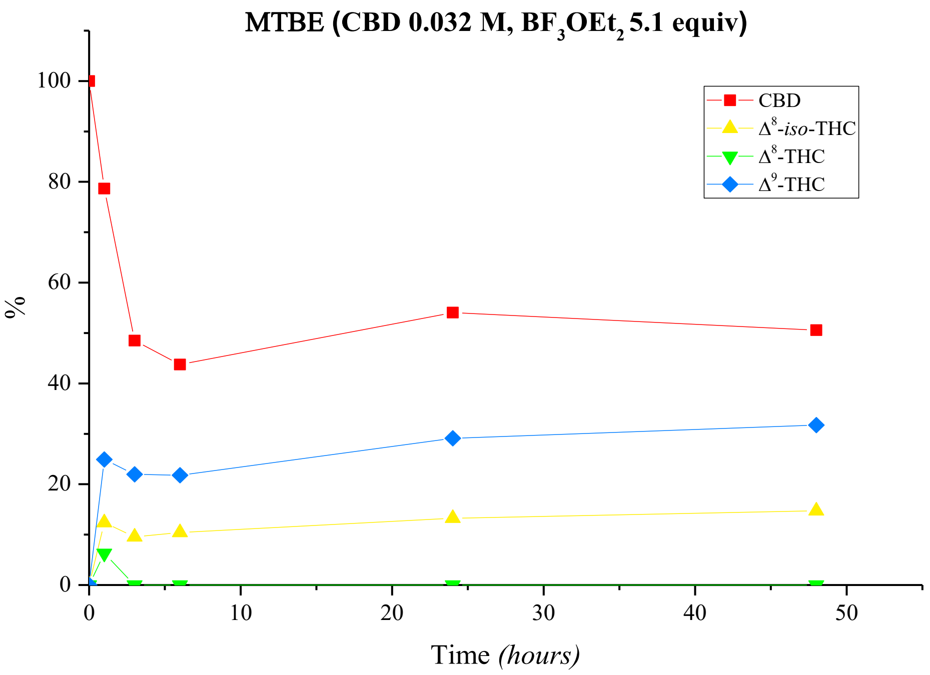

Fig. S22 *Time-dependent consumption of CBD and product formation in MTBE. Conditions: CBD 0.032 M, BF_3OEt_2 5.1 equivalents.*

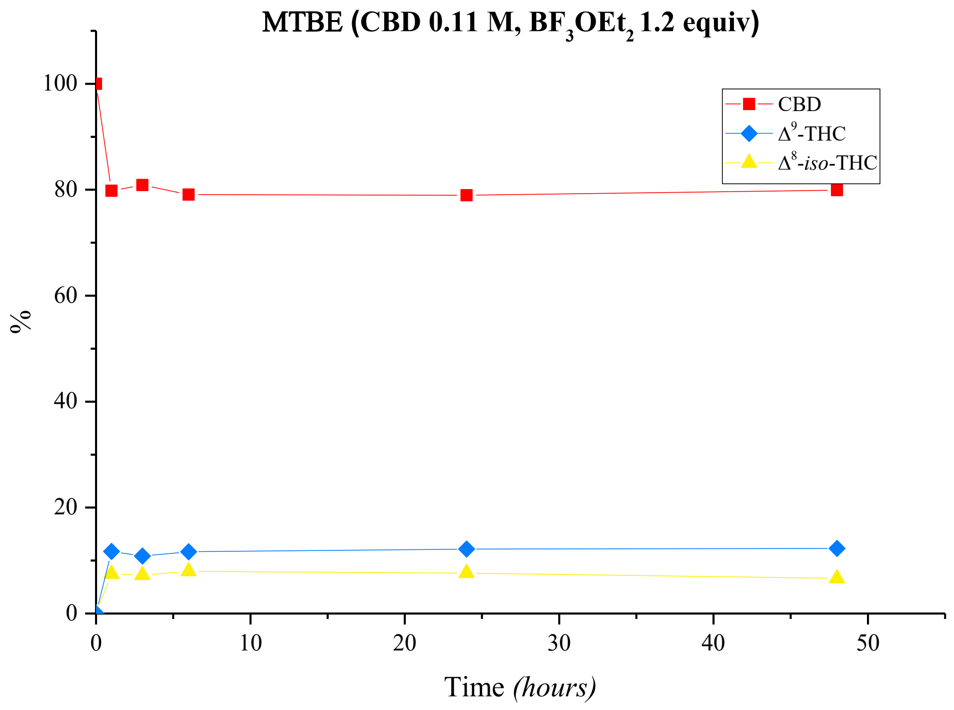

Fig. S23 *Time-dependent consumption of CBD and product formation in MTBE. Conditions: CBD 0.11 M, BF_3OEt_2 1.2 equivalents.*

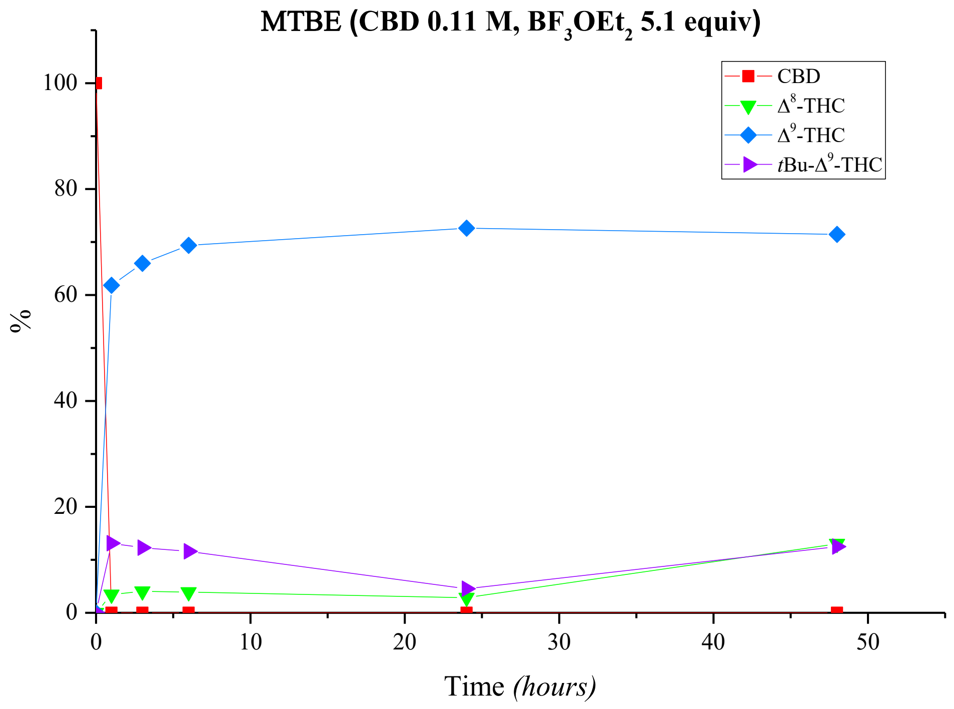

Fig. S24 *Time-dependent consumption of CBD and product formation in MTBE. Conditions: CBD 0.11 M, BF_3OEt_2 5.1 equivalents.*

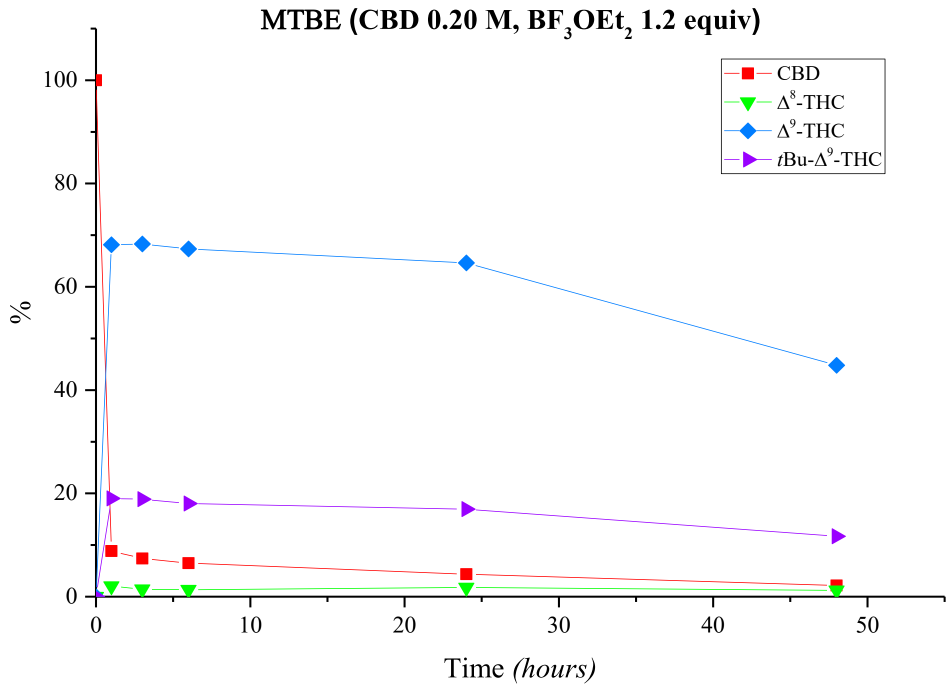

Fig. S25 *Time-dependent consumption of CBD and product formation in MTBE. Conditions: CBD 0.20 M,* BF_3OEt_2 *1.2 equivalents.*

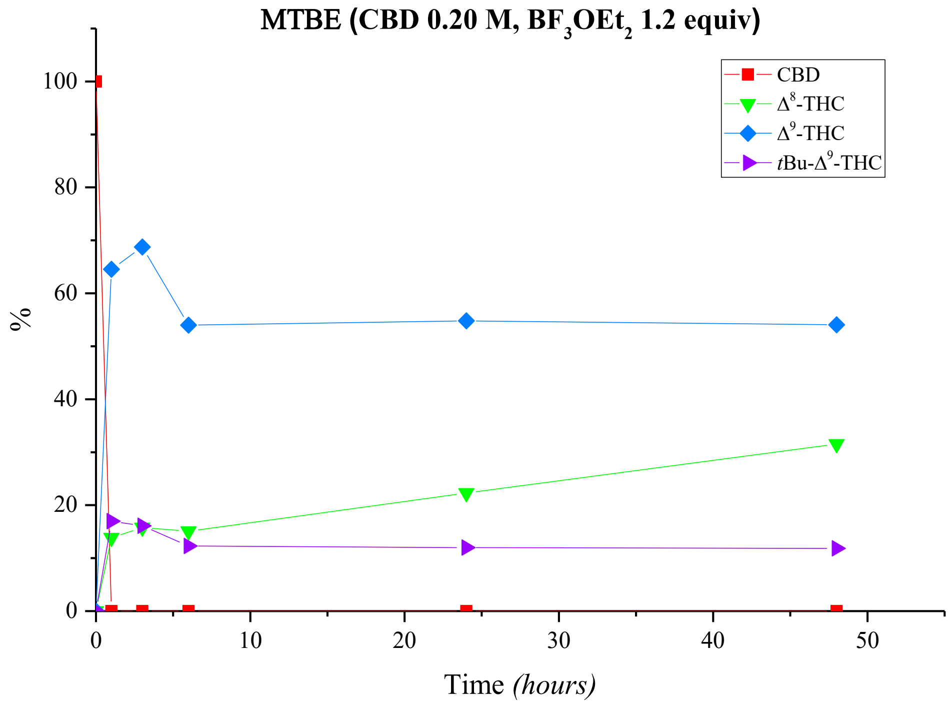

Fig. S26 *Time-dependent consumption of CBD and product formation in MTBE. Conditions: CBD 0.20 M,* BF_3OEt_2 *5.1 equivalents.*

As depicted in Figures S21-S26, Δ^9-THC is the main product obtained from the treatment of CBD at high concentration with BF_3OEt_2 along with minor amounts of tBu-Δ^9-THC. When the same process was carried out on diluted solutions of CBD, no significant conversion of the starting substrate was observed.

2.4. MeCN

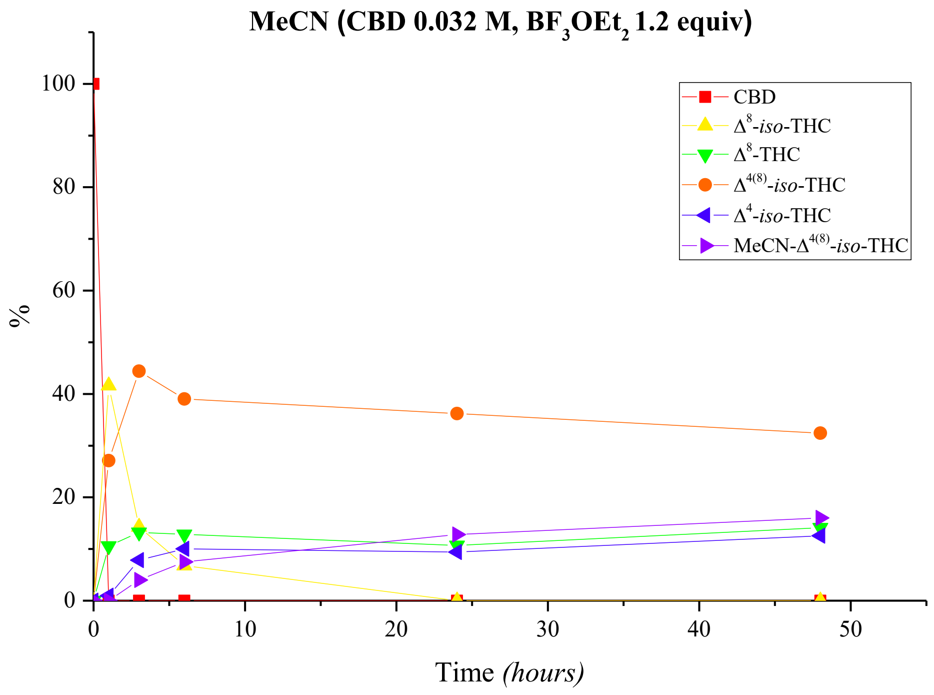

Fig. S27 *Time-dependent consumption of CBD and product formation in acetonitrile. Conditions: CBD 0.032 M, BF_3OEt_2 1.2 equivalents.*

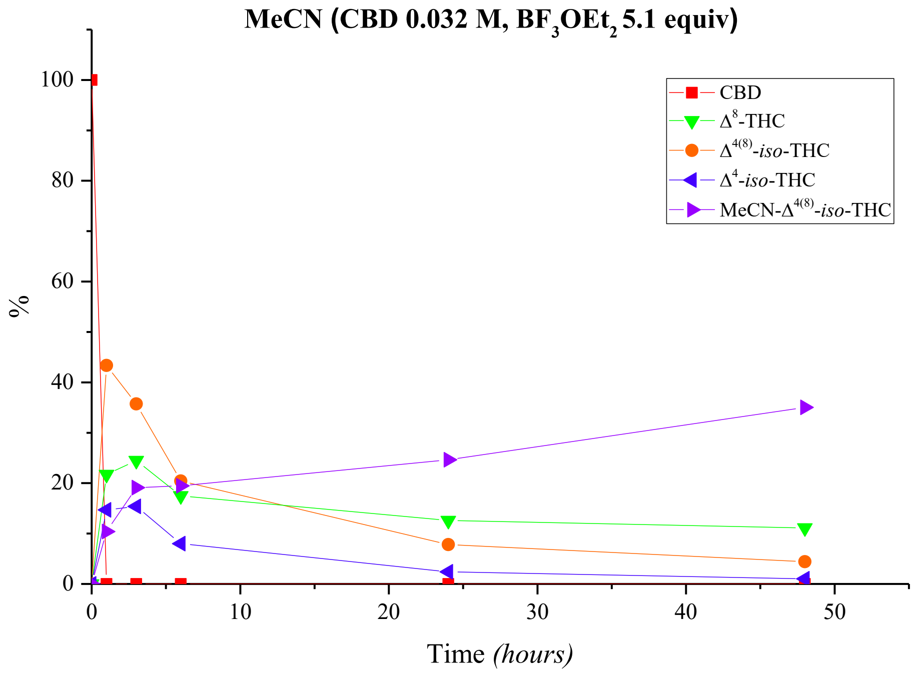

Fig. S28 *Time-dependent consumption of CBD and product formation in acetonitrile. Conditions: CBD 0.032 M, BF_3OEt_2 5.1 equivalents.*

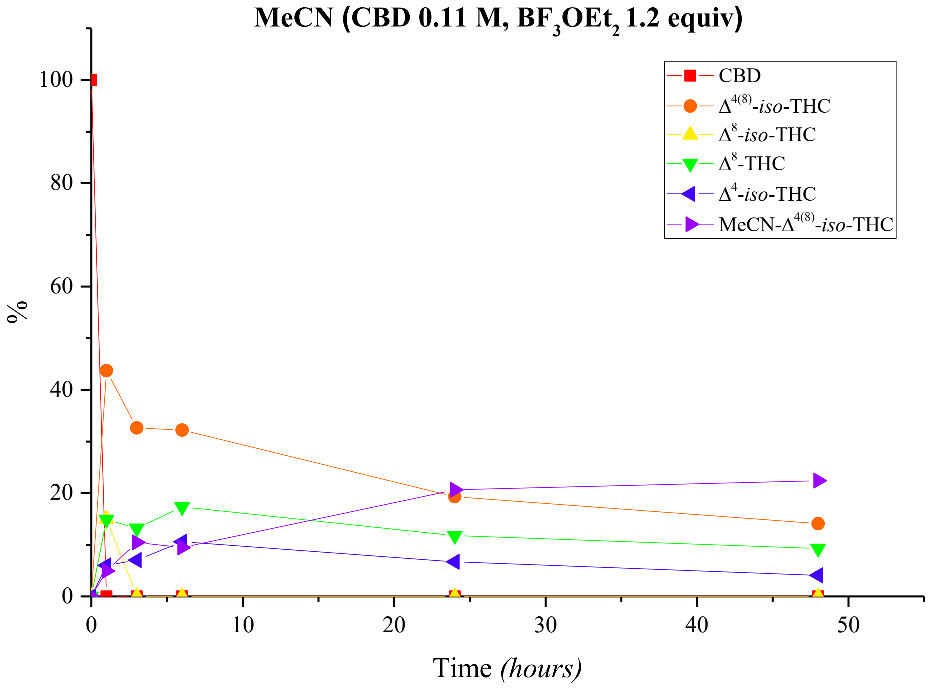

Fig. S29 *Time-dependent consumption of CBD and product formation in acetonitrile. Conditions: CBD 0.11 M, BF$_3$OEt$_2$ 1.2 equivalents.*

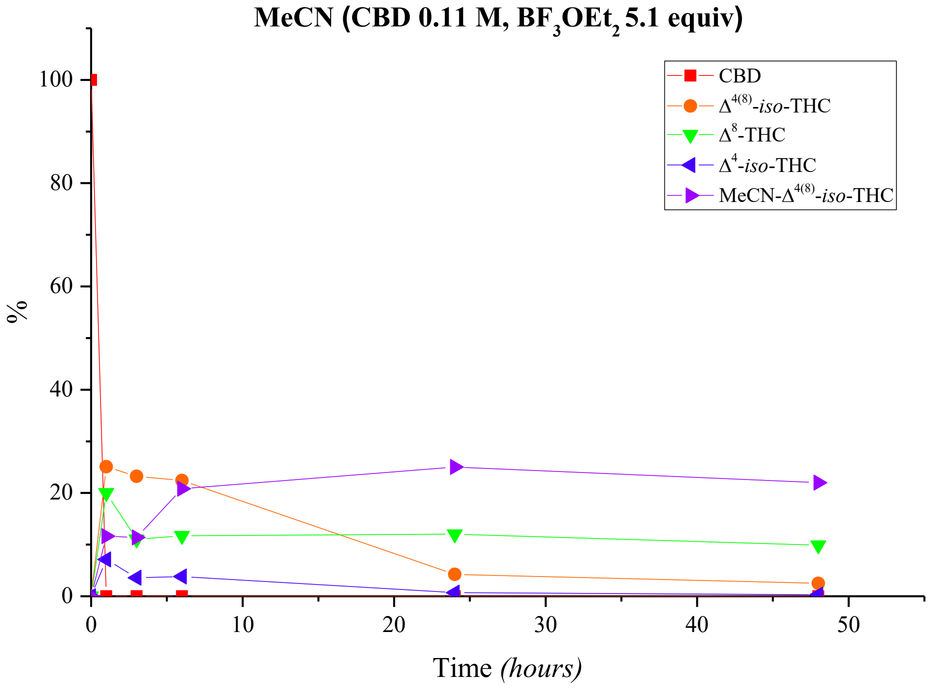

Fig. S30 *Time-dependent consumption of CBD and product formation in acetonitrile. Conditions: CBD 0.11 M, BF$_3$OEt$_2$ 5.1 equivalents.*

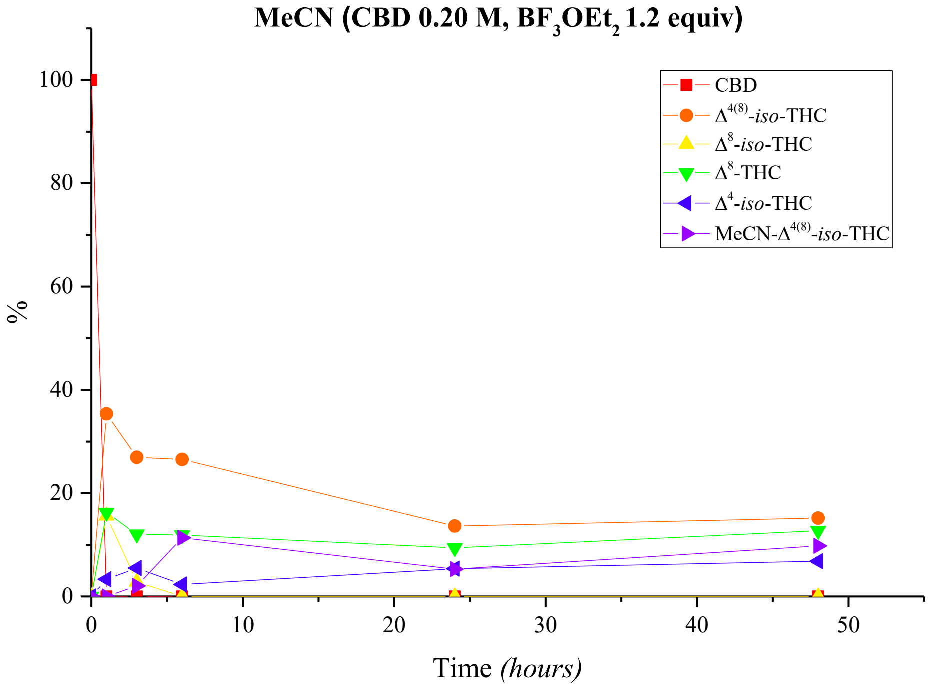

Fig. S31 *Time-dependent consumption of CBD and product formation in acetonitrile. Conditions: CBD 0.20 M, BF_3OEt_2 1.2 equivalents.*

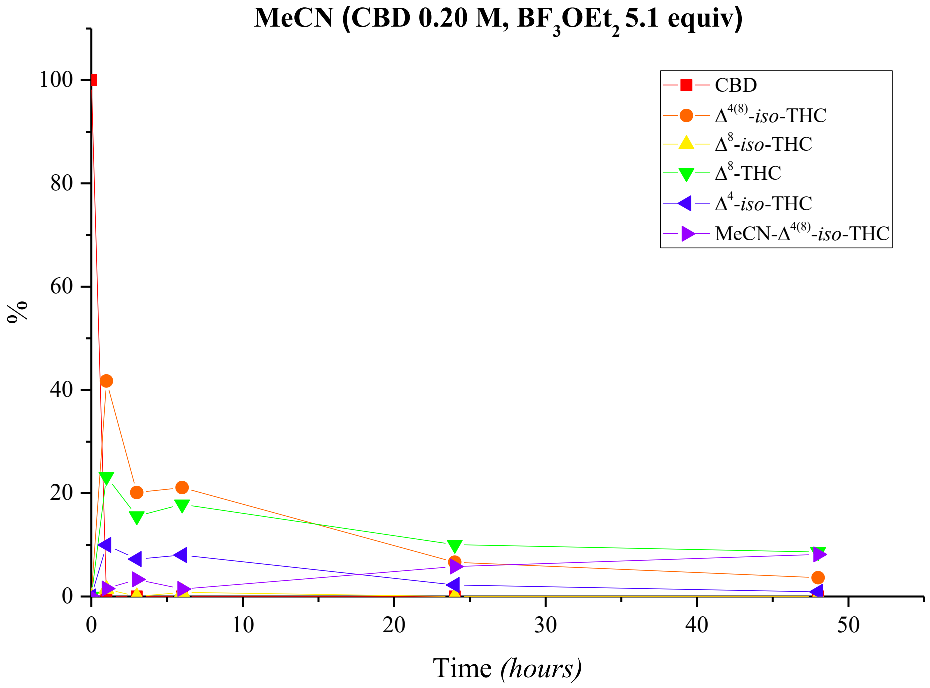

Fig. S32 *Time-dependent consumption of CBD and product formation in acetonitrile. Conditions: CBD 0.20 M, BF_3OEt_2 5.1 equivalents.*

As depicted in Figures S27-S32, in MeCN the formation $\Delta^{4(8)}$-iso-THC is followed by its partial conversion to the corresponding MeCN-$\Delta^{4(8)}$-iso-THC, that has been observed in high yield under the reaction conditions adopted in figure S28 (a 0.032 M solution of CBD treated with 5.1 equiv of BF_3OEt_2). In most cases, low amounts of Δ^8-THC and Δ^4-*iso*-THC have been also detected.

3. Design of Experiments (DoE)

3.1. 2^3 full factorial Design of Experiments

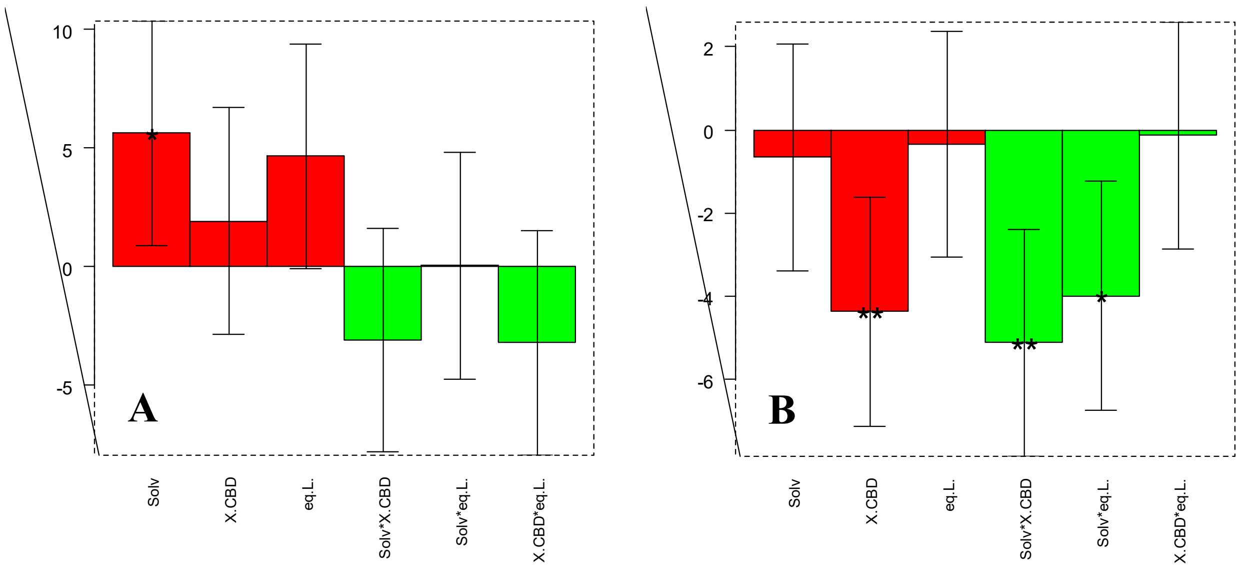

Fig. S33 *Coefficient plots relative to the FFD on $\Delta^{4(8)}$-iso-THC, respectively to [A] 1 hour of reaction time and [B] 3 hours of reaction time. The asterisks, when present, indicate a significant influence of the respective parameters or their interaction and significance (* $p \leq 0.05$, ** $p \leq 0.01$, *** $p \leq 0.001$).*

FFD on $\Delta^{4(8)}$-*iso*-THC yield				
Coefficient	***1 hour***		***3 hours***	
	Value	*Significance*	*Value*	*Significance*
b_0	32.4		31.1	
b_1	5.6	*	-0.7	
b_2	1.9		-4.4	**
b_3	4.7		-0.3	
b_{12}	-3.1		-5.1	**
b_{13}	0.0		-4.0	*
b_{23}	-3.2		-0.1	

Table S1 *Coefficient values and their significance (* $p \leq 0.05$, ** $p \leq 0.01$, *** $p \leq 0.001$ relative to the FFD on $\Delta^{4(8)}$-iso-THC.*

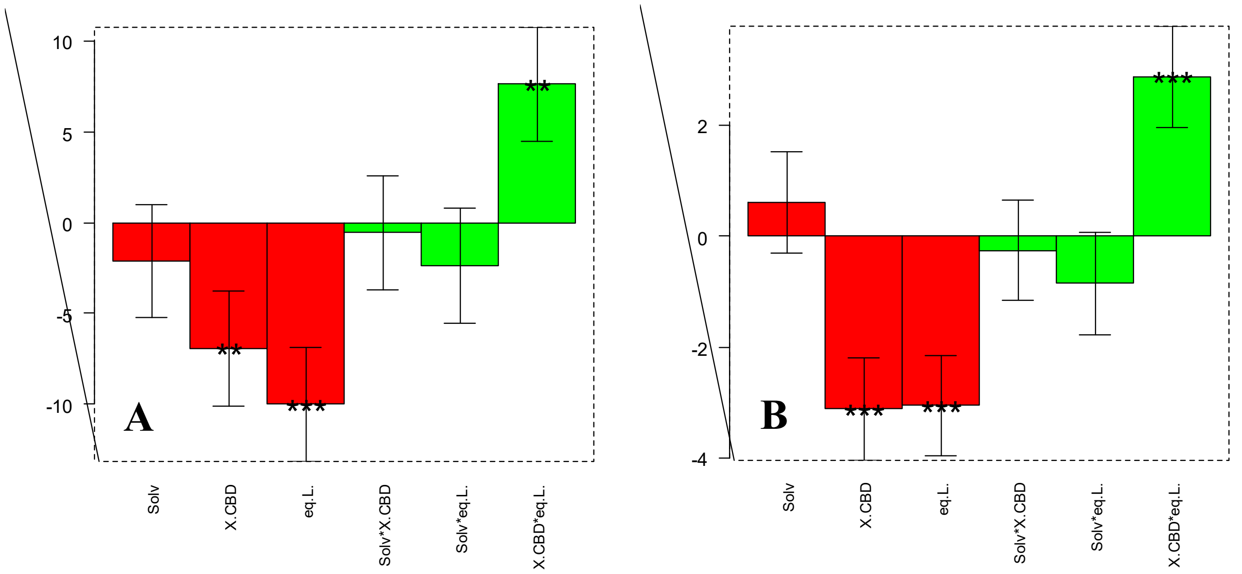

Fig. S34 *Coefficient plots relative to the FFD on Δ*[8]*-iso-THC, respectively to [A] 1 hour of reaction time and [B] 3 hours of reaction time. The asterisks, when present, indicate a significant influence of the respective parameters or their interaction and significance (* $p \leq 0.05$, ** $p \leq 0.01$, *** $p \leq 0.001$).*

FFD for the optimization of Δ^8-*iso*-THC yield				
Coefficient	***1 hour***		***3 hours***	
	Value	*Significance*	*Value*	*Significance*
b_0	14.9		3.5	
b_1	-2.1		0.6	
b_2	-6.9	**	-3.1	***
b_3	-10.0	***	-3.1	***
b_{12}	-0.6		-0.3	
b_{13}	-2.4		-0.9	
b_{23}	7.6	**	2.9	***

Table S2 *Coefficient values and their significance (* $p \leq 0.05$, ** $p \leq 0.01$, *** $p \leq 0.001$ relative to the FFD on Δ*[8]*-iso-THC.*

3.2. Face centered designs: validation, coefficients and their significance

	Δ$^{4(8)}$-iso-THC		***Δ^{8}-iso-THC***	
	Experimental	***Predicted***	***Experimental***	***Predicted***
Value	26.1	37.0	85.4	91.7
Lower limit	23.9	27.8	83.8	86.5
Upper limit	28.3	46.1	87.1	96.9

Table S3 *Summary of the model validation for the face-centered designs; the independent validation experiments for Δ$^{4(8)}$-iso-THC and Δ^{8}-iso-THC have been performed, respectively, at point [−0.5, –0.6] and [0.4, –0.5]. For both models, experimental yield – calculated with pooled standard deviation from central replicates – fell within the model's predicted confidence interval (95% confidence level), confirming the validity of the proposed models.*

FCD for the optimization of Δ$^{4(8)}$-*iso*-THC yield

Coefficient	***1 hour***		***2 hours***		***3 hours***	
	Value	*Significance*	*Value*	*Significance*	*Value*	*Significance*
b_0	26.1		18.0		12.6	
b_1	-8.1	***	-9.0	***	-7.8	***
b_2	-7.5	***	-3.4	*	-5.6	**
b_{12}	4.5	**	3.5	*	3.2	*
b_1^2	4.0	*	5.9	**	6.2	**
b_2^2	-0.1		-3.6	*	-0.7	

Table S4 *Optimization of Δ$^{4(8)}$-iso-THC yield by FCD: coefficient values and their significance (* p ≤ 0.05, ** p ≤ 0.01, *** p ≤ 0.001).*

FCD for the optimization of Δ^8-*iso*-THC yield

Coefficient	***1 hour***		***2 hours***		***3 hours***	
	Value	*Significance*	*Value*	*Significance*	*Value*	*Significance*
b_0	90.7		85.7		80.4	
b_1	0.2		-5.8	***	-9.3	***
b_2	-5.2	***	-18.7	***	-17.9	***
b_{12}	-11.9	***	-11.5	***	-13.5	***
b_1^2	-9.3	***	-6.5	**	-5.6	**
b_2^2	-10.6	***	-12.8	***	-10.6	***

Table S5 *Optimization of Δ^8-iso-THC yield by FCD: coefficient values and their significance (* $p \leq 0.05$, ** $p \leq 0.01$, *** $p \leq 0.001$).*

4. ^{1}H and ^{13}C NMR Spectra for the isolated compounds

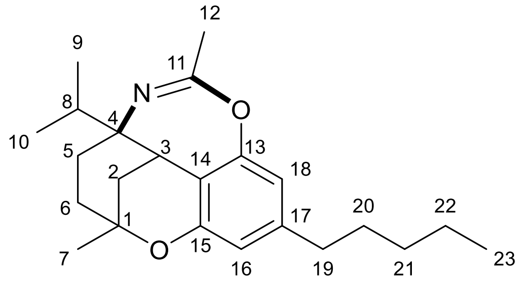

MeCN -Δ4(8)-*iso*-THC

MeCN-Δ4(8)-*iso*-THC. ^{1}H NMR (400 MHz, CD_3COCD_3) δ 6.41 (d, *J* = 1.5 Hz, 1H), 6.33 (d, *J* = 1.5 Hz, 1H), 3.66 (dt, *J* = 4.1, 1.7 Hz, 1H, H-3), 2.56 – 2.40 (m, 2H, H-19), 2.24 (p, *J* = 6.7 Hz, 1H, H-8), 2.14 (dd, *J* = 13.8, 2.0 Hz, 1H, H-2), 2.02 (s, 3H, H-12), 1.83 – 1.65 (m, 4H, H-5, H-6, H-2), 1.59 (p, *J* = 7.5 Hz, 2H, H-20), 1.40 – 1.26 (m, 7H, H-7, H-21, H-22), 1.19 – 1.08 (m, 1H, H-5), 1.01 (d, *J* = 6.5 Hz, 3H, H-10), 0.90 (m, 6H, H-9 and H-23). ^{13}C NMR (101 MHz, CD_3COCD_3) δ 158.0, 156.3, 149.7 (C-11), 143.6, 116.7, 112.2 (CH), 110.6 (CH), 74.8 (C-1), 63.2 (C-4), 36.7 (CH_2, C-19), 36.3 (CH_2, C-6), 35.8 (CH, C-3), 32.9 (CH_2, C-2), 32.6 (CH_2, C-21), 32.1 (CH_2, C-20), 30.7 (CH_2, C-5), 30.5 (CH, C-8), 29.3 (CH_2, C-22), 24.8 (CH_3, C-12), 23.5 (CH_3, C-7), 18.0 (CH_3, C-10), 17.3 (CH_3, C-9), 14.7 (CH_3, C-23).

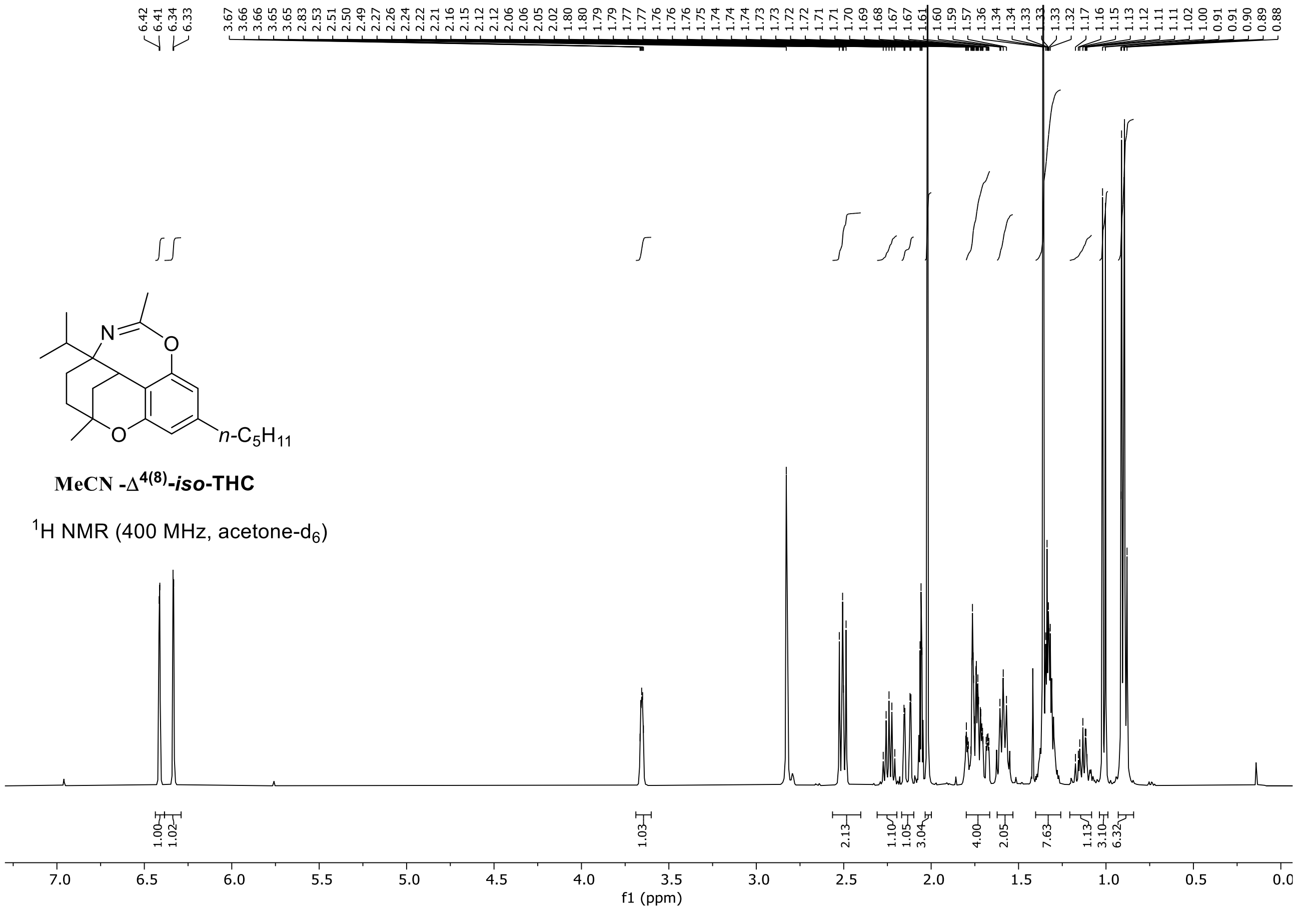

MeCN -Δ$^{4(8)}$-*iso*-THC

^{1}H NMR (400 MHz, acetone-d_6)

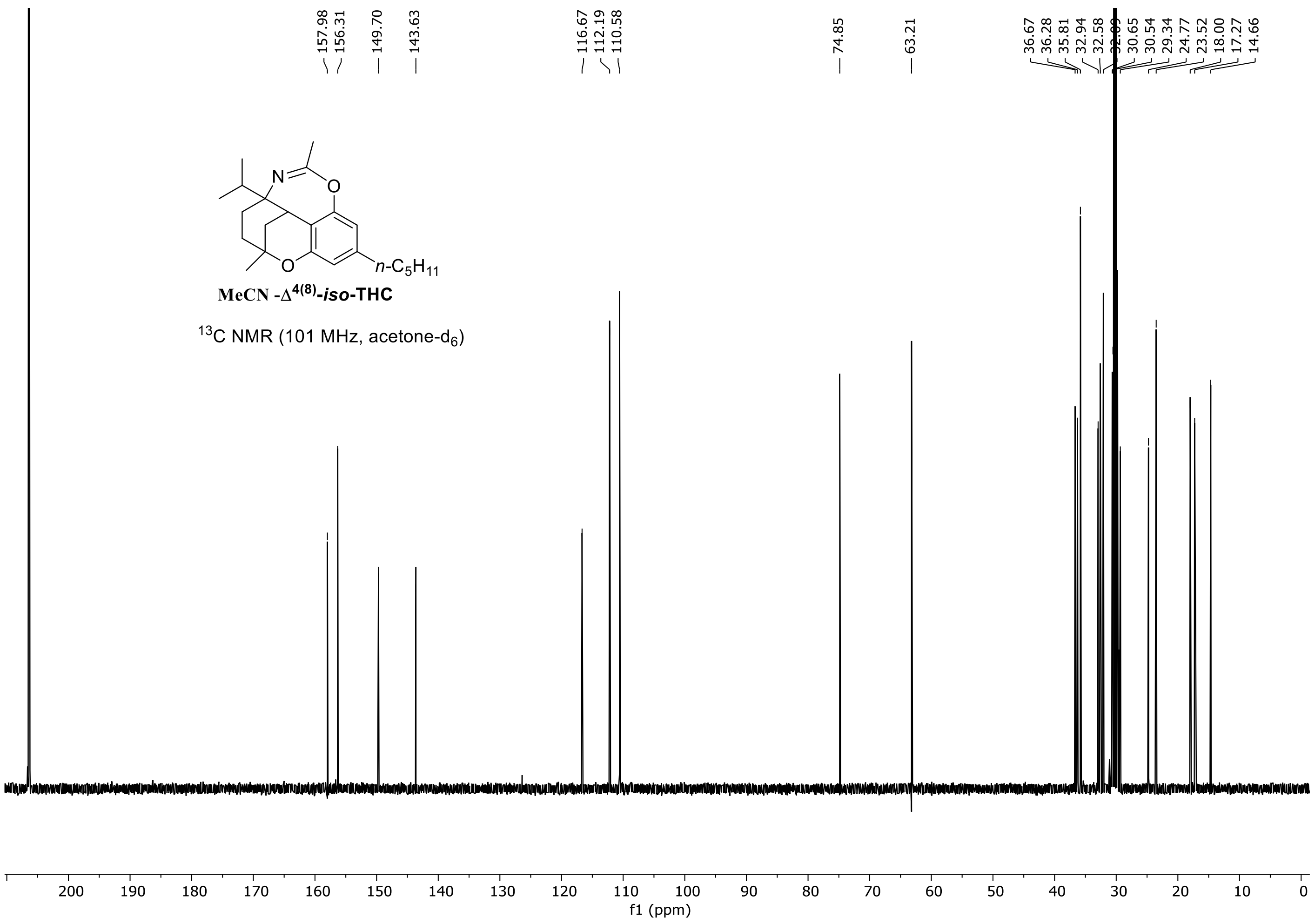
157.98
156.31
149.70
143.63
116.67
112.19
110.58
74.85
63.21
36.67
36.28
35.81
32.94
32.58
30.65
30.54
29.34
24.77
23.52
18.00
17.27
14.66
N
O
O
n-C5H11
MeCN -Δ4(8)-iso-THC
13C NMR (101 MHz, acetone-d6)
200
190
180
170
160
150
140
130
120
110
100
90
80
70
60
50
40
30
20
10
0
f1 (ppm)

Heteronuclear single quantum coherence (HSQC) spectra for compound MeCN-Δ$^{4(8)}$-*iso*-THC (Acetone-d$_6$)

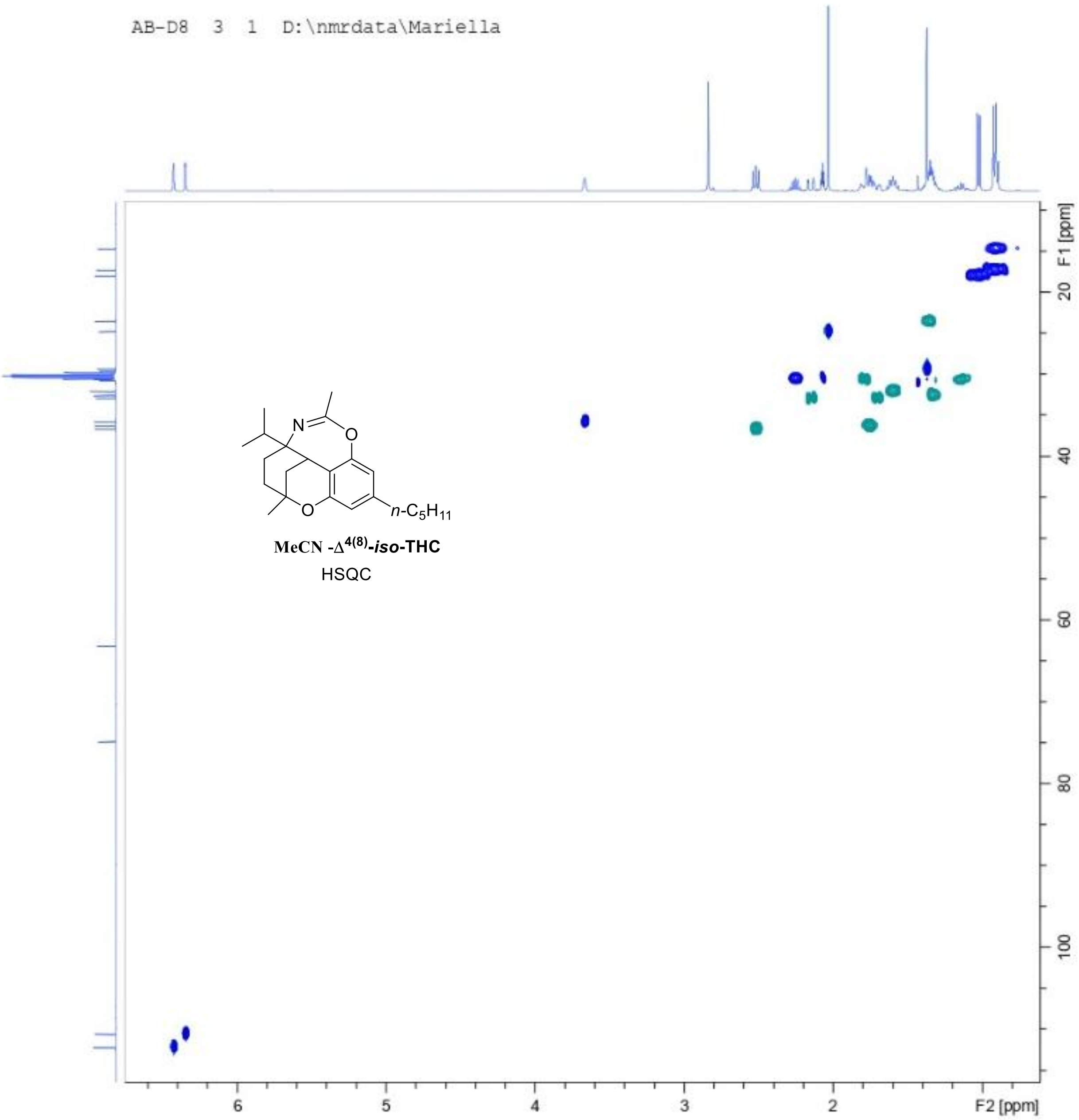

Heteronuclear Multiple Bond Correlation (HMBC) spectra for compound MeCN-Δ$^{4(8)}$-*iso*-THC (Acetone-d$_6$)

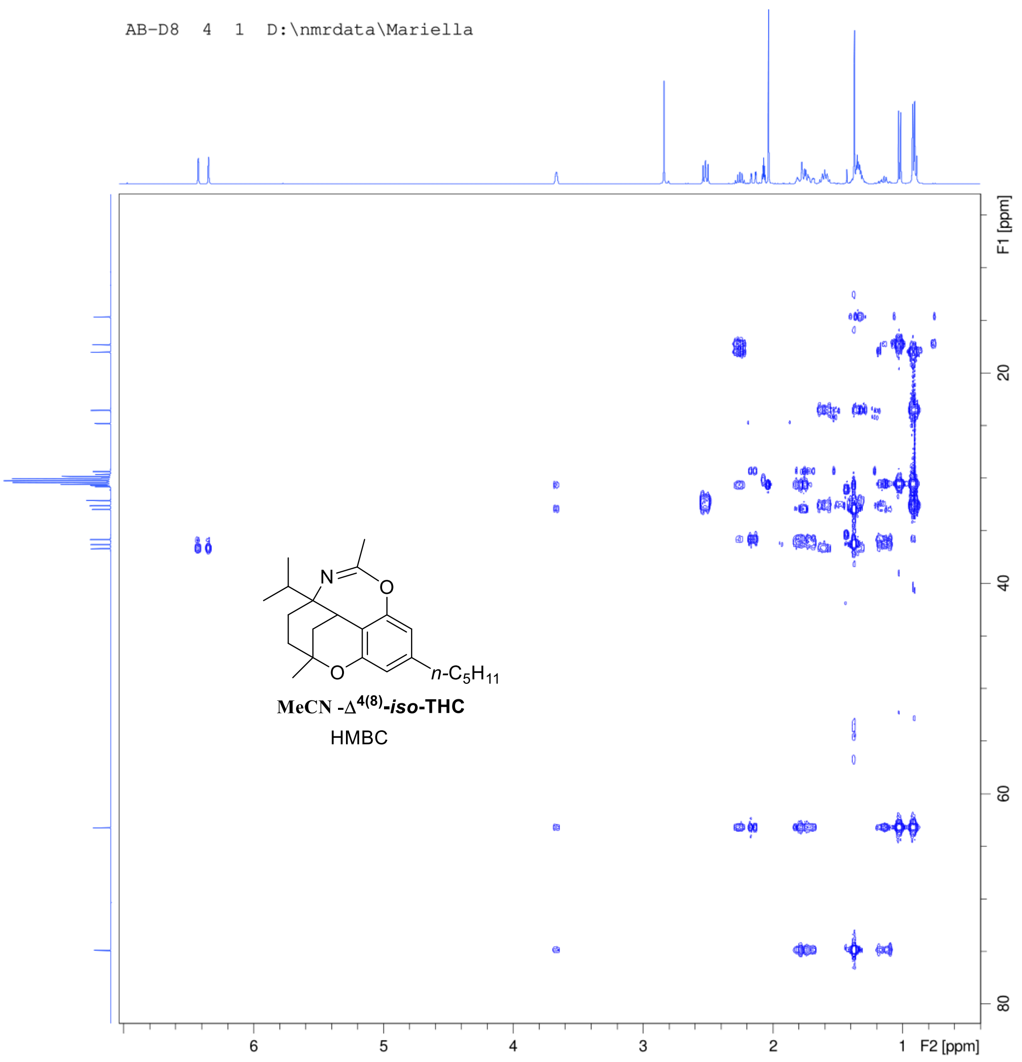

Correlation Spectroscopy analysis (COSY) for compound MeCN-$\Delta^{4(8)}$-iso-THC (Acetone-d_6)

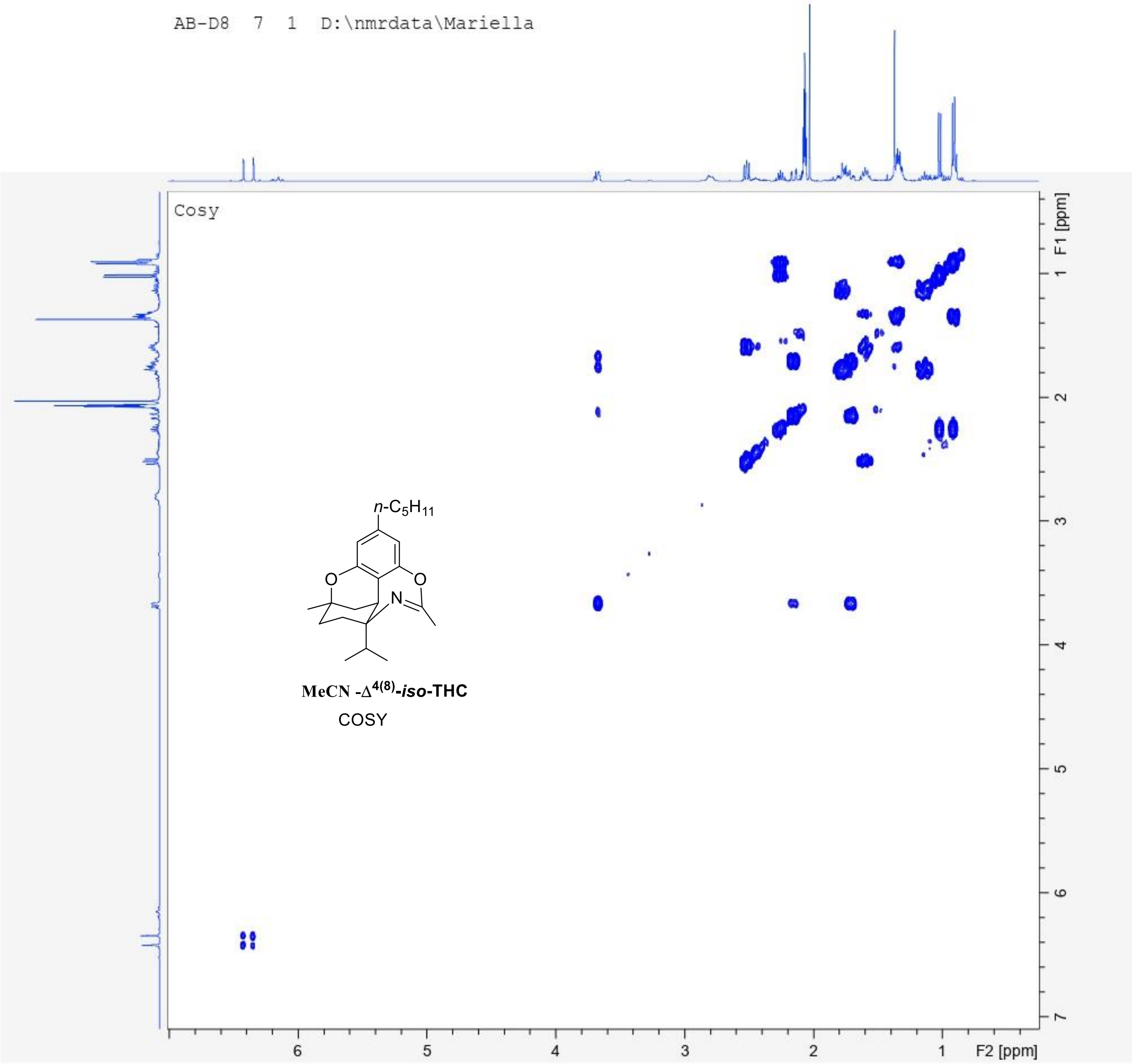

Nuclear Overhauser Effect Spectrum (NOESY) for compound MeCN-$\Delta^{4(8)}$-iso-THC (Acetone-d_6)

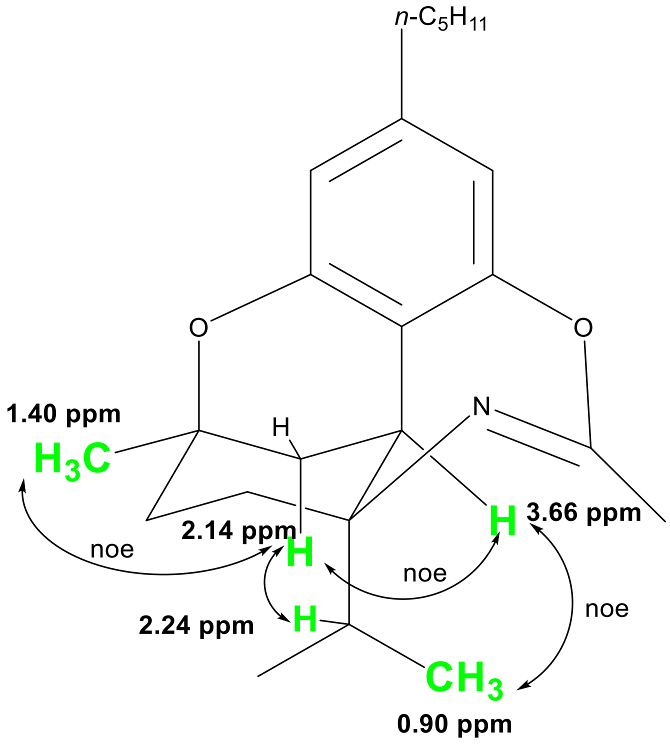

Nuclear Overhauser Effect Spectrum (NOESY) for compound MeCN-$\Delta^{4(8)}$-iso-THC (Acetone-d_6)

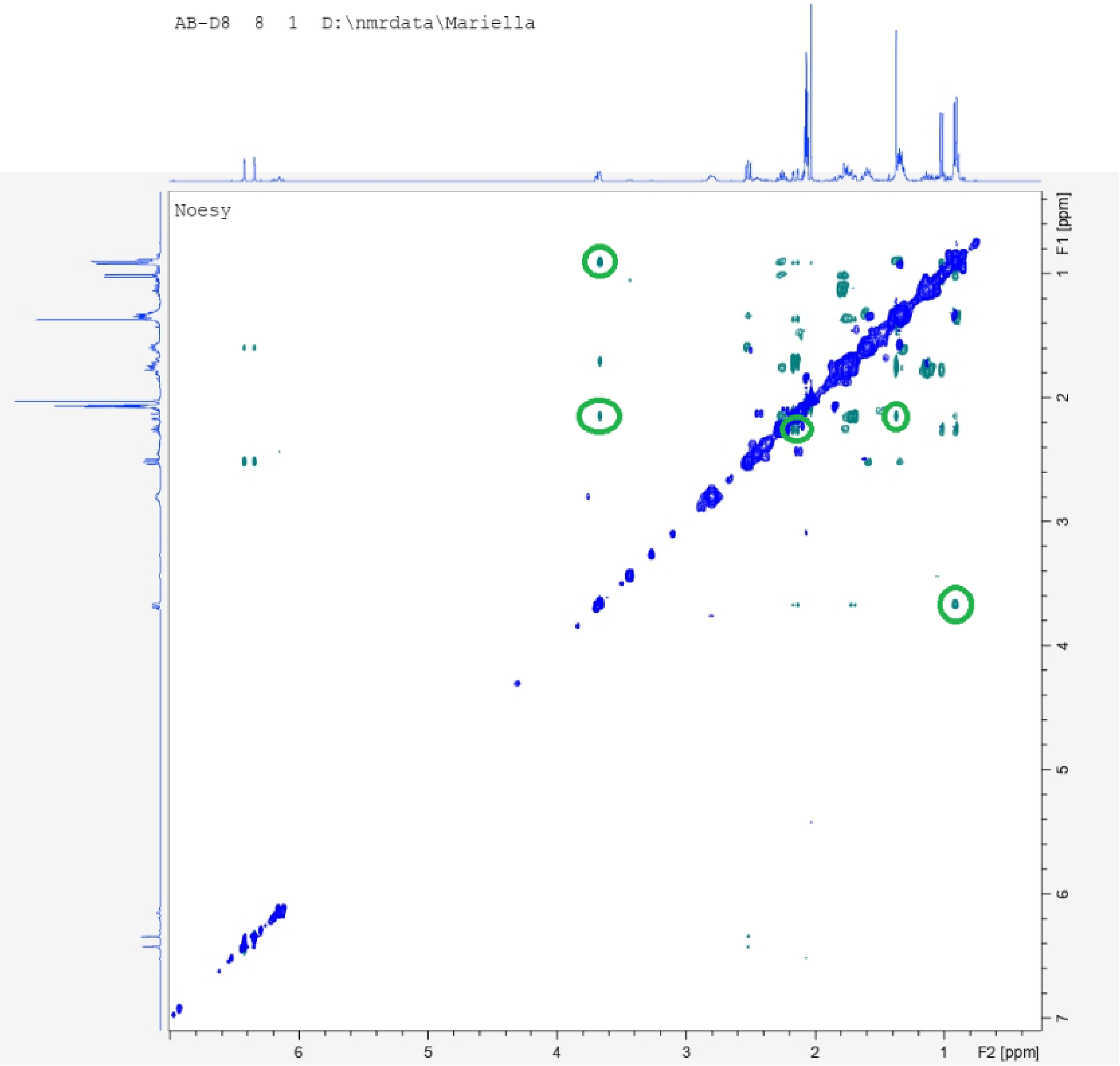

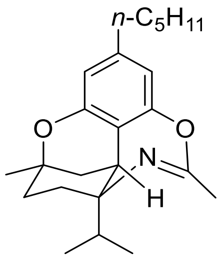

MeCN -Δ$^{4(8)}$-*iso*-THC

NOESY + COSY

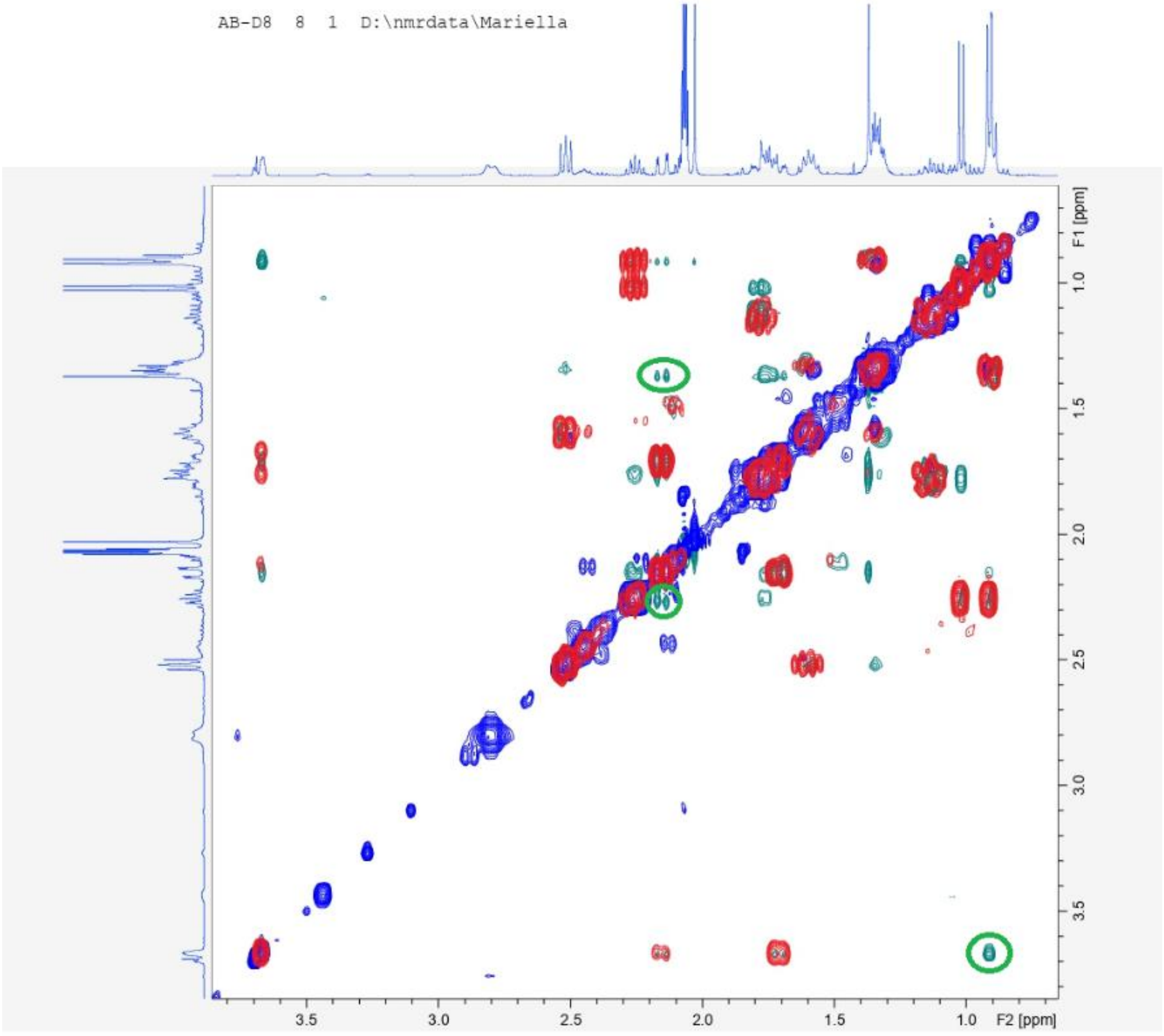

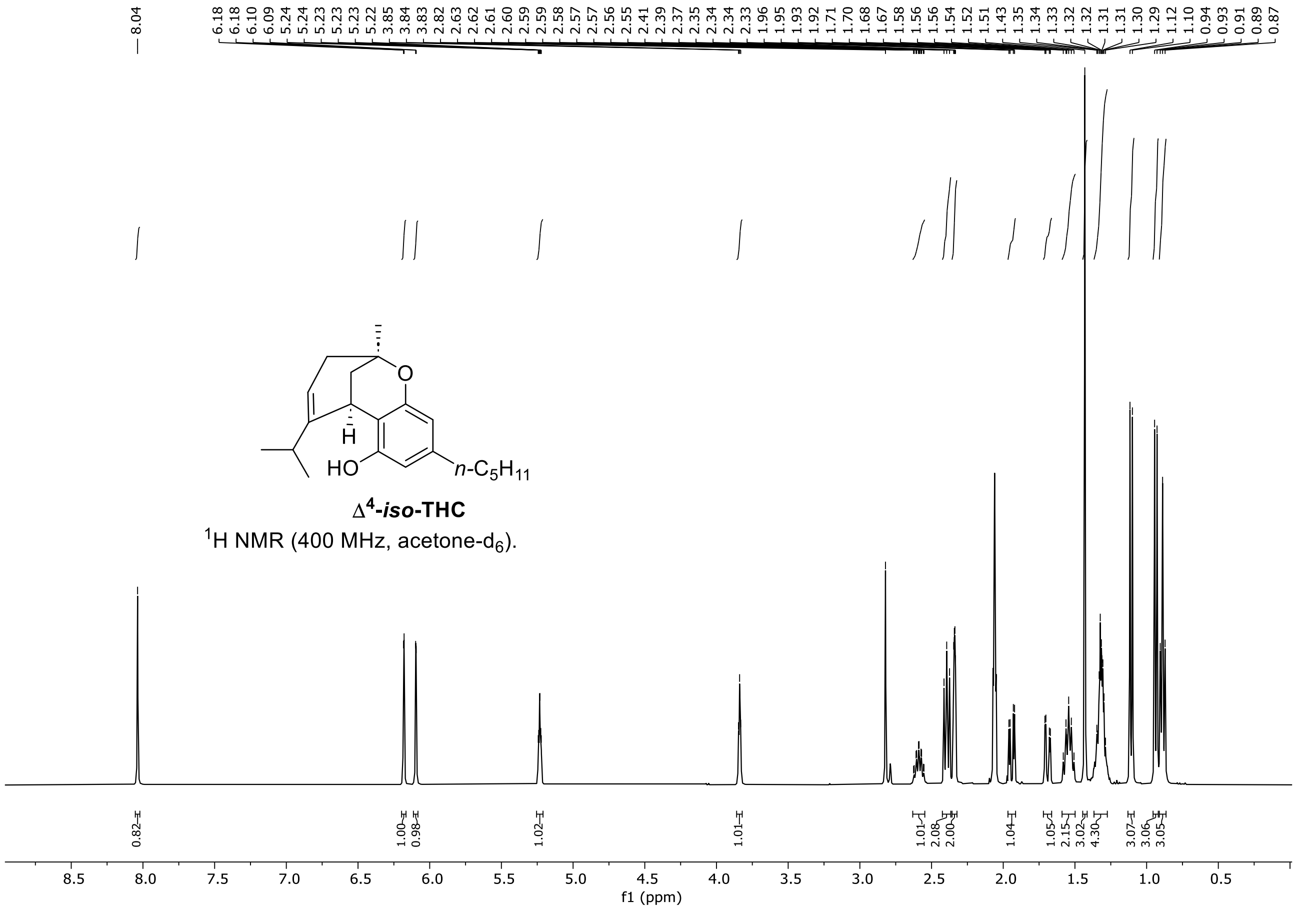
HO
H
O
n-C5H11
Δ4-iso-THC
1H NMR (400 MHz, acetone-d6).
f1 (ppm)

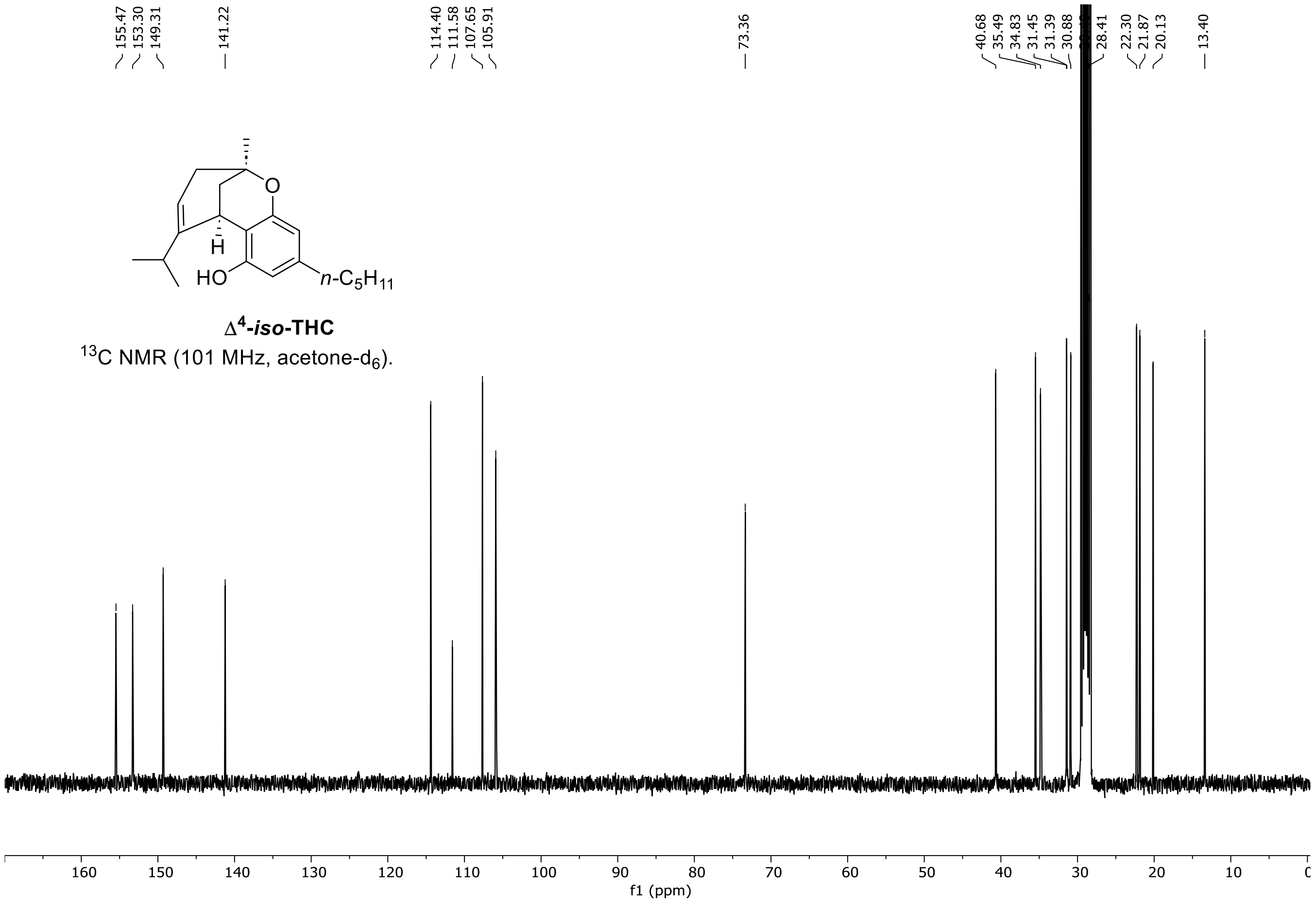
155.47
153.30
149.31
141.22
114.40
111.58
107.65
105.91
73.36
40.68
35.49
34.83
31.45
31.39
30.88
28.41
22.30
21.87
20.13
13.40
O
H
HO
n-C5H11
Δ4-iso-THC
13C NMR (101 MHz, acetone-d6).
160
150
140
130
120
110
100
90
80
70
60
50
40
30
20
10
f1 (ppm)

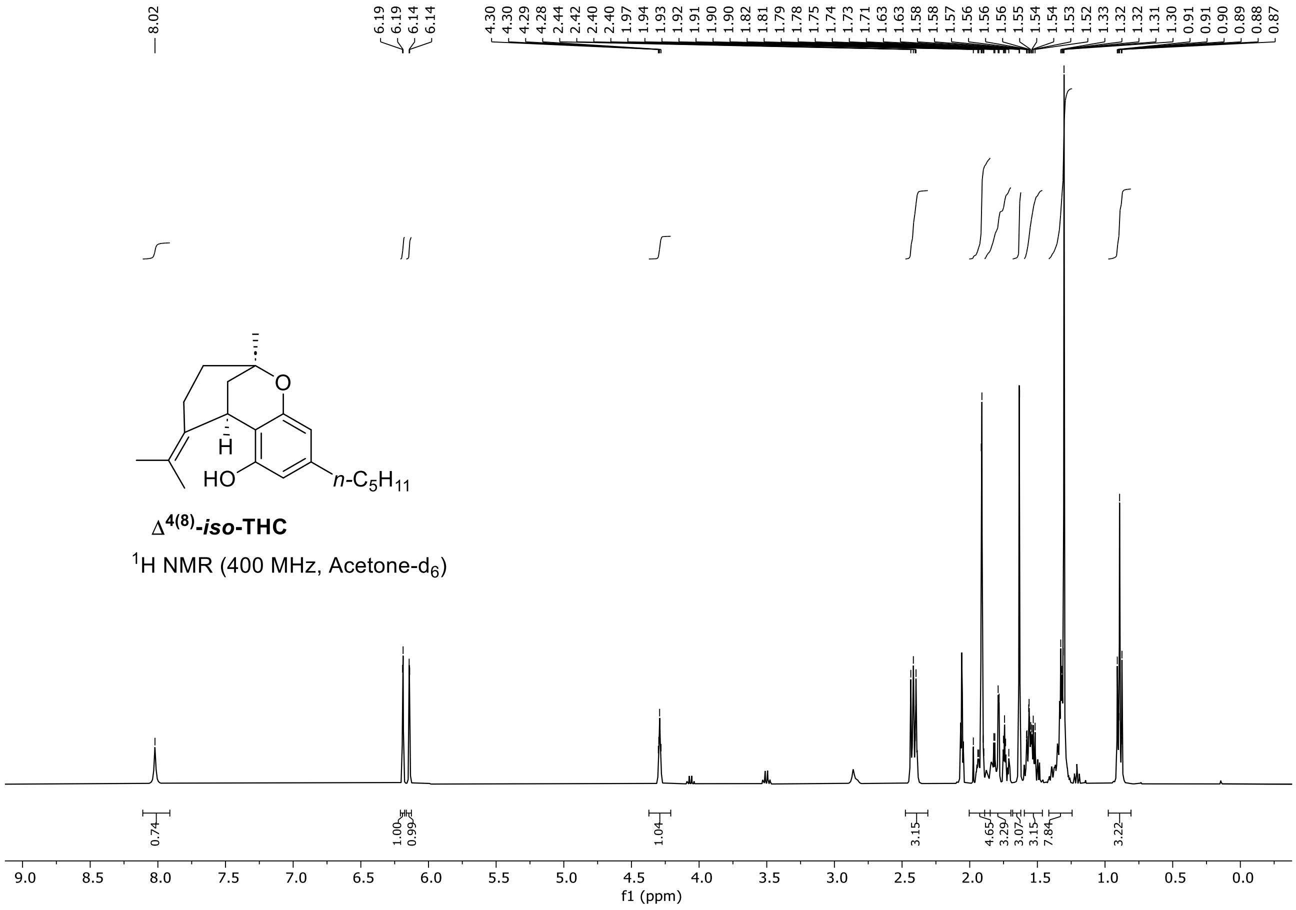

$\Delta^{4(8)}$-*iso*-THC

^1H NMR (400 MHz, Acetone-d_6)

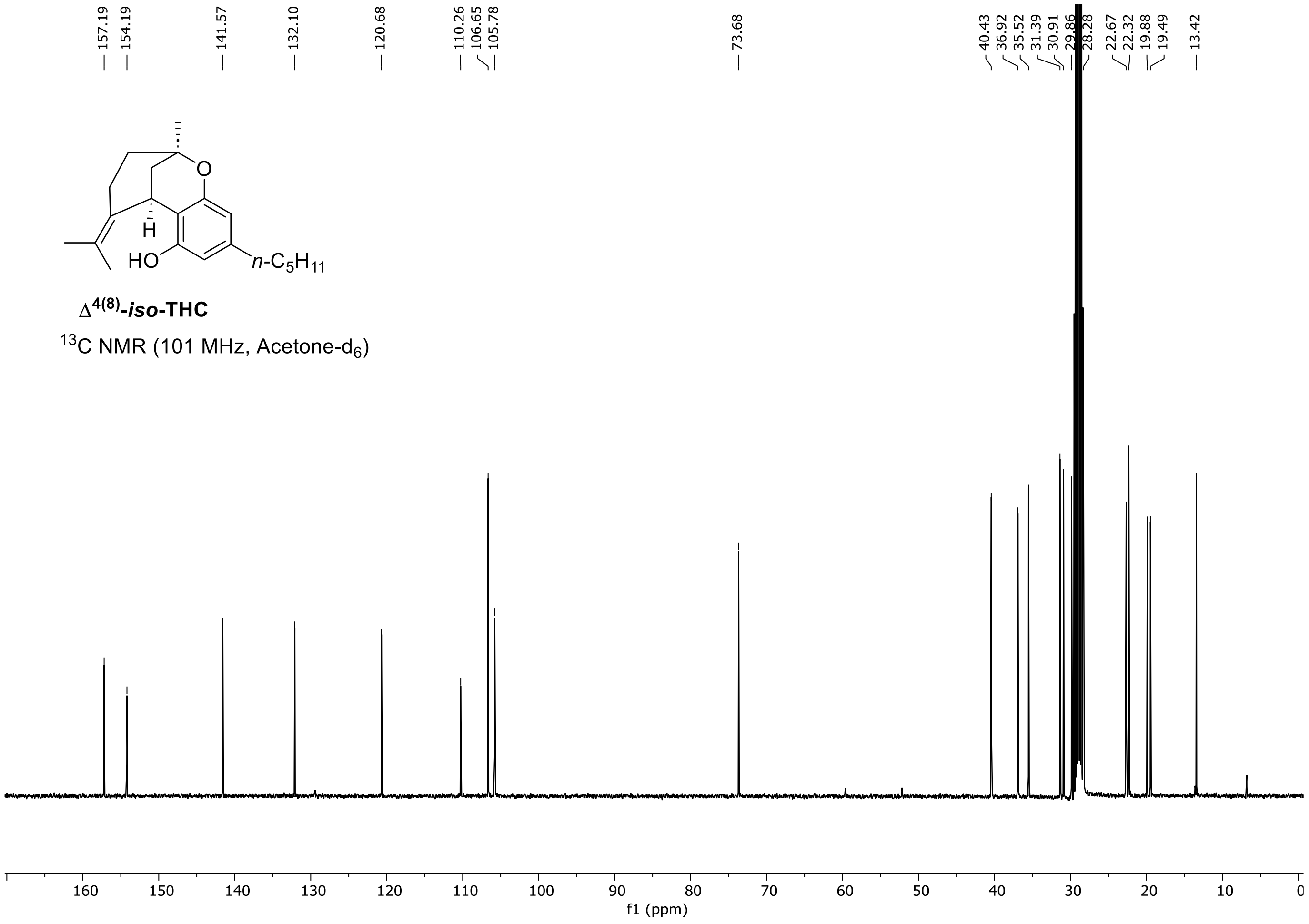
157.19
154.19
141.57
132.10
120.68
110.26
106.65
105.78
73.68
40.43
36.92
35.52
31.39
30.91
29.86
28.28
22.67
22.32
19.88
19.49
13.42
O
H
HO
n-C_5H_{11}
$\Delta^{4(8)}$-*iso*-THC
^{13}C NMR (101 MHz, Acetone-d_6)
160
150
140
130
120
110
100
90
80
70
60
50
40
30
20
10
0
f1 (ppm)

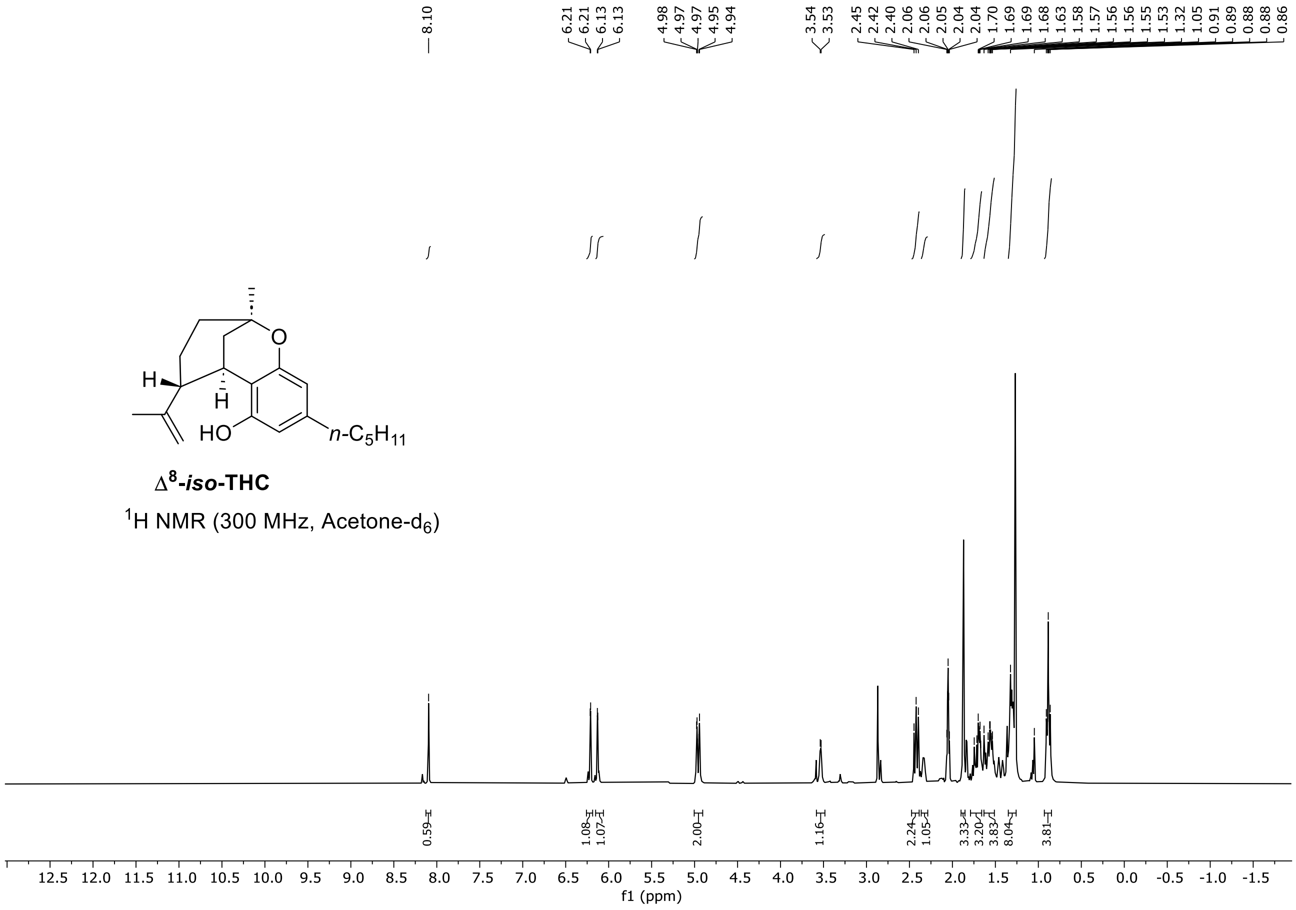

Δ[8]-*iso*-THC

^{1}H NMR (300 MHz, Acetone-d$_6$)

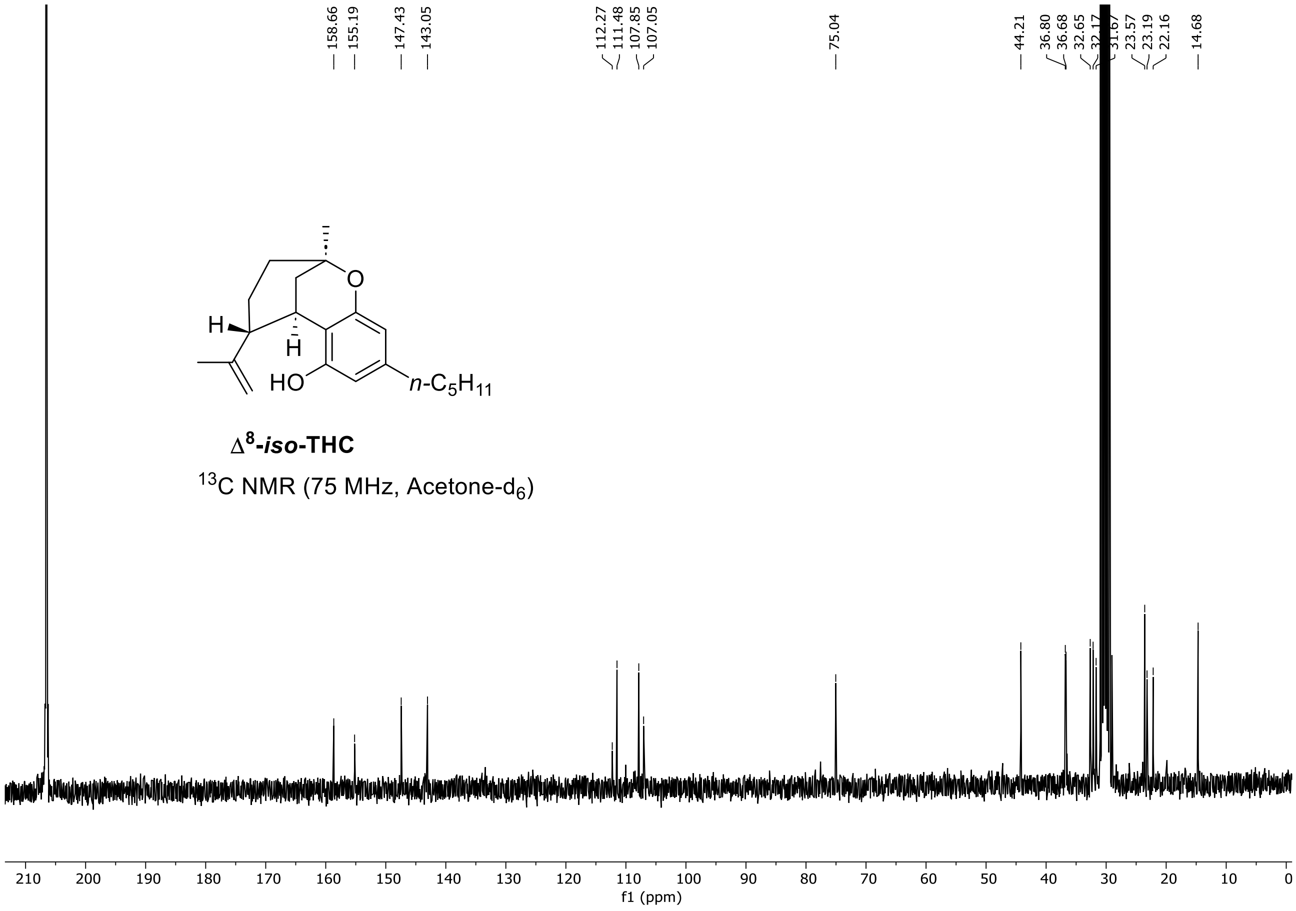
158.66
155.19
147.43
143.05
112.27
111.48
107.85
107.05
75.04
44.21
36.80
36.68
32.65
32.17
31.67
23.57
23.19
22.16
14.68
H
H
O
HO
n-C5H11
Δ8-iso-THC
13C NMR (75 MHz, Acetone-d6)
210 200 190 180 170 160 150 140 130 120 110 100 90 80 70 60 50 40 30 20 10 0
f1 (ppm)

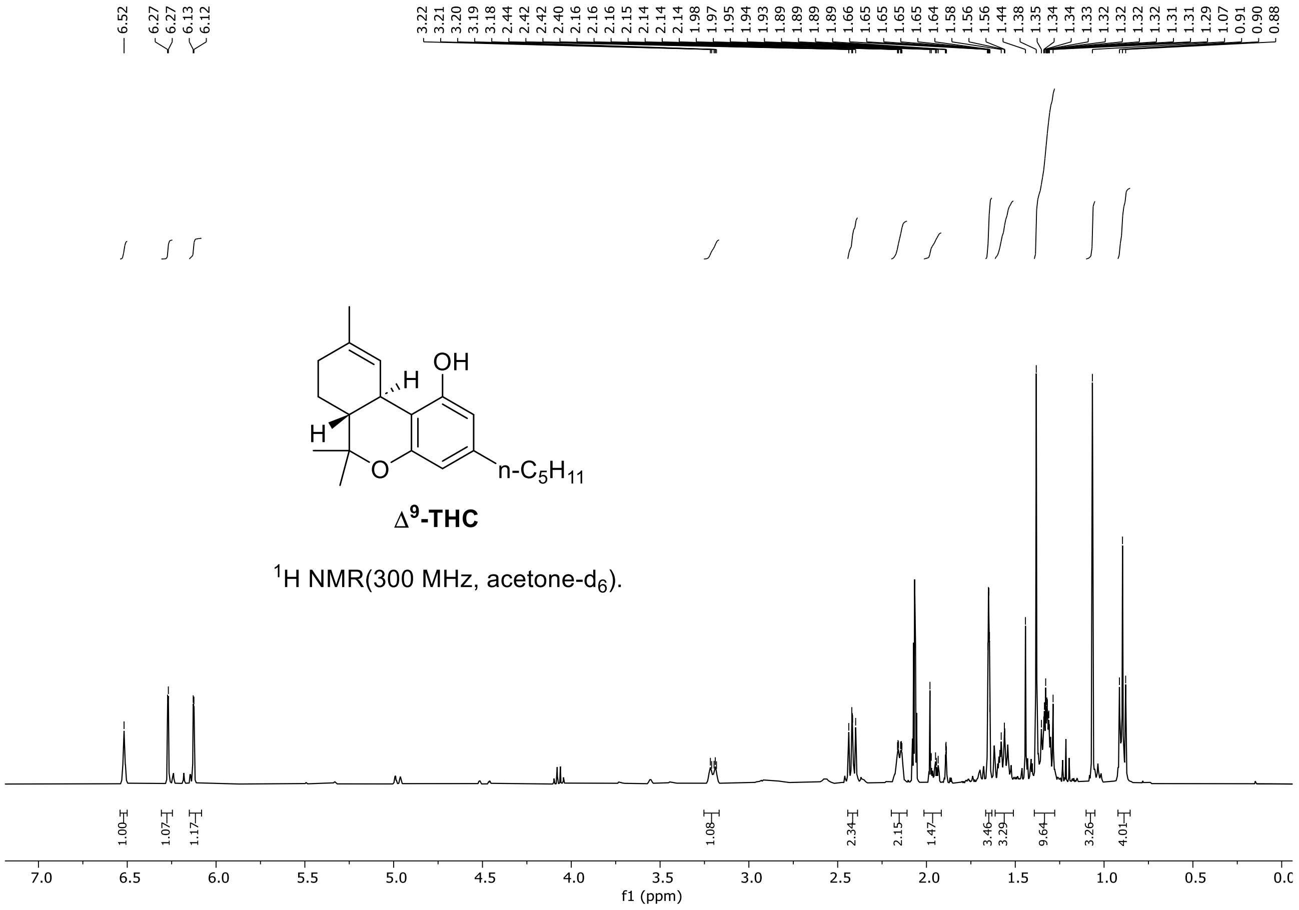
6.52
6.27 6.27 6.13 6.12
3.22 3.21 3.20 3.19 3.18 2.44 2.42 2.42 2.40 2.16 2.16 2.16 2.15 2.14 2.14 2.14 1.98 1.97 1.95 1.94 1.93 1.89 1.89 1.89 1.89 1.66 1.65 1.65 1.65 1.65 1.64 1.58 1.56 1.56 1.44 1.38 1.35 1.34 1.34 1.33 1.32 1.32 1.32 1.32 1.31 1.31 1.29 1.07 0.91 0.90 0.88
OH
H
H
O
n-C5H11
Δ9-THC
1H NMR(300 MHz, acetone-d6).
1.00 1.07 1.17 1.08 2.34 2.15 1.47 3.46 3.29 9.64 3.26 4.01
7.0 6.5 6.0 5.5 5.0 4.5 4.0 3.5 3.0 2.5 2.0 1.5 1.0 0.5 0.0
f1 (ppm)

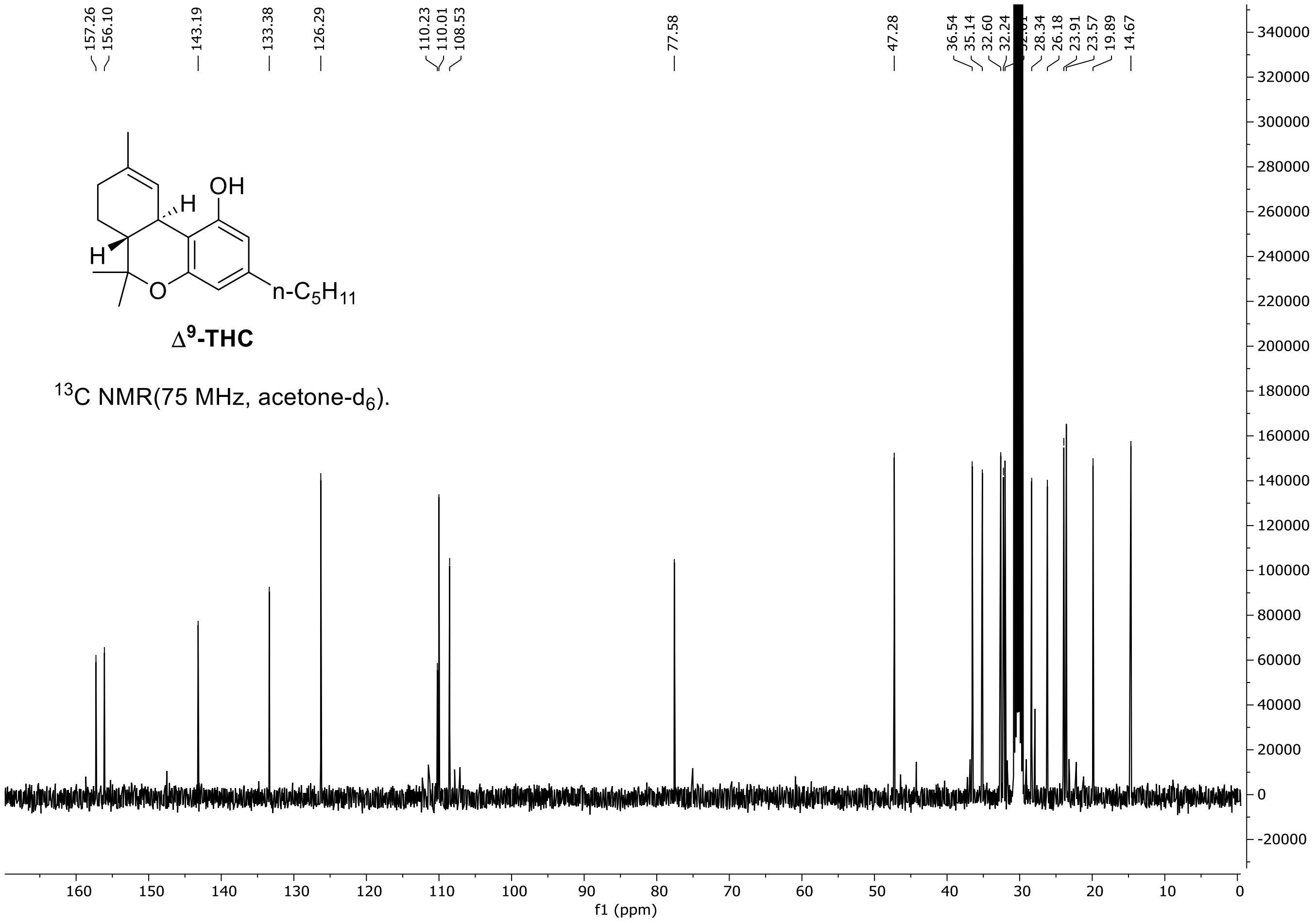

157.26
156.10
143.19
133.38
126.29
110.23
110.01
108.53
77.58
47.28
36.54
35.14
32.60
32.24
28.34
26.18
23.91
23.57
19.89
14.67
OH
H
H
O
n-C5H11
Δ9-THC
13C NMR(75 MHz, acetone-d6).
f1 (ppm)

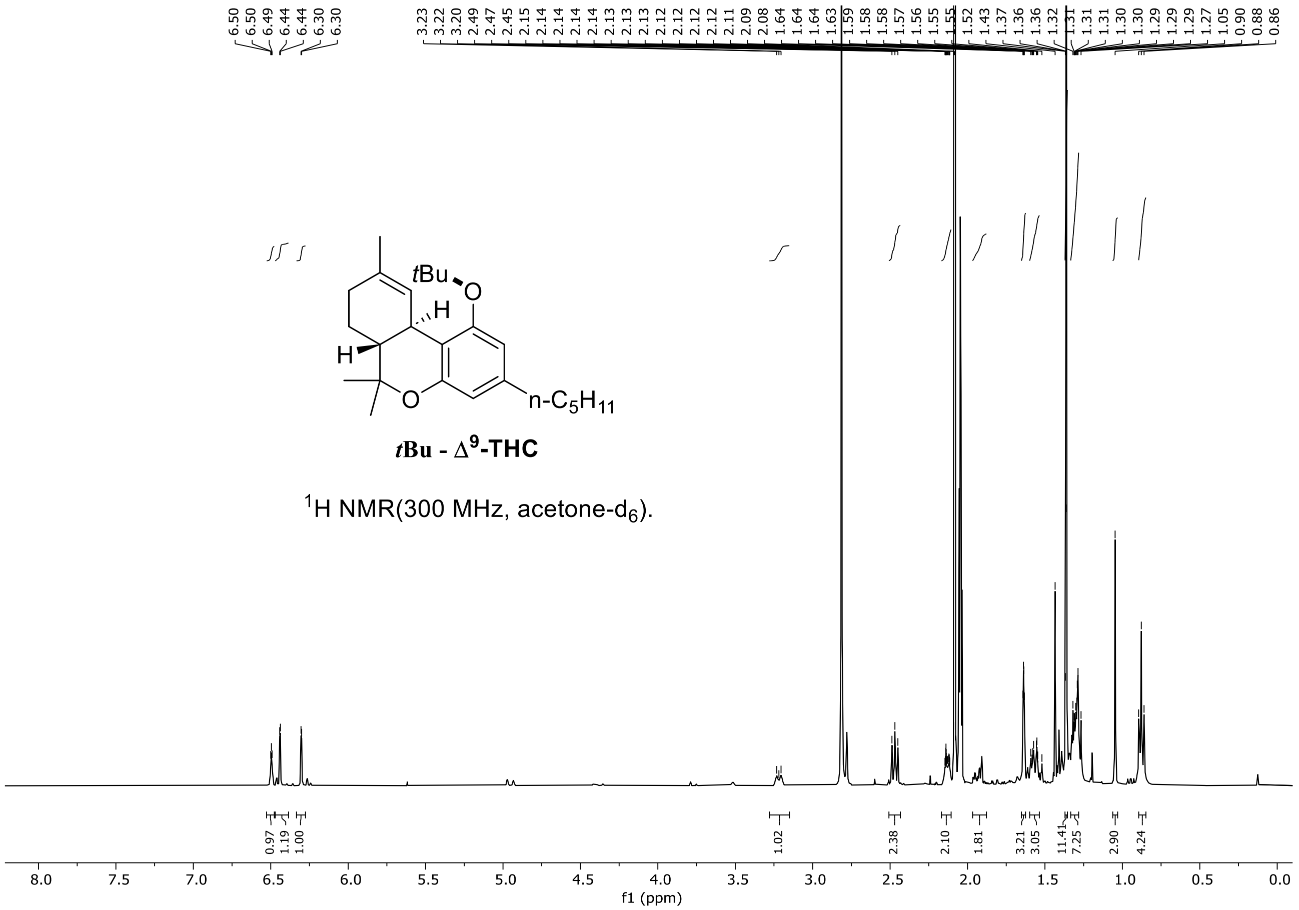
tBu - Δ9-THC
1H NMR(300 MHz, acetone-d6).
tBu
O
H
H
O
n-C5H11
f1 (ppm)

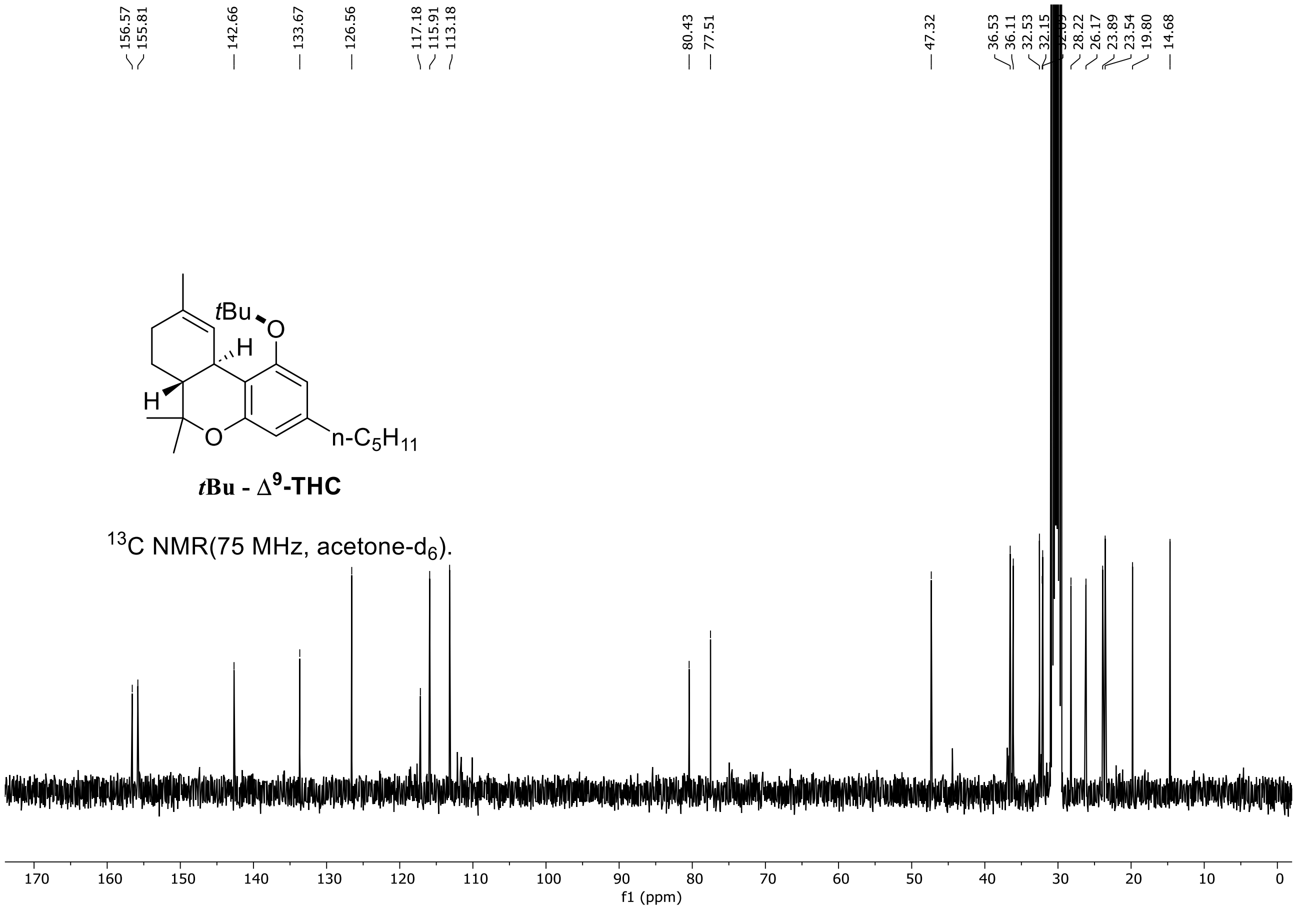
156.57
155.81
142.66
133.67
126.56
117.18
115.91
113.18
80.43
77.51
47.32
36.53
36.11
32.53
32.15
28.22
26.17
23.89
23.54
19.80
14.68
tBu
O
H
H
O
n-C5H11
tBu - Δ9-THC
13C NMR(75 MHz, acetone-d6).
f1 (ppm)

5. Exact mass results

MeCN -Δ^{4(8)}-iso-THC, HRMS *m/z*: $[M+H]^+$ calcd for $C_{23}H_{33}O_2N$ 356.2584; found 356.2581.

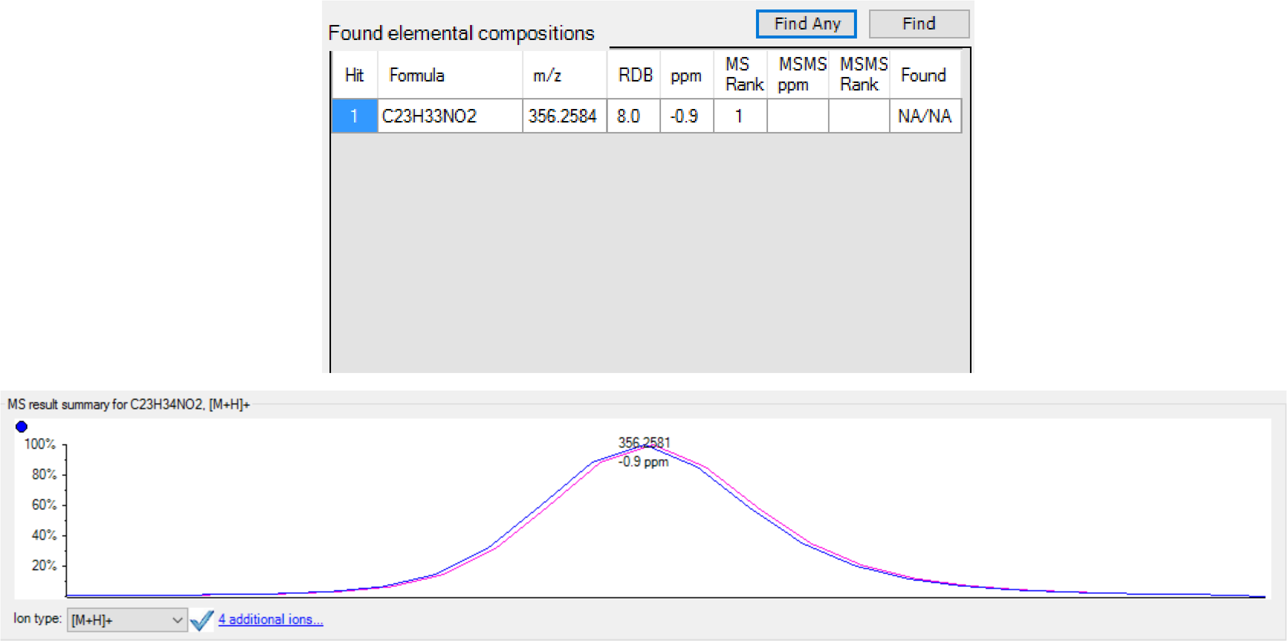

Found elemental compositions

Hit	Formula	m/z	RDB	ppm	MS Rank	MSMS ppm	MSMS Rank	Found
1	C23H33NO2	356.2584	8.0	-0.9	1			NA/NA

Fig. S35 *HRMS peak obtained from the analysis of isolated* ***MeCN -Δ^{4(8)}-iso-THC.***

Δ^4-iso-THC, HRMS *m/z*: $[M+H]^+$ calcd for $C_{21}H_{30}O_2$ 315.2319, found 315.2310

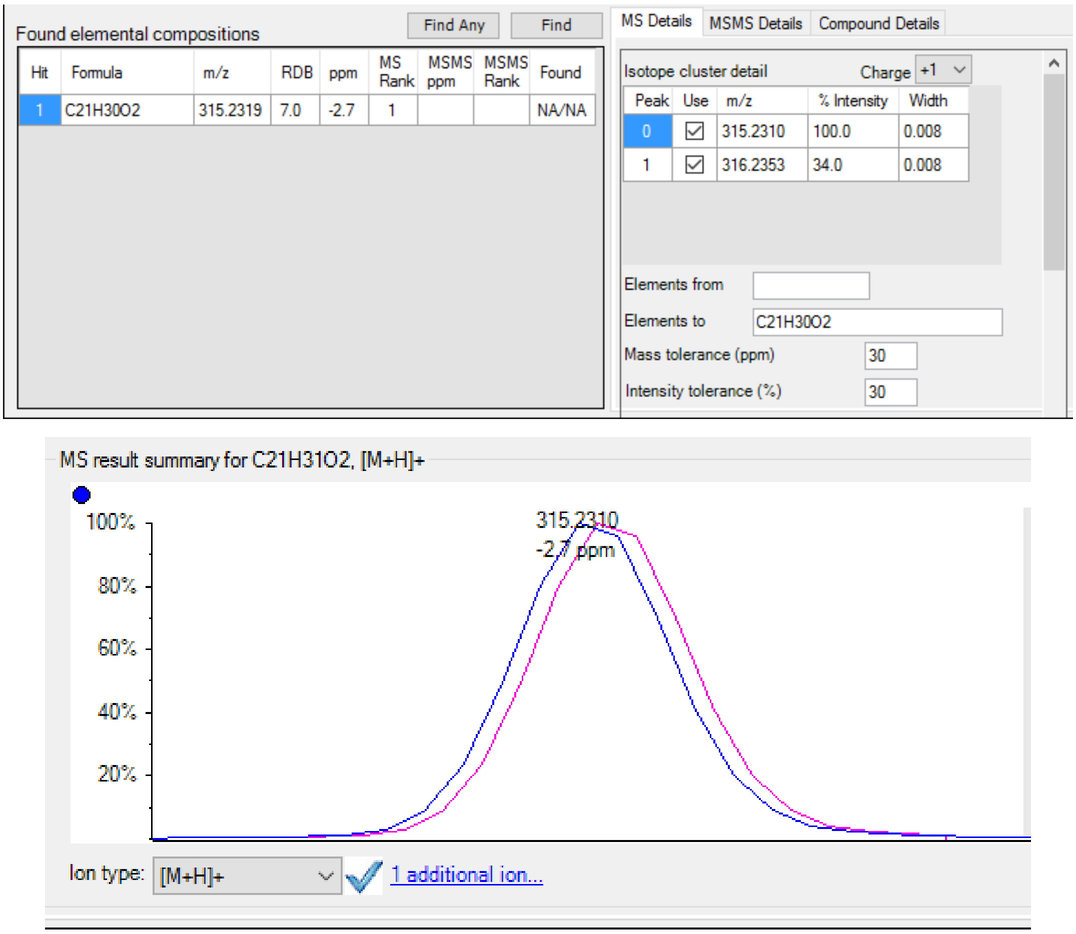

***Fig. S36** HRMS peak obtained from the analysis of isolated **Δ^4-iso-THC**.*

Δ**$^{4(8)}$**-iso-THC, HRMS *m/z*: $[M+H]^+$ calcd for $C_{21}H_{30}O_2$ 315.2319; found 315.2316

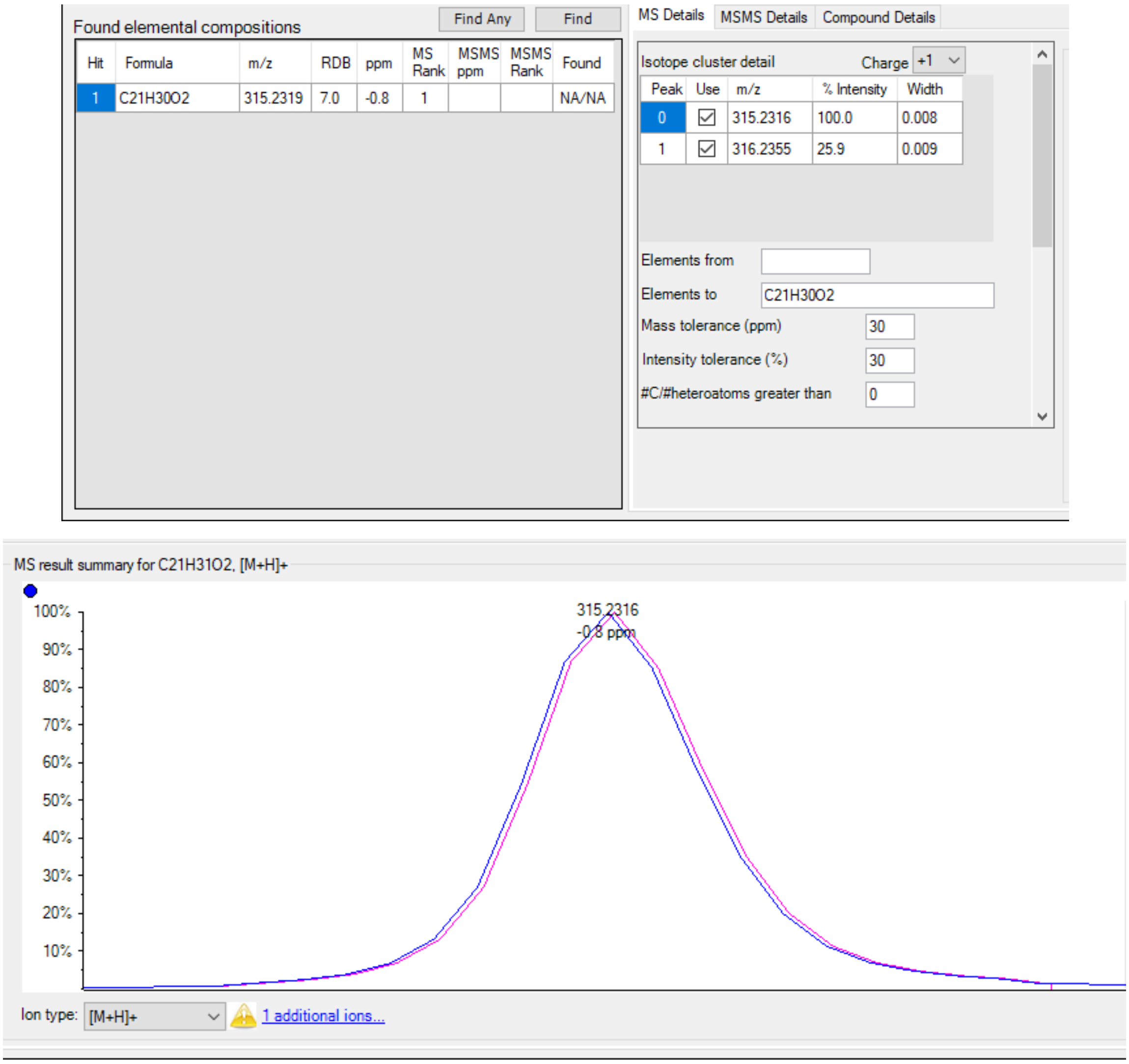

Fig. S37 *HRMS peak obtained from the analysis of isolated* ***Δ**$^{4(8)}$**-iso-THC***.

1-(tert-butoxy)-6,6,9-trimethyl-3-pentyl-6a,7,8,10a-tetrahydro-6H-benzo[c]chromene (tBu-$\Delta^{4(8)}$-iso-THC), HRMS *m/z*: $[M+H]^+$ calcd for $C_{25}H_{39}O_2$ 371.2945, found 371.2940

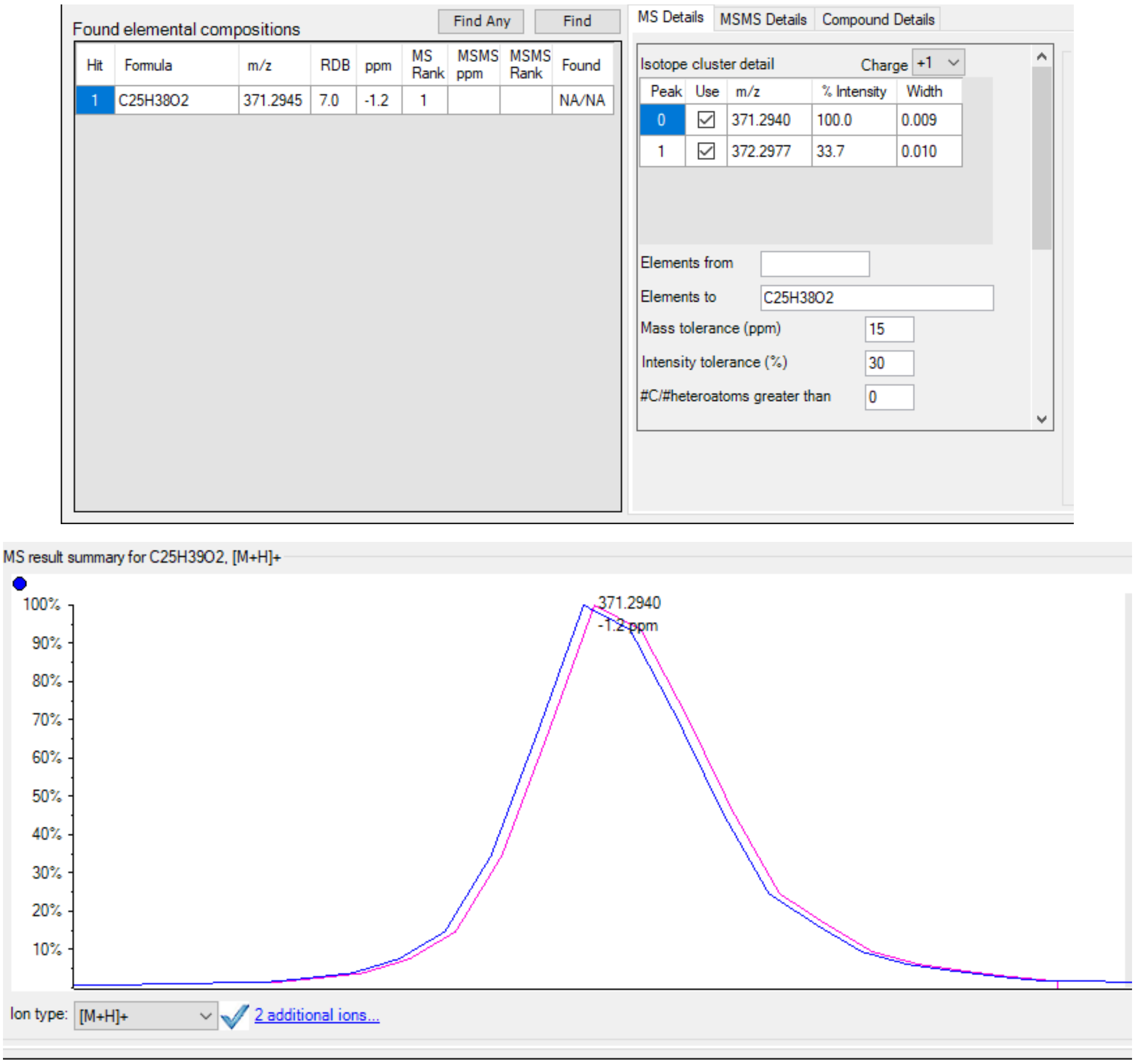

Fig. S38 *HRMS peak obtained from the analysis of isolated* ***tBu-$\Delta^{4(8)}$-iso-THC****.*
